# Anti-Cancer Effect of Sesquiterpene and Triterpenoids from Agarwood of *Aquilaria sinensis*

**DOI:** 10.3390/molecules27165350

**Published:** 2022-08-22

**Authors:** Lili Chen, Yunyun Liu, Yifei Li, Wu Yin, Yongxian Cheng

**Affiliations:** 1 State Key Lab of Pharmaceutical Biotechnology, College of Life Sciences, Nanjing University, Nanjing 210023, China; 2 Health Science Center, Institute for Inheritance-Based Innovation of Chinese Medicine, School of Pharmaceutical Sciences, Shenzhen University, Shenzhen 518060, China

**Keywords:** *Aquilaria sinensis*, agarwood, sesquiterpenes, cucurbitacins, anti-cancer

## Abstract

Two new guaiane sesquiterpenes, aquisinenoids A and B (**1** and **2**), two new eudesmane-type sesquiterpenoids, aquisinenoids C and D (**3** and **4**), one new cucurbitacin, aquisinenoid E (**5**), and five known cucurbitacins (**6**–**10**) were isolated from agarwood of *Aquilaria sinensis*. The structures of these new compounds, including their absolute configurations, were characterized by spectroscopic and computational methods. The biological evaluation showed that compounds **3** and **9** had an anti-cancer effect on most of the cancer cells at 5 μM, especially in human breast cancer cells. Interestingly, the new compound **3** exhibited more sensitivity on cancer cells than normal cells, highlighting its potential as a novel anti-cancer agent. Mechanically, compound **3** treatment increased the ROS generation and triggered apoptosis of human breast cancer cells.

## 1. Introduction

**Figure 1:**
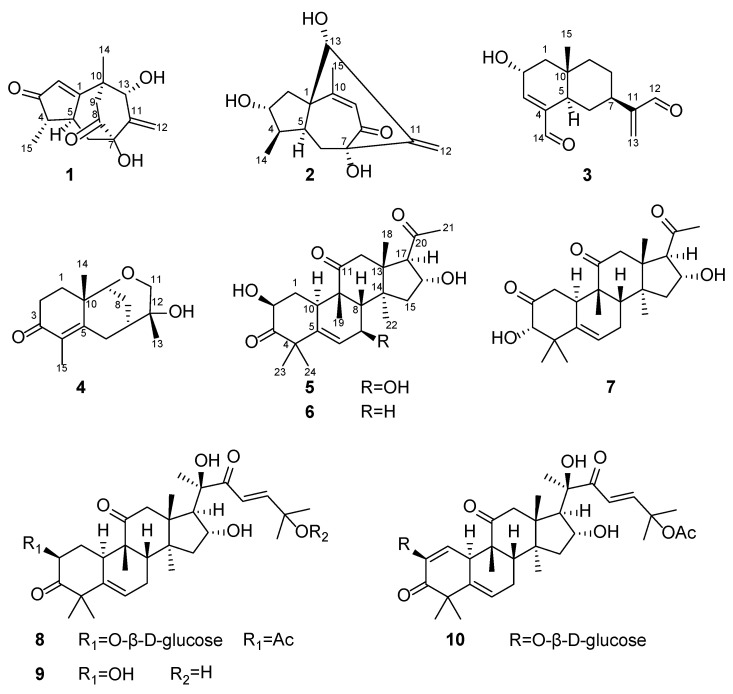
The structures of compounds **1**–**10**.

Cancer is the second leading cause of human death in the worldwide. Despite significant advances in cancer research over the decades, cancer treatment is still facing serious challenges; therefore, it is an important issue to find potential effective anticancer drugs. Chemotherapy is one of the main means of cancer treatment and most of the anti-cancer drugs are obtained by chemical synthesis [1]. Agarwood, the resinous heartwood of *Aquilaria* tree, is secreted by trees as a defense reaction to physical wounding or fungal infection [2,3]. The resins are also known as “the Wood of the Gods”, owing to their widespread use for medicinal, aromatic, and religious purposes in the Middle East, South Asia, Japan, and China [4,5,6]. Agarwood has a long history in traditional therapy and plays an important role, especially in traditional Chinese medicine as an aphrodisiac, sedative, cardiotonic, and carminative, as well as to treat gastric problems, coughs, rheumatism, and high fever [7]. The previous studies of agarwood revealed the presence of sesquiterpenes and 2-(2-phenethyl) chromones [7], sesquiterpene polymers [4], Sesquiterpenoid-Chromone Hetero-hybrids [8] with anti-bacterial, anti-tumor and anti-inflammatory actions, relief of coughs and asthma, anti-depressive and anxiety action, anti-oxidative and anti-aging effects, and effects on the cardiovascular system [9]. For the development of a new generation of potential anticancer drugs, we conducted further studies on agarwood. As a result, structurally and biologically intriguing compounds were characterized by us. In the course of our ongoing chemical investigation of the agarwood of *Aquilaria* *sinensis*, four sesquiterpenoids and six cucurbitacins were identified, with **1**–**5** being new ones (Figure 1).

## 2. Results and Discussion

### 2.1. Structure Elucidation of the Compounds

**Figure 2:**
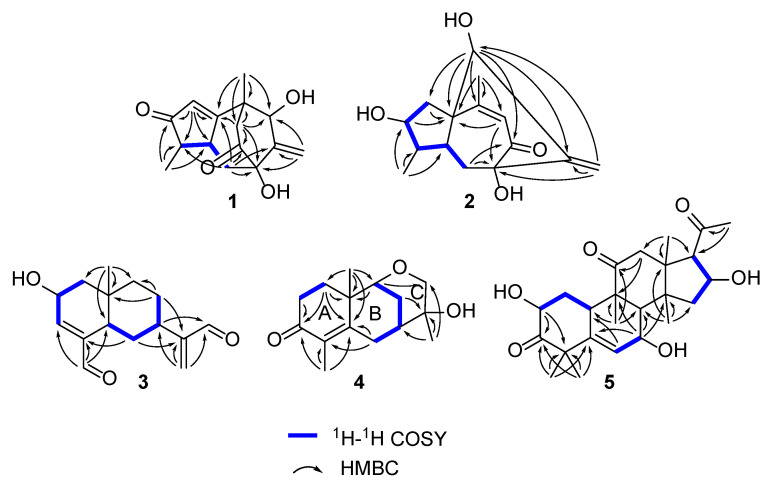
Key ^1^H−^1^H COSY and HMBC correlations for **1**–**5**.

**Figure 3:**
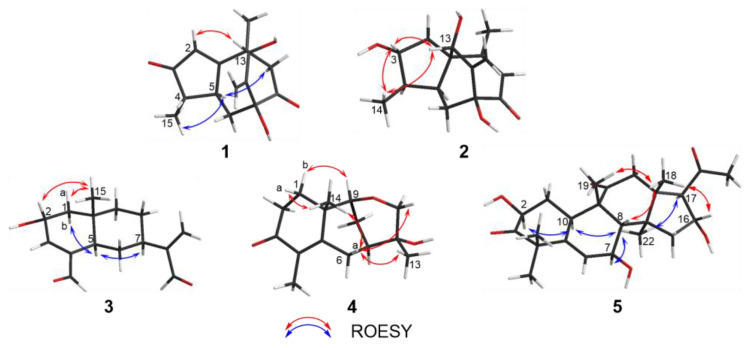
Key ROESY correlations for **1**–**5**.

**Figure 4:**
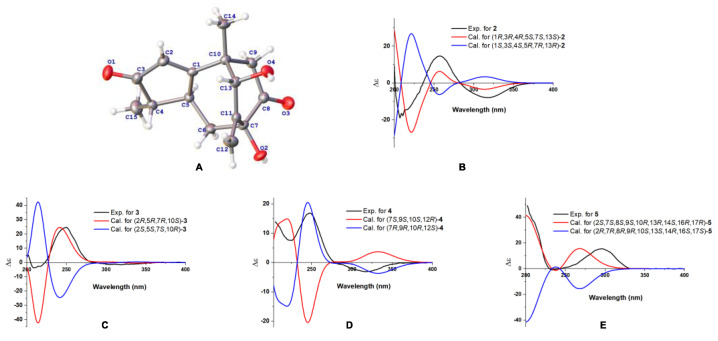
(**A**): X-ray structure of **1**. (**B**–**E**): The calculated and experimental ECD spectra of **2**–**5**.

Compound **1** was obtained as a colorless gum with a negative optical rotation ([α]^20^_D_ −148.3; in MeOH). Its molecular formula was deduced as C_15_H_18_O_4_ on the basis of its HRESIMS (high resolution electrospray ionization mass spectroscopy), ^13^C NMR, and DEPT spectra. The ^1^H NMR spectrum of **1** exhibits two methyl groups (*δ*_H_ 1.40 (s) and 1.14 (d, *J* = 7.44 Hz)). The ^13^C NMR and DEPT spectra (Table 1) indicate 15 carbons ascribed to two methyl groups, three methylenes (including one terminal olefinic methylene), four methines (one olefinic and one aliphatic with oxygenated), and six non-protonated carbons (two olefinic, two ketone, and one aliphatic with oxygenated). The structure of **1** was determined mainly with 2D NMR data. The ^1^H−^1^H COSY spectrum shows H-4/H-5 correlations. The HMBC spectrum shows H_3_-15/C-3 (*δ*_C_ 213.0), C-4, C-5 and H-2/C-1 (*δ*_C_ 188.7), C-3 (*δ*_C_ 213.0), C-4, C-5, allowing us to identify the presence of a five-membered ring and a methyl group at C-4, as shown in Figure 2. These data also indicate that C-2 is connected to C-4 via a ketone carbon. In addition, the ^1^H−^1^H COSY spectrum gives H-5/H-6 correlations and the HMBC spectrum gives H-6/C-1 (*δ*_C_ 187.7), C-7 (*δ*_C_ 81.1), C-11 (*δ*_C_ 154.2), H-12/C-7 (*δ*_C_ 81.1), C-11 (*δ*_C_ 154.2), C-13 (*δ*_C_ 76.7) and H_3_-14/C-1 (*δ*_C_ 187.7), C-13 (*δ*_C_ 76.7), and C-10 correlations, establishing the framework of a seven-membered ring that fuses with the five-membered ring via C-1/C-5, clarifying the positions of methyl group and exocyclic methylene group, and implying C-7 and C-13 are both oxygenated (Figure 2). The HMBC correlations of H_3_-14/C-9, H-9/C-1/C-7/C-8 (*δ*_C_ 211.0)/C-10/C-13, and H-6/C-8 (*δ*_C_ 211.0) indicate that C-10 and C-7 are connected via C-8−C-9 and that C-8 is a ketone carbon (Figure 2). Thus, the planner structure of **1** was determined. There are five chiral centers in the molecule. The ROESY cross peaks of H_3_-15/H-5 indicate H_3_-15 and H-5 are adjacent in space and assigned as *α*-orientation. Further, the nuclear Overhauser effect (NOE) irradiation was used to confirm the other chiral centers. In the ROESY spectrum (Figure 3) of **1**, we found the presence of a seven-membered ring that fuses with the six-membered ring via C-7/C-8/C-9/C-10, which makes the bicyclo[3.2.2]nonane in **1** a rigid structure, in combination with the enhancement of NOE between H-5 and Hb-9 (Figure S7, Supplementary Materials), naturally fixing the relative configurations at C-7 and C-5. In addition, the ROESY interactions of H-2/H-13 indicate that the relative configurations at C-13 should be 13S* (Figure 3). A coplanar arrangement of Hb-9 and H-13 was suggested by a mutual ROESY correlation, while a long-range COSY correlation of Hb-9 and H-13 was explained by a “*W*-path” within a seven-membered ring, also securing the above conclusion. So far, the relative configurations at the chiral carbons of **1** have been assigned. As for the absolute configuration of **1**, a qualified crystal was acquired. The subsequent X-ray diffraction analysis using Cu Kα radiation allows for the assignment of the absolute configuration of **1** as 4*S*,5*S*,7*S*,10*S*,13*S*, with a calculated Flack parameter of 0.02(15) (Figure 4A). In this way, the structure of **1** was identified and named as aquisinenoid A.

Compound **2** was isolated as a colorless gum with a positive optical rotation ([α]^20^_D_ +9.1, in MeOH). The molecular formula of **2** was assigned as C_15_H_20_O_4_, aided with its HRESIMS, ^13^C NMR, and DEPT spectra. The ^1^H NMR spectrum of **2** exhibits two methyl groups (*δ*_H_ 0.96 (d, *J* = 7.34) and 2.08 (d, *J* = 1.41)). The ^13^C NMR and DEPT spectra (Table 1) display 15 carbons classified into two methyl groups, three methylenes (including one terminal olefinic methylene), five methines (one olefinic and two aliphatic with oxygenated), and five non-protonated carbons (two olefinic, one ketone, and one aliphatic with oxygenated). The NMR data of **2** (Table 1) were similar to those of qinan-guaiane-one [10], except for the presence of an additional hydroxy substituted on C-7, which was confirmed by the HMBC correlations of H-12/C-11 (*δ*_C_ 151.5), C-7 (*δ*_C_ 83.3), C-13 (*δ*_C_ 77.0) and H-6/C-7, and C-8 (*δ*_C_ 201.0) (Figure 2). Thus, the planner structure of **2** was determined. The relative configuration of **2** was assigned by ROESY data (Figure 3), which gives a correlation of H-3/H-13/H_3_-14, indicating that these protons are adjacent to each other. In addition, the rigid structure of bicyclo[3.2.2]nonane in **2** clearly indicates the relative configuration at C-5 and C-7 to be 5S*,7S*. Finally, the absolute configuration of **2** was established by the ECD calculations. It was found that the calculated ECD of (1*R*,3*R*,4*R*,5*S*,7*S*,13*S*)-**2** (Figure 4B) is in accordance with that of the experimental one, eventually clarifying the absolute configuration of **2** to be 1*R*,3*R*,4*R*,5*S*,7*S*,13*S* named as aquisinenoid B.

Compound **3** was obtained as a colorless gum with positive optical rotation ([α]^25^_D_ +36.4 in MeOH). The molecular formula of **3** was deduced as C_15_H_20_O_3_ derived from its HRESIMS, ^13^C NMR, and DEPT spectra. The ^1^H NMR spectrum of **3** (Table 2) gives the signal for one methyl (0.93 (s)). The ^13^C NMR and DEPT spectra (Table 2) contain 15 carbon signals classified into one methyl, five methylenes (including one terminal olefinic methylene), six methines (one olefinic, two aldehyde groups, and one aliphatic with oxygenated), and three non-protonated carbons (two olefinic). The 1H and 13C NMR spectra of 3 were very similar to those of 12,15-dioxo-α-selinen [11], except for the presence of an additional hydroxy group substituted on C-2, which was corroborated by the ^1^H−^1^H COSY correlations of H-1/H-2/H-3 (Figure 2). The ROESY correlations of Hb-1, H-7/H-5 revealed that the H-5 and H-7 at C-5 and C-7 were assigned in a *α*-orientation (Figure 3). Meanwhile, the ROESY correlations of Ha-1, H-2/H_3_-15 indicated that H-2 and H_3_-15 are both *β*-oriented. To clarify the absolute configuration of **3**, the ECD calculations were utilized, which shows that the calculated ECD curve of (2*R*,5*R*,7*R*,10*S*)*-***3** (Figure 4C) matches well with the calculated one, evidently indicating the absolute configuration are 2*R*,5*R*,7*R*,10*S*, named as aquisinenoid C.

Compound **4**, obtained as a colorless gum with positive optical rotation ([α]^20^_D_ +29.6 in MeOH), was found to have a molecular formula of C_15_H_22_O_3_ by analysis of its HRESIMS, ^13^C NMR, and DEPT spectra. The ^1^H NMR spectrum of **4** (Table 2) displays three methyl singlets (*δ*_H_ 1.18 (s), 1.78 (s), and 1.16 (s)). The ^13^C NMR and DEPT spectra (Table 2) contain 15 carbon signals ascribed to three methyl groups, five methylenes (one aliphatic with oxygenated), two methines (one aliphatic with oxygenated), and five non-protonated carbons (two olefinic and one ketone). The structure architecture of **4** was mainly assembled with the aid of 2D NMR experiments. The ^1^H−^1^H COSY spectrum of **4** shows correlations of H-1/H-2 (Figure 2). Starting from the three spin systems, the HMBC correlations of H_3_-14/C-1, C-10, C-5 (*δ*_C_ 164.2), H-1/C-3 (*δ*_C_ 201.5), C-10, C-5, and H_3_-15/C-3, C-4 (*δ*_C_ 131.0), C-5, in consideration of the chemical shift of C-3 (*δ*_C_ 201.5), C-4 (*δ*_C_ 131.0), and C-5 (*δ*_C_ 164.2) suggest the presence of a six-membered ring (A), a double bond ∆^(3,4)^, and a carbonyl (*δ*_C_ 164.2, C-5) in **4**. In addition, the ^1^H−^1^H COSY correlations of H-6/H-7/H-8/H-9 and the HMBC correlations of H_3_-14/C-1, C-5 (*δ*_C_ 164.2), C-9 (*δ*_C_ 79.1), C-10 and H-6/C-4 (*δ*_C_ 131.0), C-5, and C-10 imply the presence of another six-membered ring (B) which fuses with ring A via the formation of C-5–C-10 (Figure 2). The additional HMBC H_3_-13/C-7, C-12, C-11 (*δ*_C_ 74.6), H-9/C-11, and H-8/C-12 (*δ*_C_ 68.8), in combination with the chemical shift of C-9 (*δ*_C_ 79.1) and C-12 (*δ*_C_ 68.8), indicates that C-9 and C-11 are connected via an oxygen bridge, and that a methyl and a hydroxy group is attached to C-12. Collectively, the planar structure of **4** was assigned. There are four chiral centers in the molecule. The ROESY correlations of Ha-6/H_3_-14, H-11, and H_3_-13 indicated that these protons are adjacent to each other. The rigid structure of bicyclo[3.2.2]nonane in **4** naturally fixed the relative configurations at C-7 and C-9. To clarify the absolute configuration of **4**, the ECD calculations were utilized, which show that the calculated ECD curve of (7*R*,9*R*,10*R,*12*S*)*-***4** (Figure 4D) matches well with the calculated one, evidently indicating the absolute configuration is 7*R*,9*R*,10*R,*12*S.* As a result, the absolute configuration of **4** was finally confirmed and named aquisinenoid D.

Compound **5**, obtained as a white amorphous powder with positive optical rotation ([α]^20^_D_ +289.7 in MeOH), was found to have a molecular formula of C_24_H_34_O_6_ by analysis of its HRESIMS, ^13^C NMR, and DEPT spectra. Its spectroscopic data were comparable to those of hexanorcucurbitacin D [12], which differs only in the substituent of a hydroxy group at C-7, and were finally assigned to an *β*-OH using the ROESY correlations and ECD method (Figure 4E). Therefore, the structure of compound **5** was named aquisinenoid E.

The five known compounds were identified as hexanorcucurbitacin D (**6**) [12], hexanorisocucurbitacin D (**7**) [12], arvenin I (**8**) [13], cucurbitacin D (**9**) [14], 2-O-*β*-D-glucopyranosylcucurbitacin I (**10**) [15], by a comparison of their spectroscopic data with those reported in the literature. 

### 2.2. Biological Evaluation

To explore the potential biological functions of these compounds, the MTT assay was used to evaluate the cytotoxic effect of the compounds on the human cancer cell lines, including human colon cancer cell line (HT29), human cervical cancer cell line (HeLa), human liver cancer cell line (HepG2), human breast cancer cell line (MCF-7), human lung adenocarcinoma cell line A549, and human normal liver cell line LO2. The results in Figure S37 (Supplementary Materials) show that compound **9** and the new compound **3** at 5μM inhibited the proliferation and survival of most of the cancer cells, especially in the human breast cancer cell line (MCF-7). Interestingly, compared with the positive control cisplatin (DDP), which is one of the most potent and widely used drugs for the treatment of various solid cancers [16], compound **3** was more sensitive to human breast cancer and exhibited significantly less sensitivity on the normal liver cells LO2.

**Figure 5:**
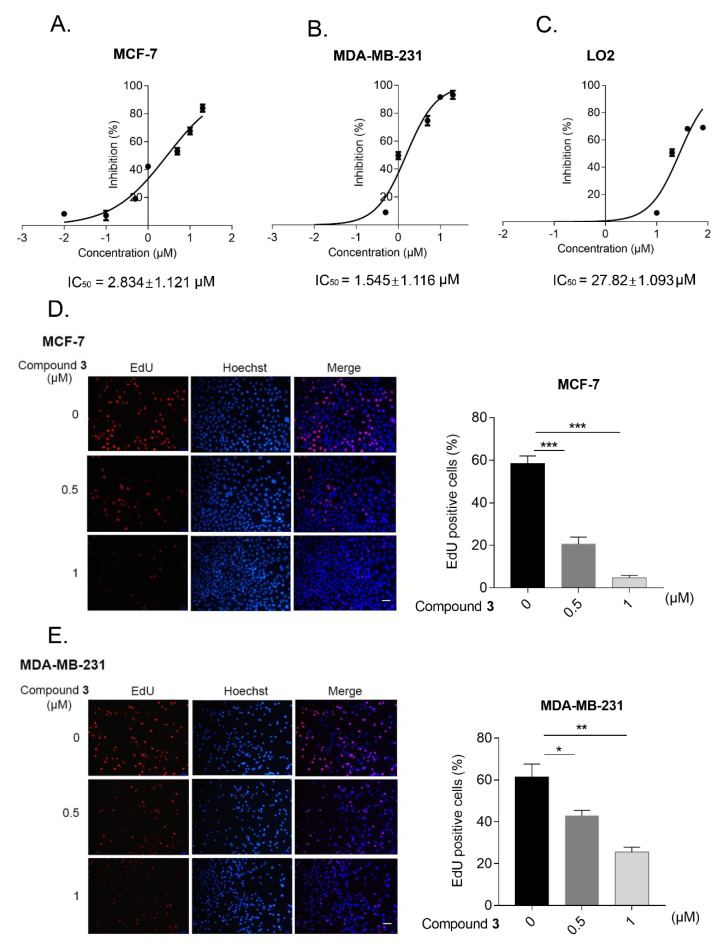
Compound 3 inhibited the proliferation and growth of human breast cancer cells. (**A**–**C**); MCF-7 (**A**), MDA-MB-231 (**B**), and LO2 cells (**C**) were treated with indicated concentration of compound **3** for 24 h, MTT assay was used to detect cell viability. GraphPad Prism 7 was used to draw the picture and SPSS was utilized to analyze IC50 of compound **3**; (**D**,**E**), EdU assay was applied to detect the cell proliferation ability after treatment with compound **3** in MCF-7 (**D**) and MDA-MB-231 cells (**E**). Fluorescence microscopy was performed to capture the picture and Image J software was used to analyze the EdU positive ratio, scale bar, 40 μm. * p < 0.05, ** p < 0.01, *** p < 0.001.

To further evaluate the anti-cancer activity of compound **3** in human breast cancer cells, apart from MCF-7, we used another breast cancer cell line, MDA-MB-231. After treatment with the indicated concentration of compound **3** for 24 h, the half maximal inhibitory concentration (IC_50_) of this new compound in the MCF-7 cells (IC_50_ = 2.834 ± 1.121 μM), MDA-MB-231 cells (IC_50_ = 1.545 ± 1.116 μM) and normal liver cells LO2 (IC_50_ = 27.82 ± 1.093 μM) were measured by MTT assay (Figure 5A–C). In addition, the EdU assay implied that the cell proliferation and cell growth decreased after treatment with the different concentrations of compound **3** in human breast cancer cells (Figure 5D,E).

Recently, the accumulating evidence suggests that the reactive oxygen species (ROS), which include hydrogen peroxide (H_2_O_2_), superoxide anion radical (O_2_^−^) and hydroxyl radical (OH^−^) [17], are the byproducts of oxygen metabolism and played an important role in cancer development and are associated with cell proliferation and cell growth [18]. To further determine the inhibitory effect of compound **3** on human breast cancer cells, we first examined the ROS production by flow cytometry in MCF-7, MDA-MB-231, and LO2 cells after compound **3** treatment. H_2_O_2_ served as a positive control. Compared with the control group (0 μM group), compound **3** remarkably triggered an elevation of the ROS level (Figure S38A,B, Supplementary Materials) in both of the MCF-7 and MDA-MB-231 breast cancer cells, however, it had no effect on the normal liver cells LO2 (Figure S38C, Supplementary Materials).

**Figure 6:**
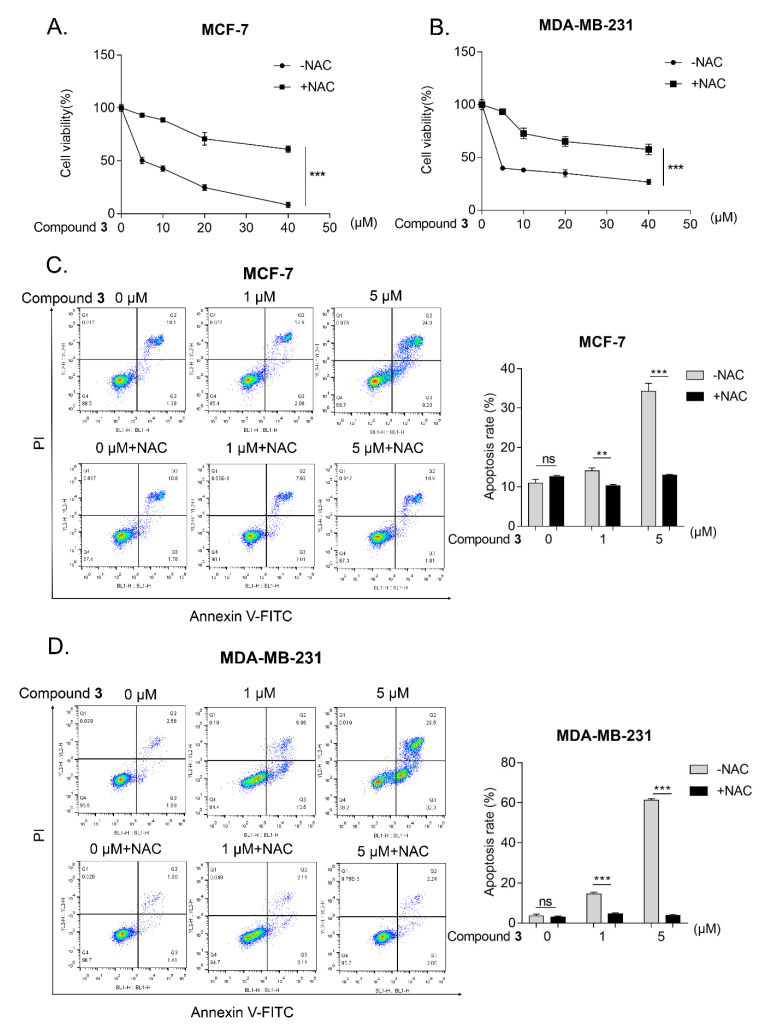
ROS are involved in compound 3-induced cancer cells apoptosis. (**A**,**B**), MCF-7 (**A**) and MDA-MB-231 cells (**B**) were pre-treated with NAC (1 mM) for 1 h and then treated with different concentration of compound **3** for 24 h, MTT assay was used to examine the cell viability; (**C**,**D**), MCF-7 (**C**) and MDA-MB-231 cells (**D**) were exposed to the indicated concentration of compound **3** or co-treated with 1 mM NAC for 24 h and the cell apoptosis was detected by flow cytometry and bar chart indicated that the apoptosis rate via FlowJo software. ** *p* < 0.01, *** *p* < 0.001; ns represents nonsignificant effects.

*N*-acetyl-L-cysteine (NAC) is a commonly-used ROS scavenger [19]. After treatment NAC, the inhibition of compound **3** on cell viability was blocked significantly in the MCF-7 and MDA-MB-231 cells (Figure 6A,B). Apoptosis, a kind of programmed cell death, is involved in various diseases, especially in human cancer [20]. ROS as one of the stress agents is a well-characterized apoptosis trigger. In addition, several reports have shown that ROS is a key molecule in cell apoptosis [21]. To assess whether the compound **3**-induced ROS production is related to apoptosis, flow cytometry was performed to detect the pro-apoptotic effect of compound **3** on the MCF-7 and MDA-MB-231 cells. Because NAC is commonly used to block ROS generation [22], the cells were co-treated with NAC and compound **3** for 24 h. The results showed that compound **3** enhanced the occurrence of apoptosis in a dose-dependent manner and meanwhile, the apoptosis is largely accounted for by the ROS generation (Figure 6C,D). 

Taken together, most of the cancer cells, especially in the human breast cancer cells, exhibit exquisite sensitivity to the compound **3** over the normal cells. Compound **3**, a new natural compound, may act as a potential anti-cancer agent by increasing the ROS production and triggering apoptosis of human breast cancer cells.

## 3. Experimental Section

### 3.1. General Procedures

The UV spectra were obtained on a Shimadzu UV-2600 spectrometer (Shimadzu Corporation, Tokyo, Japan). The CD spectra were measured on a Chirascan instrument (Agilent Technologies, Santa Clara, CA, USA). The NMR spectra were recorded on a Bruker AV-600 spectrometer, with TMS as an internal standard. The HRESIMS were collected by a SCIEX X500R QTOF MS spectrometer. The column chromatography was performed by using silica gel (200–300 mesh; Qingdao Marine Chemical Inc., Shandong, China), RP-18 silica gel (40–60 μm; Daiso Co., Amagasaki, Japan), MCI gel CHP 20P (75–150 μm; Mitsubishi Chemical Industries, Tokyo, Japan), and Sephadex LH-20 (Amersham Pharmacia, Uppsala, Sweden). The optical rotations were measured on an Anton Paar MCP-100 digital polarimeter, (ANTON PAAR, Shanghai, China). The semi-preparative or analytic HPLC was carried out using an Agilent 1200 liquid chromatograph (Agilent Technologies, Santa Clara, CA, USA). The column used was a YMC-Pack ODS-A 250 × 9.4 mm, i.d., 5 µm (YMC Co., Ltd., Kyoto, Japan.).

### 3.2. Plant Material

The resinous wood of A. sinensis was purchased from Hainan Xiangshu Agarwood Industry Ground Co., Ltd. Hainan, China, July 2018. A voucher specimen (CHYX0642), identified by the Gansu Institute for Drug Control, is deposited at the School of Pharmaceutical Sciences, Shenzhen University, ShenZhen, China.

### 3.3. Extraction and Isolation

The dried and powdered agarwood sample (15 kg) was extracted by percolating with 95% EtOH (150 L, flow rate: 4.5 L/h) to afford a crude extract (1.7 kg), which was suspended in water, followed by partition with EtOAc to afford an EtOAc-soluble extract (1.3 kg). The EtOAc extract was separated by a MCI gel CHP 20P column, eluted with gradient aqueous MeOH (50–100%) to provide nine portions (Fr.1–Fr.9). The Fr.1 (15.1 g) was separated via a YMC GEL ODS-A-HG column, eluted with aqueous MeOH (40–100%), to provide four portions (Fr.1.1–Fr.1.4). Among them, Fr.1.1 (8.7 g) was subjected to a Sephadex LH-20 (MeOH) to provide three portions (Fr.1.1.1–Fr.1.1.3). The Fr.1.1.2 (7.0 g) was further separated via MCI gel CHP 20P, washed with aqueous MeOH (35–100%), to provide four portions (Fr.1.1.2.1–Fr.1.1.2.4). The Fr.1.1.2.1 (4.7 g) was further separated via vacuum liquid chromatography and washed with CH_2_Cl_2_–MeOH (100:1–10:1) to provide twelve portions (Fr.1.1.2.1.1–Fr.1.1.2.1.12). Of them, the Fr.1.1.2.1.3 (671.0 mg) was subjected to preparative HPLC with aqueous MeOH (5–20%) to give Fr.1.1.2.1.3.1–Fr.1.1.2.1.3.5. The Fr.1.1.2.1.3.4 (127.5 mg) was further purified by semi-preparative HPLC on YMC-PACK-ODS-A with aqueous MeCN (10%) to afford **2** (8.1 mg, *t*_R_ = 15.7 min; flow rate: 3 mL/min) and **1** (5.1 mg, *t*_R_ = 29.0 min; flow rate: 3 mL/min). The Fr.1.1.2.4 (1.3 g) was further divided into nine parts (Fr.1.1.2.4.1–Fr.1.1.2.4.9) by a vacuum liquid chromatography eluted with CH_2_Cl_2_–acetone (100:1–1:1). The Fr.1.1.2.4.7 (22.6 mg) was further purified by semi-preparative HPLC on YMC-PACK-ODS-A with aqueous MeCN (13%) to afford **5** (6.2 mg, *t*_R_ = 30.7 min; flow rate: 3 mL/min). The Fr.1.3 (2.0 g) was divided into ten parts (Fr.1.3.1–Fr.1.3.10) by a vacuum liquid chromatography eluted with CH_2_Cl_2_–MeOH (100:1–10:1). Of them, Fr.1.3.7 (178.8 mg) was subjected to preparative TLC to give Fr.1.3.7.1–Fr.1.3.7.7. The Fr.1.3.7.1 (76.9 mg) was separated by semi-preparative HPLC with aqueous MeOH (28%) to afford **4** (11.3 mg, *t*_R_ = 36.3 min, flow rate: 3 mL/min). The Fr.4 (81.9 g) was subjected to a RP-18 column eluted with aqueous MeOH (40–100%) to yield nine fractions (Fr.4.1–Fr.4.9). Of which, the Fr.4.8 (5.5 g) was further divided into nine parts (Fr.4.8.1–Fr.4.8.9) by a vacuum liquid chromatography eluted with petroleum ether–EtOAC (100:1–1:1). Of them, the Fr.4.8.8 (324.7 mg) was subjected to preparative HPLC with aqueous MeOH to give Fr.4.8.4.1–Fr.4.8.4.4. Fr.4.8.4.4 (52.0 mg) was further purified by semi-preparative HPLC on YMC-PACK-ODS-A with aqueous MeCN (10%) to afford **9** (16.4 mg, *t*_R_ = 29.2 min; flow rate: 3 mL/min). Fr.4.8.8 (324.7 mg) was passed through Sephadex LH-20 (MeOH) to yield three fractions Fr.4.8.8.1–Fr.4.8.8.3. Fr.4.8.8.1 (74.0 mg) was further purified by semi-preparative HPLC on YMC-PACK-ODS-A with aqueous MeCN (30%) to afford **8** (10.5 mg, *t*_R_ = 17.3 min; flow rate: 3 mL/min) and with aqueous MeCN (35%) to afford **10** (14.3 mg, *t*_R_ = 17.3 min; flow rate: 3 mL/min). The Fr.4.6 (30.0 g) was further separated via a silica gel column washed with petroleum CH_2_Cl_2_–acetone (100:1–1:1) to provide thirteen portions (Fr.4.6.1–Fr.4.6.13). The Fr.4.6.1 (1.7 g) was passed through Sephadex LH-20 (MeOH) to yield three fractions Fr.4.6.1.1–Fr.4.6.1.3. The Fr.4.6.1.3 (94.3 mg) was further purified by semi-preparative HPLC on Agilent with aqueous MeCN (45%) to afford **3** (3.1 mg, *t*_R_ = 26.3 min; flow rate: 3 mL/min). The Fr.4.6.6 (494.9 mg) was passed through Sephadex LH-20 (MeOH) to yield five fractions Fr.4.6.6.1–Fr.4.6.6.5. The Fr.4.6.6.1 (146.1 mg) was further purified by semi-preparative HPLC on YMC-PACK-ODS-A with aqueous MeOH (58%) to afford **7** (3.51 mg, *t*_R_ = 20.7 min; flow rate: 3 mL/min). The Fr.4.6.6.4 (47.7 mg) was further purified by semi-preparative HPLC on Agilent with aqueous MeCN (28%) to afford **6** (8.1 mg, *t*_R_ = 23.7 min; flow rate: 3 mL/min).

### 3.4. Compound Characterization Data

Compound **1**: colorless gum. [α]^20^_D_ −148.3 (*c* 0.03, MeOH); CD (MeOH), ∆*ε*_2__21_ −4.71, ∆*ε*_248_ +1.73; UV (MeOH) *λ*_max_ (log *ε*) 211 (3.46) nm, 232 (3.69) nm; HRESIMS: *m*/*z* 263.1280 [M + H]^+^ (calcd. for C_15_H_18_O_4_, 263.1278); ^1^H and ^13^C NMR data, see Table 1.

Compound **2**: colorless gum. [α]^20^_D_ +9.1 (*c* 0.02, MeOH); CD (MeOH), ∆*ε*_2__10_ −4.61, ∆*ε*_257_ +3.91, ∆*ε*_316_ −2.11, UV (MeOH) *λ*_max_ (log *ε*) 216 (3.37) nm, 247 (3.82) nm; ESIMS: *m*/*z* 265.1442 [M + H]^+^ (calcd. for C_15_H_20_O_4_, 265.1430); ^1^H and ^13^C NMR data, see Table 1.

Compound **3**: colorless gum. [α]^25^_D_ +36.4 (*c* 0.011, MeOH); CD (MeOH), ∆*ε*_211_ −0.94, ∆*ε*_248_ +6.18; UV (MeOH) *λ*_max_ (log *ε*) 211 (3.21) nm, 246 (3.99) nm; HRESIMS: *m*/*z* 249.1483 [M + H]^+^ (calcd. for C_15_H_20_O_3_, 249.1485); ^1^H and ^13^C NMR data, see Table 2.

Compound **4**: colorless gum. [α]^20^_D_ +29.6 (*c* 0.027, MeOH); CD (MeOH), *∆ε*_222_ +1.83, *∆ε*_2__47_ +4.38, Δ*ε*_3__11_ −0.81; UV (MeOH) *λ*_max_ (log *ε*) 211 (3.12) nm, 249 (3.87) nm; HRESIMS: *m*/*z* 251.1637 [M + H]^+^ (calcd. for C_15_H_22_O_3_, 251.1642); ^1^H and ^13^C NMR data, see Table 2.

Compound **5**: white powder. [α]^20^_D_ +289.7 (*c* 0.068, MeOH); CD (MeOH), Δ*ε*_230_ −0.27, Δ*ε*_295_ +6.53; UV (MeOH) *λ*_max_ (log *ε*) 200 (4.02) nm; HRESIMS: *m*/*z* 441.2255 [M + H]^+^ (calcd. for C_24_H_34_O_6_, 441.2250); ^1^H and ^13^C NMR data, see Table 3.

### 3.5. ECD Calculation for Compounds ***1**–**5***

The conformation search using molecular mechanics calculations was performed in CONFLEX version 7.0 with MMFF force field with an energy window for acceptable conformers (ewindow) of 5 kcal/mol above the ground state, a maximum number of conformations per molecule (maxconfs) of 100, and an RMSD cutoff (rmsd) of 0.5Å. Then, the predominant conformers were optimized at B3LYP/6-311 + g (d,p) level in Gaussian 09 [23]. The optimized conformation geometries and thermodynamic parameters of all of the selected conformations were provided. The optimized conformers of **1**–**5** were used for the ECD calculation, which were performed with Gaussian 09 B3LYP/6-311 + g (d,p)) [24]. The solvent effects were taken into account by the polarizable-conductor calculation model (PCM, methanol as the solvent). The percentages for each conformation are shown in Figures S36–S40 (Supplementary Materials).

### 3.6. Bioactivity Assay

#### 3.6.1. Cell Culture

The human normal liver cell line (LO2), liver cancer cell line (HepG2), colon cancer cell line (HT29), cervical carcinoma cell line (HeLa), breast cancer cell line (MCF-7) and (MDA-MB-231), and lung adenocarcinoma cell line (A549) were purchased from American Tissue Culture Collection (Manassas, VA, USA). The MCF-7 and A549 cells were cultured in RPMI 1640 medium (Wisent, Nanjing, China) containing 10% fetal bovine serum (ExCell, Taicang, China) and penicillin (100 U/mL)/streptomycin (100 μg/mL) (Wisent, Canada). The HepG2, HeLa, and HT29 cells were cultured in DMEM medium supplemented with 10% FBS and penicillin (100 U/mL)/streptomycin (100 μg/mL). All types of cells were kept at 37 °C in a humidified atmosphere of 95% air and 5% CO_2_.

#### 3.6.2. Cell Viability Assay

The cell viability was measured by MTT assay. The cells were seeded in 96-well plates at 5 × 10^3^ cells per well and incubated overnight. After treatment, the various concentrations of compounds for 24 h, 20 μL MTT solution (5 mg/mL, Solarbio, Beijing, China) were added into each well, then incubated at 37 °C for 4 h, and the medium was discarded. Subsequently, the formed formazan crystals were dissolved in 150 μL DMSO (Aladdin, Shanghai, China). The absorbance values for each well were measured at OD490 nm by Microplate Reader (Thermo, Waltham, MA, USA). All of the experiments were performed in triplicate and the data were presented as mean ± SD.

#### 3.6.3. EdU Assay

The cells were seeded into 12-well plates; after incubation with 1 mL medium containing 10 μM EdU at 37 °C with 5% CO_2_ for 2 h, the cells were fixed with 4% paraformaldehyde for 30 min at room temperature. Then, the cells were permeabilized with 0.3% Triton X-100 in PBS for 15 min. Edu solution was added into cells, according to the manufacturer’s instructions (Beyotime, Shanghai, China), and the nuclei were stained with Hoechst 33342. Then, the images were captured by a fluorescence microscope.

#### 3.6.4. Flow Cytometry

The effect of the compounds on the apoptosis of MCF-7 and MDA-MB-231 was evaluated by flow cytometry assay with Annexin V-FITC/PI kit (Yeasen, Shanghai, China). Briefly, the harvested cells were resuspended in 100 μL binding buffer and incubated with 5 μL Annexin V-FITC and 10 μL PI in darkness at room temperature for 10~15 min. Subsequently, 400 µL of binding buffer was added before being measured on Attune™ Nxt acoustic focusing flow cytometer system (Thermo Fisher Scientific, Waltham, MA, USA). The FlowJo VX10 software (Becton, Dickinson & Company, Franklin Lakes, NJ, USA) was used to analyze the data.

#### 3.6.5. Measurement of Reactive Oxygen Species (ROS)

To measure the intracellular ROS generation, according to the manufacturer’s instructions (Beyotime, Shanghai, China), the cells were seeded in 6-well plates; after treatment with a different concentration of compound for 24 h, the cells were harvested and loaded with DCFH-DA (10 μM) probe. After incubation at 37 °C for 30 min, the cells were washed with serum-free medium three times and the fluorescence intensity was measured by flow cytometry. The data were analyzed by FlowJo software; the experiments were repeated three times.

#### 3.6.6. Statistical Analysis

The data were presented as mean ± SD. The difference between the two groups was performed by a two-tailed student’s *t*-test. For multiple comparisons, one-way analysis of variance (ANOVA) was applied and the graphs were created by GraphPad Prism software (Version 7). The difference was considered as statistically significant when *p* < 0.05.

## 4. Conclusions

Agarwood, also known as Chen Xiang in China, is a type of highly valuable aromatic resinous heartwood [25]. It has a long history of medicinal use in Chinese medicine [7]. In this study, we obtained two new guaiane sesquiterpenes, two new eudesmane-type sesquiterpenoids, one new cucurbitacin, and five known cucurbitacins from agarwood of *Aquilaria sinensis*. The compounds **3** and **9** have significant anti-cancer effects on most human cancer cells, especially the human breast cancer cells. The lower sensitivity of new compound **3** to normal cells indicated that it might be a potential anti-cancer agent against breast carcinoma. Further study revealed that compound **3** exerted its anti-cancer activity via modulating the ROS-induced apoptosis. In addition, we also observed new compound **2** could selectively inhibit the cell viability of lung cancer cells, but had less effect on the other types of cancer cells or normal cells, indicating its potential use in lung cancer treatment; obviously, further study is needed to confirm this presumption.

CCDC 2195955 contains the supplementary crystallographic data for this paper. These data can be obtained free of charge via www.ccdc.cam.ac.uk/data_request/cif (accessed on 10 August 2022), or by emailing data_request@ccdc.cam.ac.uk, or by contacting The Cambridge Crystallographic Data Centre, 12 Union Road, Cambridge CB2 1EZ, UK; Fax: +44-1223-336033.

## Figures and Tables

**Table 1:** ^1^H (600 MHz) and ^13^C (150 MHz) NMR data of **1** and **2** in MeOD.

	1			2	
no.	*δ*_H_	*δ*_C_	no.	*δ*_H_	*δ*_C_
1		187.7, C	1		55.6, C
2	6.12 (d, 1.72)	129.5, CH	2	Ha: 2.45 (dd, 14.02, 6.46)	39.6, CH_2_
				Hb: 2.05 (dd, 14.02, 9.23)	
3		213.0, C	3	4.20 (dt, 9.23, 6.46)	74.9, CH
4	2.00 (qd, 7.44, 2.05)	51.2, CH	4	2.23 (m)	41.3, CH
5	2.83 (ddt, 11.04, 9.03, 2.05)	47.4, CH	5	2.16 (dt, 12.27, 8.89)	49.0, CH
6	Ha: 2.42 (dd, 12.27, 9.03)	49.9, CH_2_	6	Ha: 1.97 (t, 12.27)	32.0, CH_2_
	Hb: 1.63 (dd, 12.27, 11.04)			Hb: 1.57 (dd, 12.27, 8.89)	
7		81.1, C	7		83.3, C
8		211.0, C	8		201.0, C
9	Ha: 2.72 (d, 19.40)	44.0, CH_2_	9	6.06 (d, 1.41)	128.3, CH
	Hb: 2.66 (dd, 19.40, 0.72)				
10		43.4, C	10		173.4, C
11		154.2, C	11		151.5, C
12	Ha: 5.59 (br s)	116.8, CH_2_	12	Ha: 5.45 (dd, 2.64, 1.41)	111.2, CH_2_
	Hb: 5.30 (br s)			Hb: 5.32 (t, 1.41)	
13	4.09 (br s)	76.7, CH	13	4.65 (t, 2.64)	77.0, CH
14	1.40 (s)	23.7, CH_3_	14	0.96 (d, 7.34)	9.7, CH_3_
15	1.14 (d, 7.44)	15.5, CH_3_	15	2.08 (d, 1.41)	26.4, CH_3_

**Table 2:** ^1^H (600 MHz) and ^13^C (150 MHz) NMR data of **3** and **4** in MeOD.

	3			4	
no.	*δ*_H_	*δ*_C_	no.	*δ*_H_	*δ*_C_
1	Ha: 1.88 (ddd, 12.36, 6.60, 1.42)	47.4, CH_2_	1	Ha: 2.11 (dt, 13.52, 4.65)	34.6, CH_2_
	Hb: 1.33 (m)			Hb: 1.78 (overlap)	
2	4.55 (m)	66.6, CH	2	Ha: 2.47 (ddd, 16.73, 13.90, 5.10)	34.3, CH_2_
				Hb: 2.36 (dt, 16.73, 4.65)	
3	6.64 (td, 3.12, 1.42)	154.1, CH	3		201.5, C
4		143.2, C	4		131.0, C
5	2.34 (dq, 12.76, 3.12)	45.1, CH	5		164.2, C
6	Ha: 2.67 (dt, 12.76, 3.12)	27.5, CH_2_	6	Ha: 2.78 (dd, 12.92, 2.53)	29.3, CH_2_
	Hb: 1.19 (q-like, 12.76)			Hb: 2.01 (t-like, 12.92)	
7	2.58 (m)	38.5, CH	7	1.66 (m)	42.1, CH
8	1.61 (m)	27.2, CH_2_	8	Ha: 1.92 (m)	31.1, CH_2_
				Hb: 1.67 (m)	
9	Ha: 1.55 (m)	40.3, CH_2_	9	3.36 (dd, 10.91, 4.27)	79.1, CH
	Hb: 1.45 (td, 12.92, 4.87)				
10		36.0, C	10		43.0, C
11		156.3, C	11		74.6, C
12	9.51 (s)	196.2, CH	12	Ha: 3.51 (d, 10.80)	68.8, CH_2_
				Hb: 3.47 (d, 10.80)	
13	Ha: 6.36 (br s)	134.6, CH_2_	13	1.18 (s)	21.7, CH_3_
	Hb: 6.08 (br s)				
14	9.46 (s)	196.7, CH	14	1.16 (s)	16.3, CH_3_
15	0.93 (s)	16.7, CH_3_	15	1.78 (s)	11.3, CH_3_

**Table 3:** ^1^H (600 MHz) and ^13^C (150 MHz) NMR data of **5** in MeOD.

5					
no.	*δ*_H_	*δ*_C_	no.	*δ*_H_	*δ*_C_
1	Ha: 2.12 (ddd, 12.52, 5.76, 3.74)	36.5, CH_2_	13		50.4, C
	Hb: 1.26 (q, 12.52)		14		48.8, C
2	4.60 (dd, 12.81, 5.76)	72.7, CH	15	Ha: 2.17 (m)	46.0, CH_2_
3		213.3, C		Hb: 1.58 (br d, 13.42)	
4		51.8, C	16	4.86 (overlap)	72.5, CH
5		146.1, C			
6		122.7, CH	17	3.20 (d, 6.76)	67.8, CH
7	4.12 (dd, 5.34, 1.07)	66.9, CH	18	0.70 (s)	20.2, CH_3_
8	2.08 (br s)	53.5, CH	19	1.16 (s)	21.4, CH_3_
9		49.4, C	20		210.6, C
10	3.04 (ddt, 12.96, 3.74, 1.98)	35.6, CH	21	2.20 (s)	31.8, CH_3_
11		213.9, C	22	1.32 (s)	19.8, CH_3_
12	Ha: 2.45 (d, 14.72)	48.2, CH_2_	23	1.36 (s)	21.9, CH_3_
	Hb: 3.46 (d, 14.72)		24	1.33 (s)	30.3, CH_3_

## Data Availability

All the data in this research are presented in manuscript and supplementary material.

## Supplementary Materials

The following supporting information can be downloaded at: https://www.mdpi.com/article/10.3390/molecules27165350/s1, Figures S1–S7: NMR spectra of **1**; Figure S8: HREIMS of **1**; Figures S9–S14: NMR spectra of **2**; Figure S15: HRESIMS of **2**; Figures S16–S21: NMR spectra of **3**; Figure S22: HREIMS of **3**; Figures S23–S28: NMR spectra of **4**; Figure S29: HRESIMS of **4**; Figures S30–S35: NMR spectra of **5**; Figure S36: HRESIMS of **5**; Figure S37: Effects of Compound **1**–**10** and DDP on cell viability in human cancer cells; Figure S38: Compound **3** promoted the production of reactive oxygen species in human breast cancer cells; Figures S39–S43: The lowest energy conformers of **1**–**5** (the relative populations are in parentheses); Table S1: Crystal data and structure refinement for **1**; Table S2: Fractional Atomic Coordinates (×10^4^) and Equivalent Isotropic Displacement Parameters (Å^2^ × 10^3^) for **1**. Ueq is defined as 1/3 of the trace of the orthogonalized UIJ tensor; Table S3: Anisotropic Displacement Parameters (Å^2^ × 10^3^) for **1**. The Anisotropic displacement factor exponent takes the form: −2π2[h2a*2U11 + 2hka*b*U12 + …]; Table S4: Bond Lengths for **1**; Table S5: Bond Angles for **1**; Table S6: Torsion Angles for **1**; Table S7: Hydrogen Atom Coordinates (Å × 10^4^) and Isotropic Displacement Parameters (Å^2^ × 10^3^) for **1**; Table S8: Extracted heats and weighting factors of the optimized conformers of **2**–**5**; Table S9: The Cartesian coordinates of the lowest energy conformers for **2**–**5**. Ref. [24] is cited in the Supplementary Materials.

## Sample Availability

Samples of the compounds **1**–**10** are available from the authors.
