# Supplementary material for: Anti-Cancer Effect of Sesquiterpene and Triterpenoids from Agarwood of Aquilaria sinensis
Source: Molecules. 2022 Aug 22;27(16):5350. doi: 10.3390/molecules27165350 (PMC9413513; doi:10.3390/molecules27165350)
Supplement: Supplementary file 1 [file molecules-27-05350-s001.zip › molecules-1848918-SI.pdf]

Supplementary Materials

Anti-Cancer Effect of Sesquiterpene and Triterpenoids from Agarwood of *Aquilaria sinensis*

Lili Chen [1,†], Yunyun Liu [2,†], Yifei Li [2,†], Wu Yin [1,*] and Yongxian Cheng [2,*]

[1] State Key Lab of Pharmaceutical Biotechnology, College of Life Sciences, Nanjing University, Nanjing 210023, China
[2] Health Science Center, Institute for Inheritance-Based Innovation of Chinese Medicine, School of Pharmaceutical Sciences, Shenzhen University, Shenzhen 518060, China
* Correspondence: wyin@nju.edu.cn (W.Y.); yxcheng@szu.edu.cn (Y.C.); Tel.: +86-0755-2690-2073 (Y.C.)
† These authors contributed equally to this work.

Table of Contents

1. **Figures S1–S8 NMR spectra and HRESIMS of 1**
2. **Figures S9–S15 NMR spectra and HRESIMS of 2**
3. **Figures S16–S22 NMR spectra and HRESIMS of 3**
4. **Figures S23–S29 NMR spectra and HRESIMS of 4**
5. **Figures S30–S36 NMR spectra and HRESIMS of 5**
6. **Table S1 Crystal data and structure refinement for 1.**
7. **Table S2 Fractional Atomic Coordinates ($\times10^4$) and Equivalent Isotropic Displacement Parameters (Å$^2\times10^3$) for 1. U_{eq} is defined as 1/3 of the trace of the orthogonalised U_{IJ} tensor.**
8. **Table S3 Anisotropic Displacement Parameters (Å$^2\times10^3$) for 1. The Anisotropic displacement factor exponent takes the form: $-2\pi^2[h^2a^{*2}U_{11}+2hka^*b^*U_{12}+\ldots]$.**
9. **Table S4 Bond Lengths for 1.**
10. **Table S5 Bond Angles for 1.**
11. **Table S6 Torsion Angles for 1.**
12. **Table S7 Hydrogen Atom Coordinates (Å$\times10^4$) and Isotropic Displacement Parameters (Å$^2\times10^3$) for 1.**
13. **Figure S37. Effects of Compound 1-10 and DDP on cell viability in human cancer cells.**
14. **Figure S38. Compound 3 promoted the production of reactive oxygen species in human breast cancer cells.**
15. **Figure S39. The lowest energy conformers of 1**
16. **Figure S40. The lowest energy conformers of 2**
17. **Figure S41. The lowest energy conformers of 3**
18. **Figure S42. The lowest energy conformers of 4**
19. **Figure S43. The lowest energy conformers of 5**
20. **Table S8. Extracted heats and weighting factors of the optimized conformers of 2−5 at B3LYP/6-311g(d,p) level**
21. **Table S9. The Cartesian coordinates of the lowest energy conformers for 2−5**

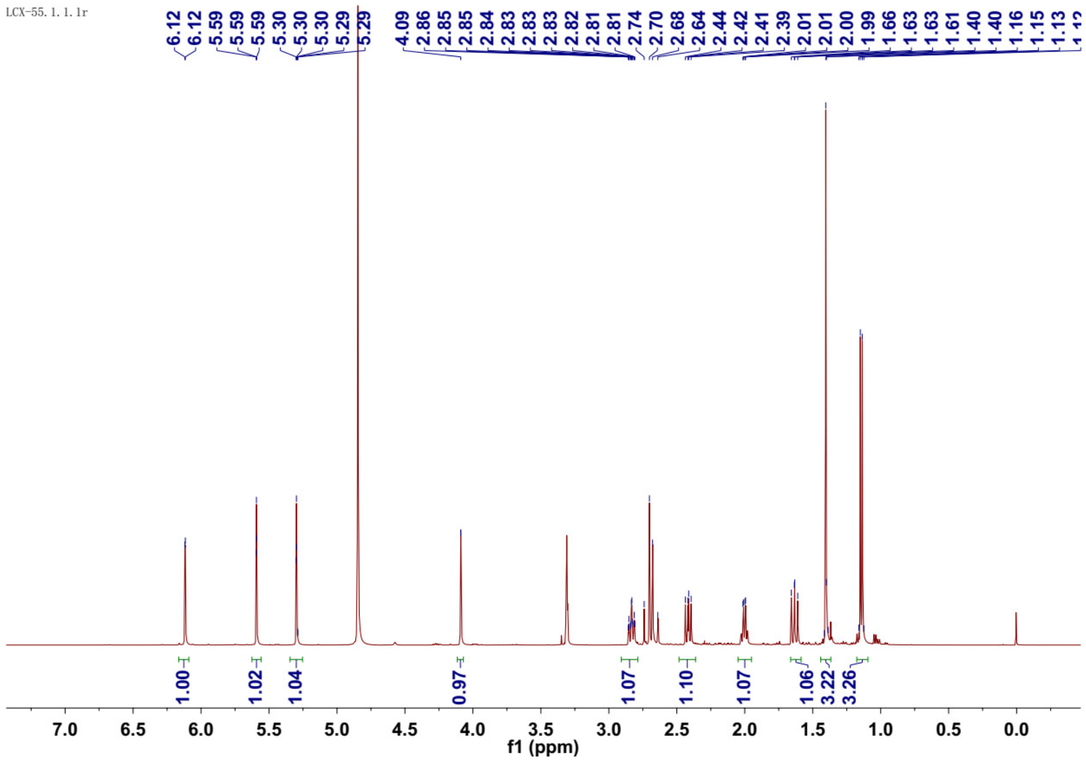

Figure S1. ^{1}H NMR spectrum of **1** in Methanol-d_4.

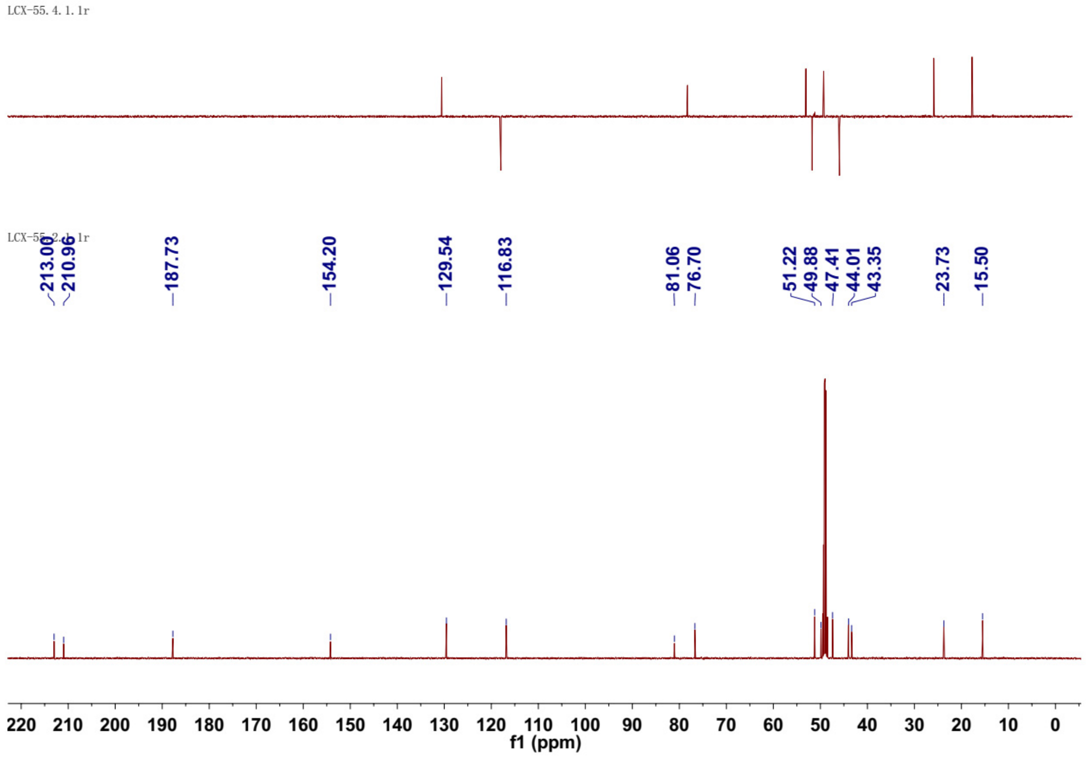

Figure S2. ^{13}C NMR and DEPT spectra of **1** in Methanol-d_4.

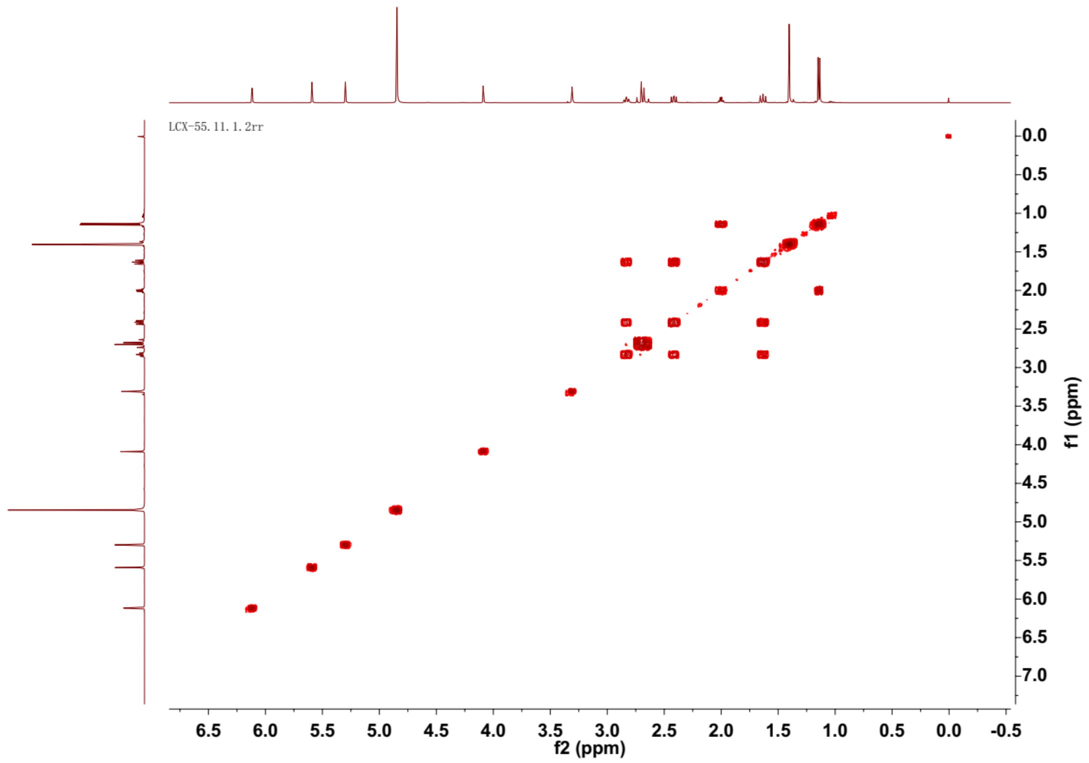

Figure S3. ^{1}H-^{1}H COSY spectrum of **1** in Methanol-d_4.

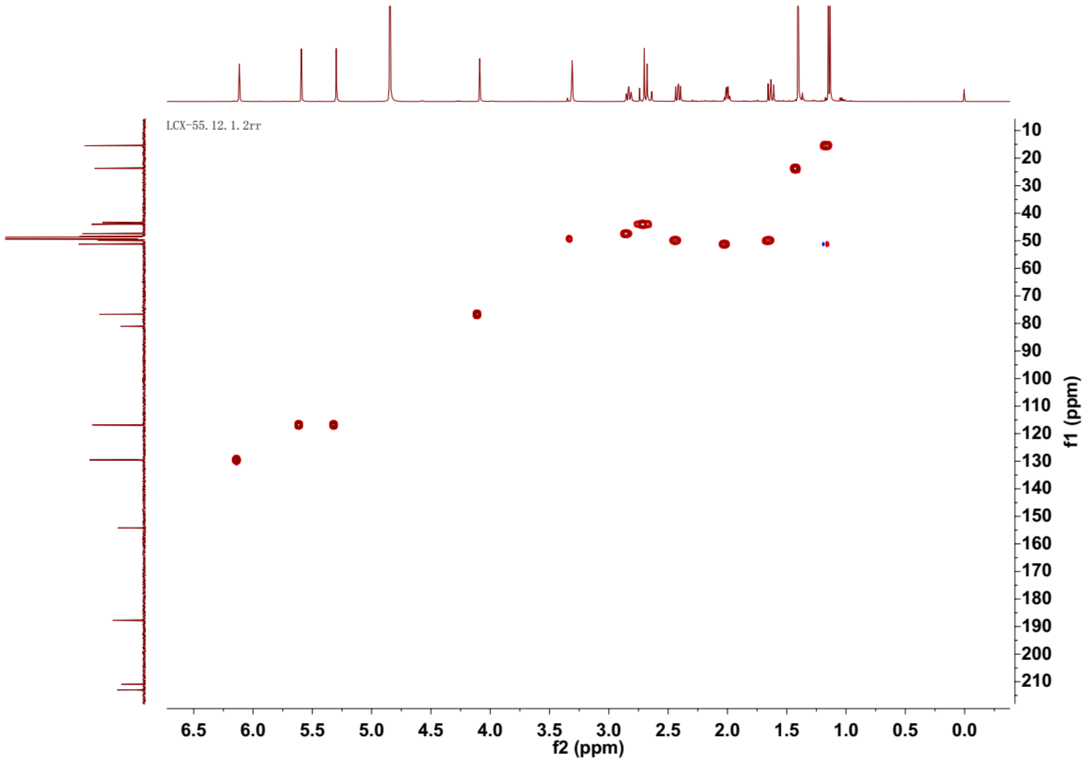

Figure S4. HSQC spectrum of **1** in Methanol-d_4.

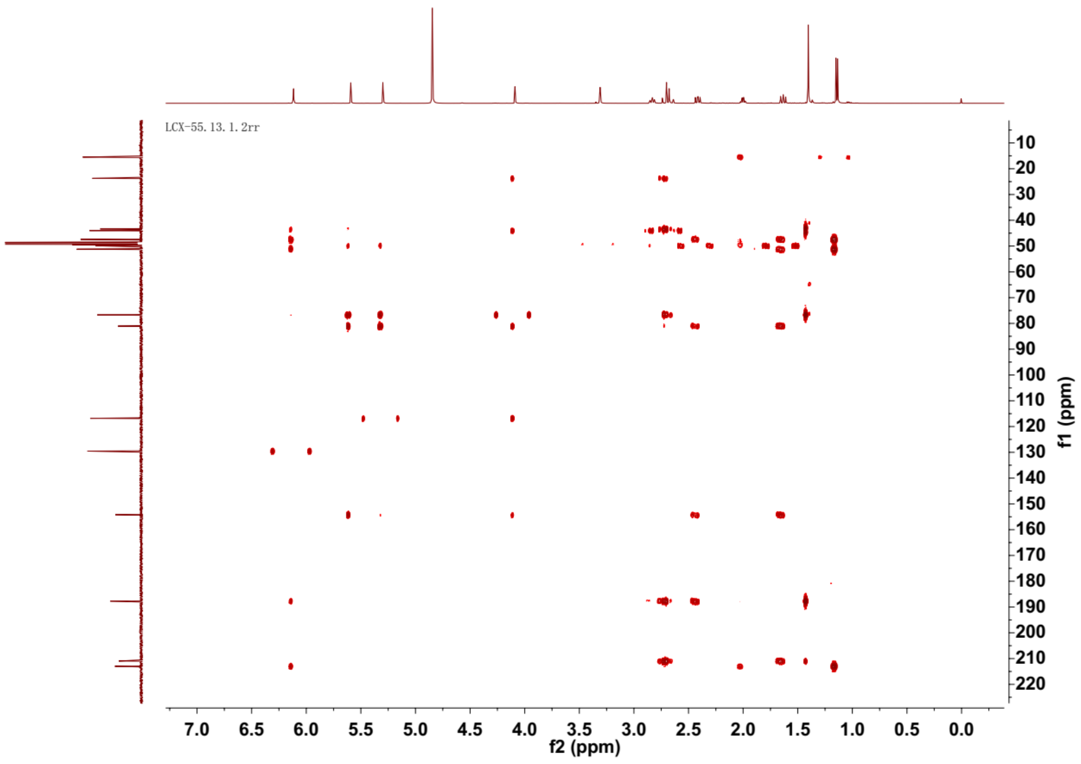

Figure S5. HMBC spectrum of **1** in Methanol-d_4.

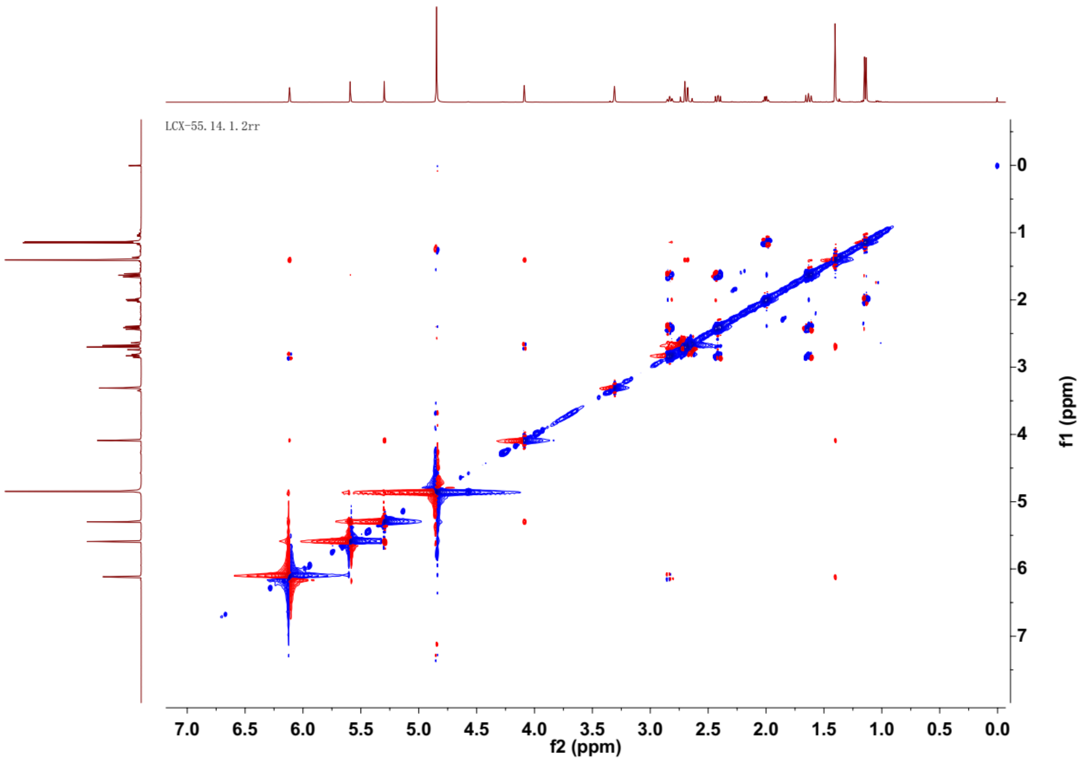

Figure S6. ROESY spectrum of **1** in Methanol-d_4.

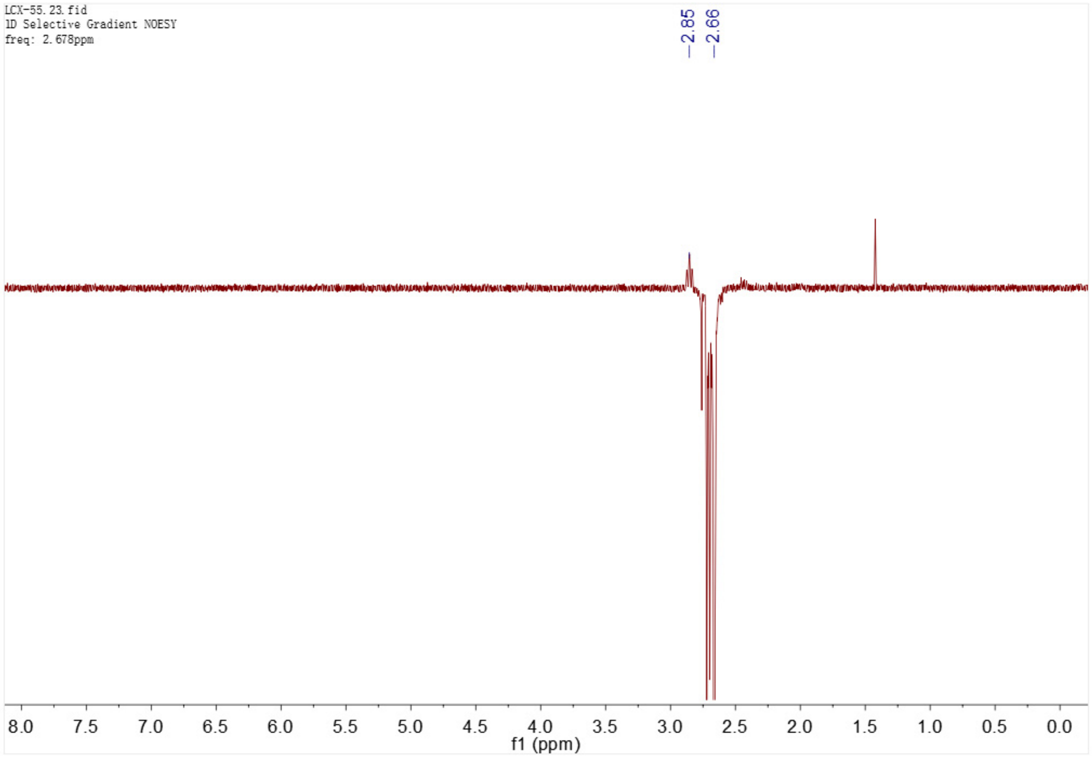

Figure S7. NOE spectrum of **1** in Methanol-d_4.

Created with SCIEX OS 1.4.1
Printed:18/1/2021 4:17:50 PM

Acquisition Date	18/1/2021 12:01:05 PM	Result Table	LCX-55
Acquisition Method	N/A	Algorithm Used	AutoPeak
Project	N/A	Instrument Name	X500 QTOF

Mass Spretra

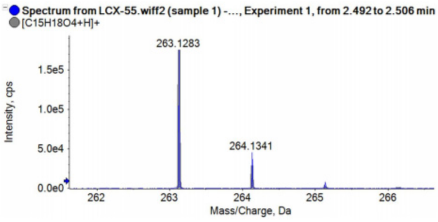

#	Analyte Peak Name	Formula	Precursor Mass	Found At Mass	Mass Error (ppm)
1	LCX-55	C15H18O4	263.1280	263.1283	2.1

Figure S8. HRESIMS of **1**.

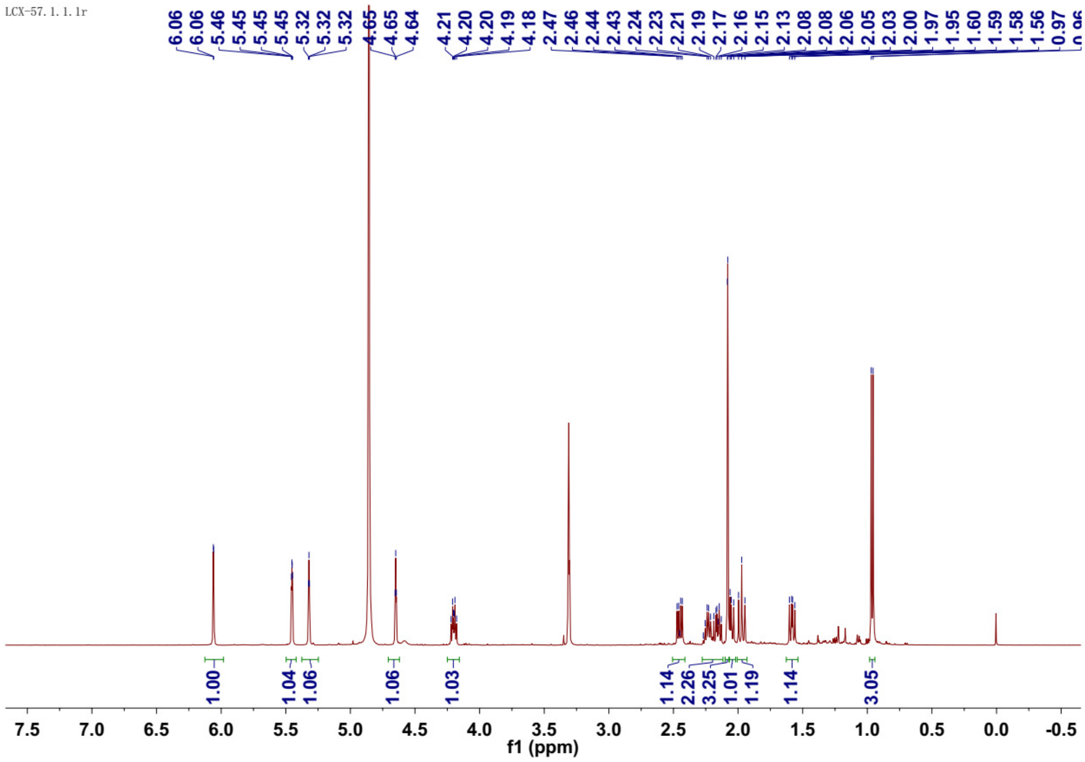

Figure S9.^{1}H NMR spectrum of **2** in Methanol-*d*4.

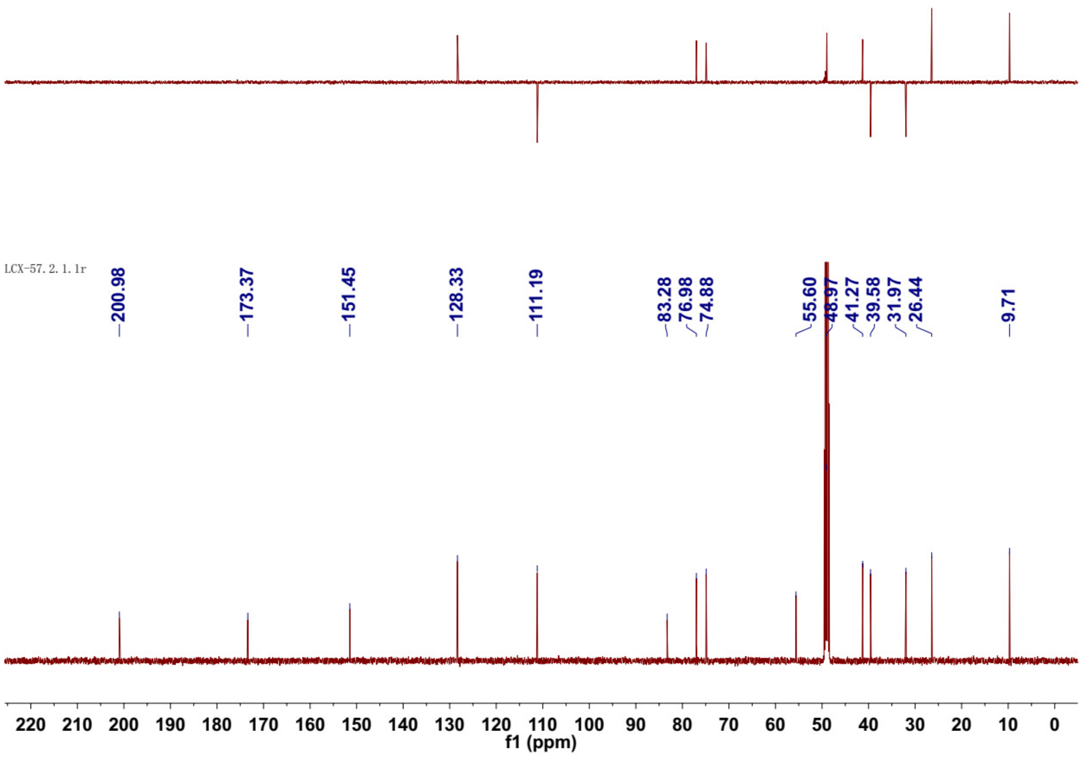

Figure S10.^{13}C NMR spectrum of **2** in Methanol-*d*4.

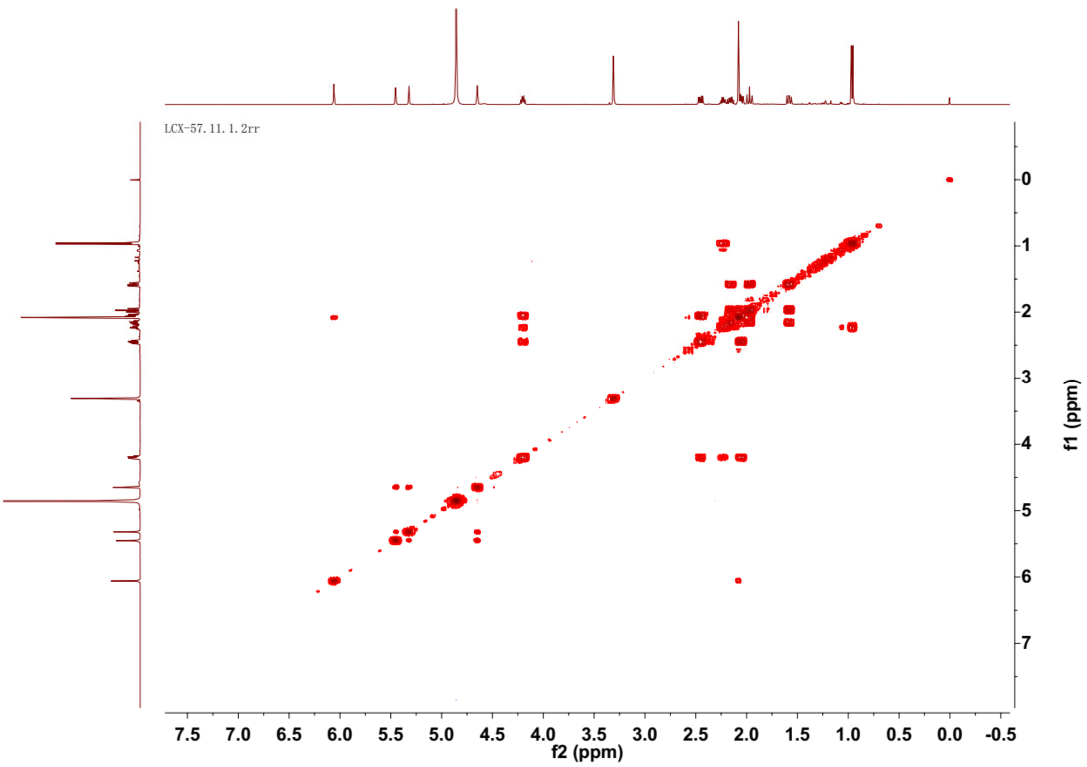

Figure S11. ^{1}H-^{1}H COSY spectrum of **2** in Methanol-d_4.

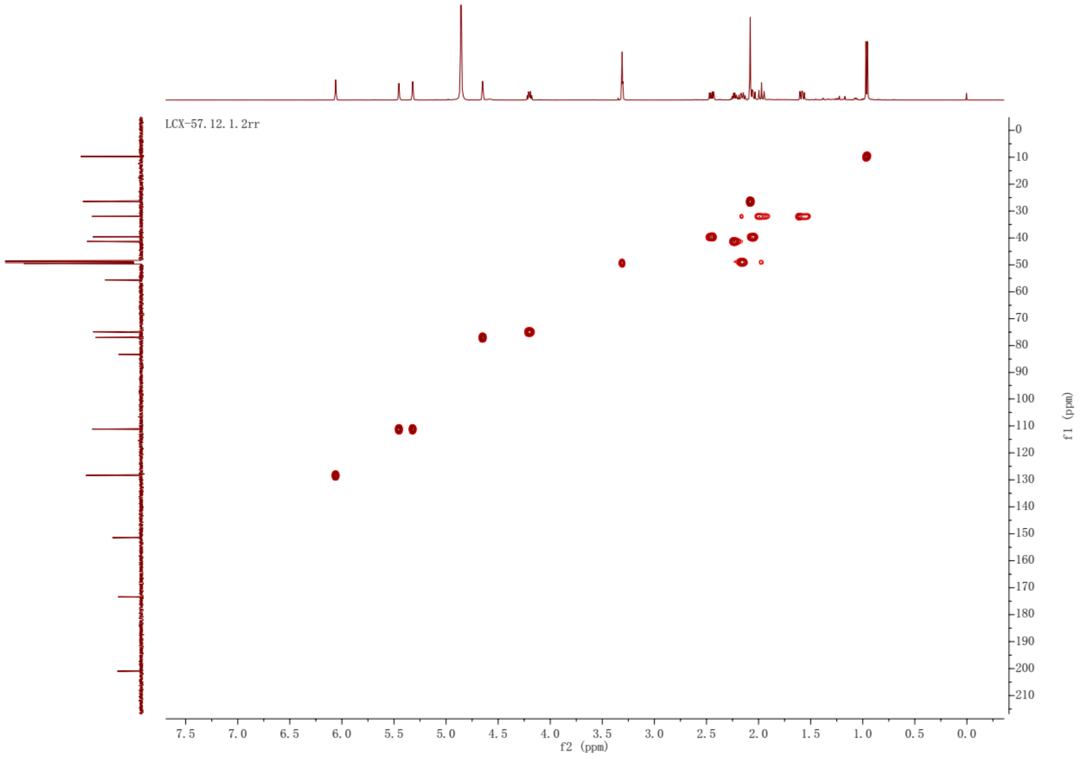

Figure S12. HSQC spectrum of **2** in Methanol-d_4.

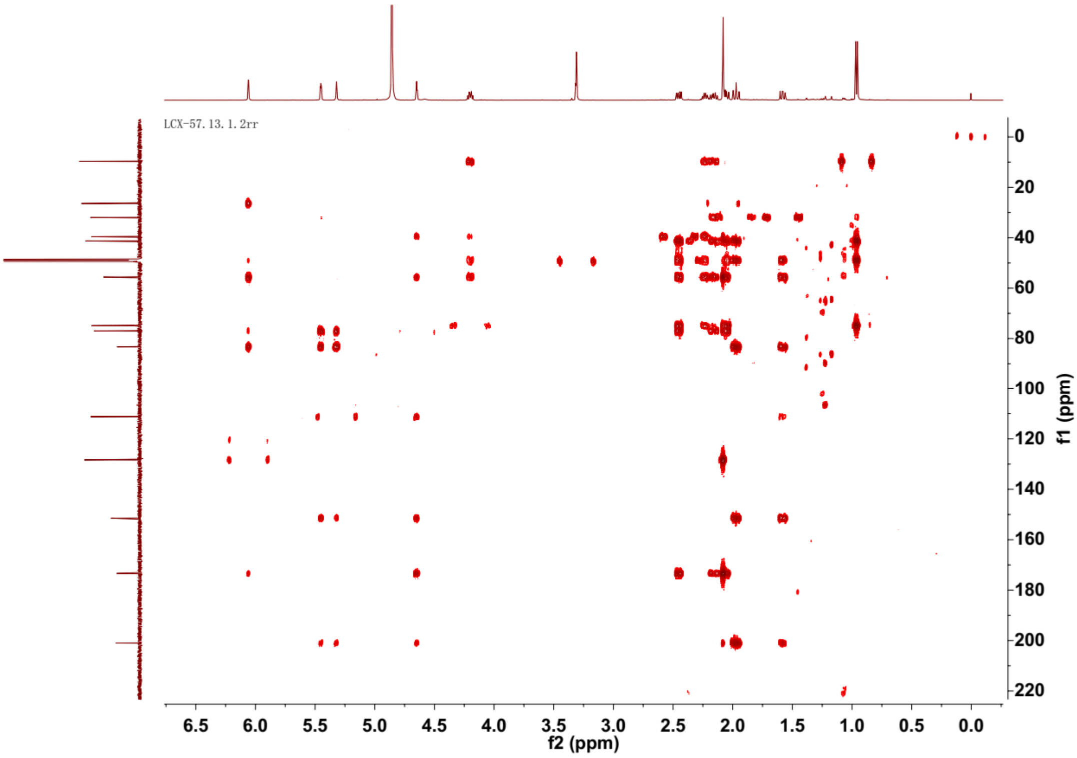

Figure S13. HMBC spectrum of **2** in Methanol-d_4.

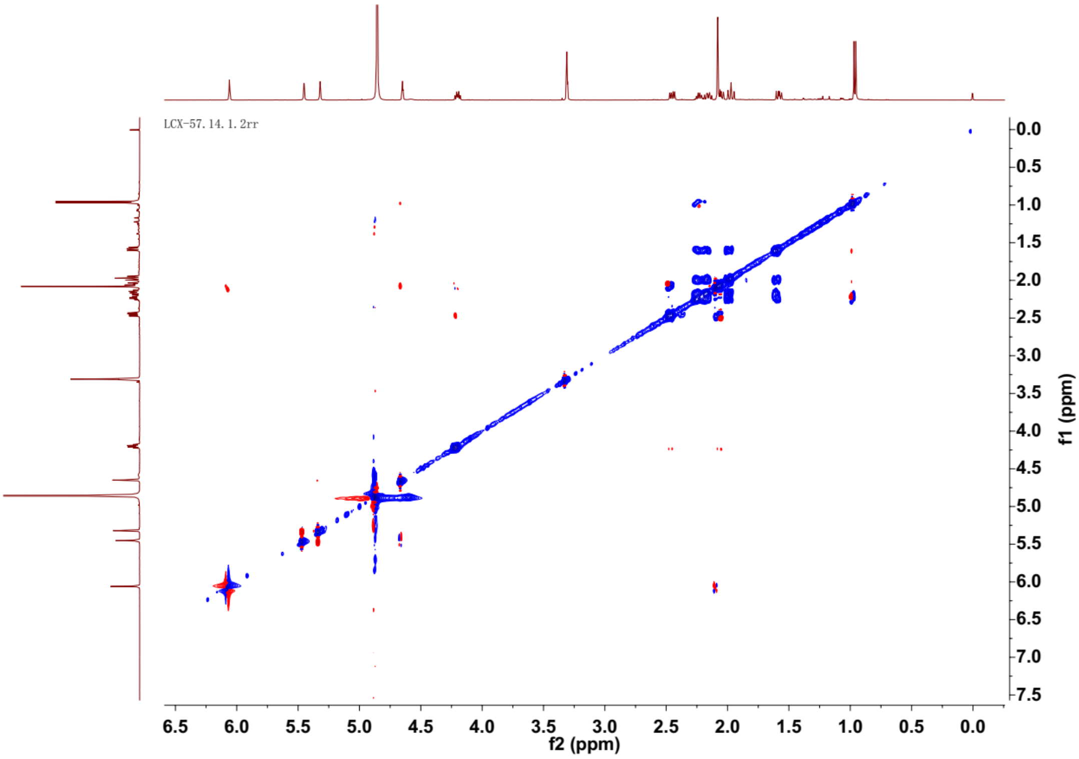

Figure S14. ROESY spectrum of **2** in Methanol-d_4.

Created with SCIEX OS 1.4.1
Printed:18/1/2021 4:23:31 PM

Acquisition Date	18/1/2021 12:11:37 PM	Result Table	LCX-57
Acquisition Method	N/A	Algorithm Used	AutoPeak
Project	N/A	Instrument Name	X500 QTOF

Mass Spretra

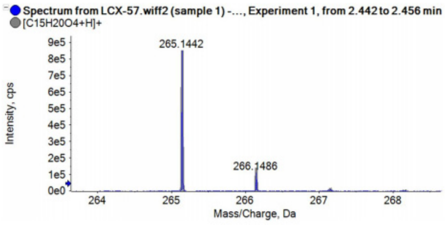

#	Analyte Peak Name	Formula	Precursor Mass	Found At Mass	Mass Error (ppm)
1	LCX-57	C15H20O4	265.1430	265.1442	2.9

Figure S15. HRESIMS of **2**.

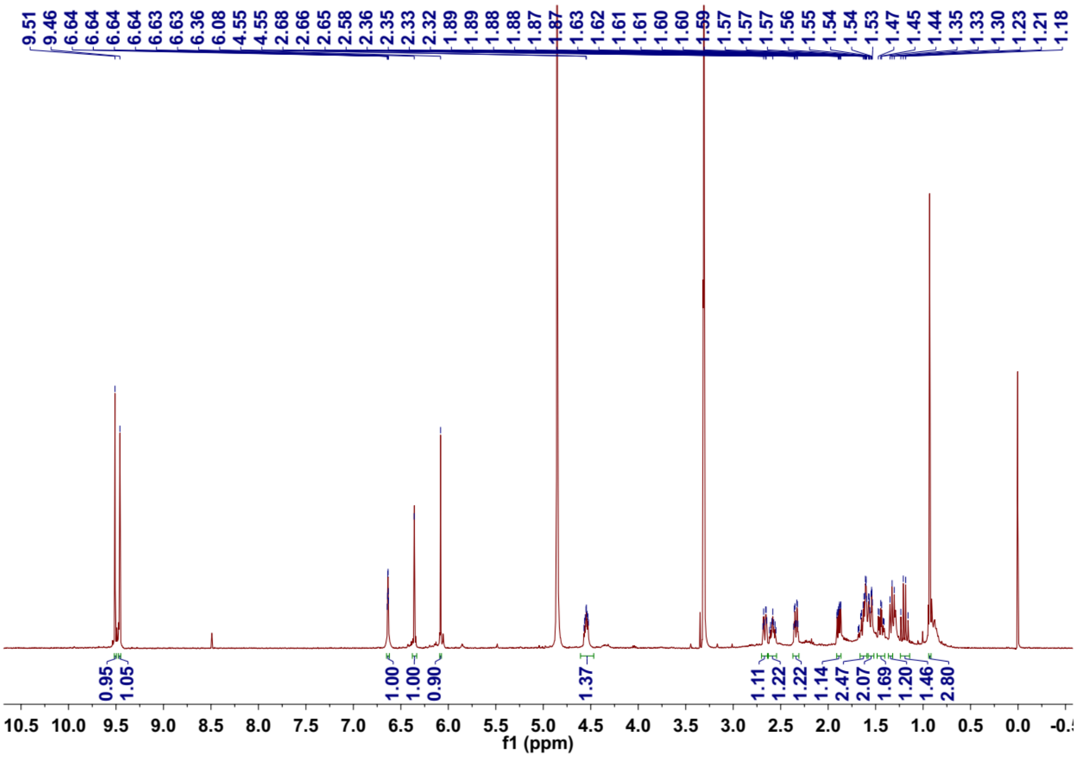

Figure S16.^{1}H NMR spectrum of **3** in Methanol-*d*4.

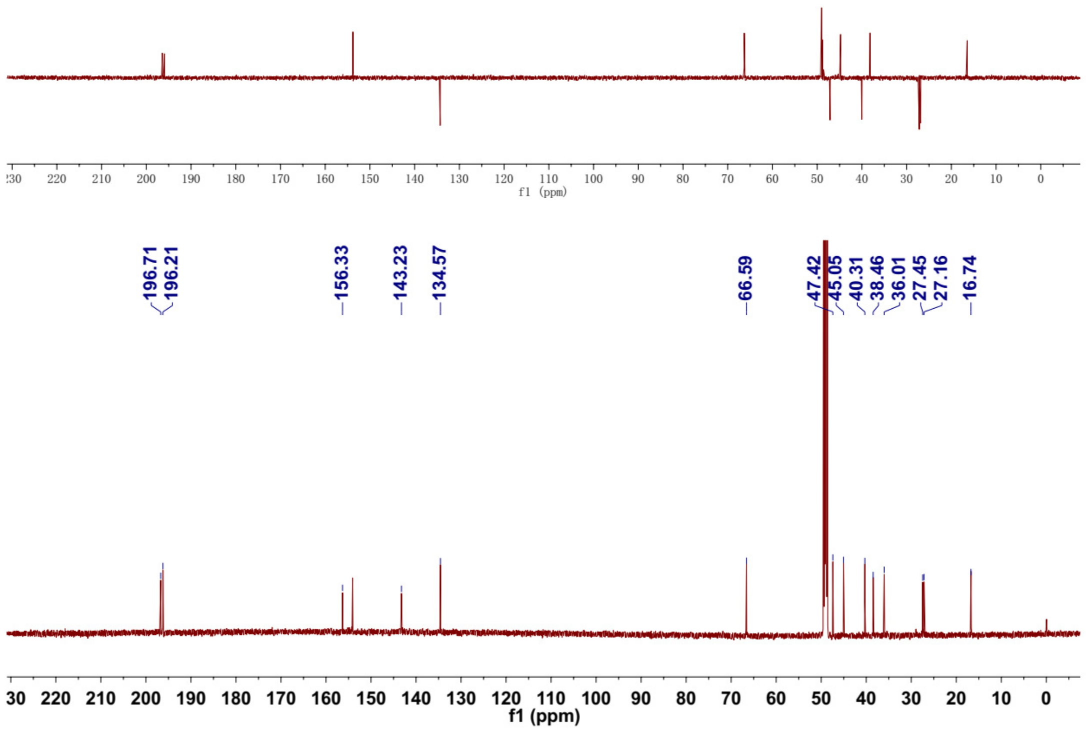

Figure S17. ^{13}C NMR and DEPT spectra of **3** in Methanol-d_4.

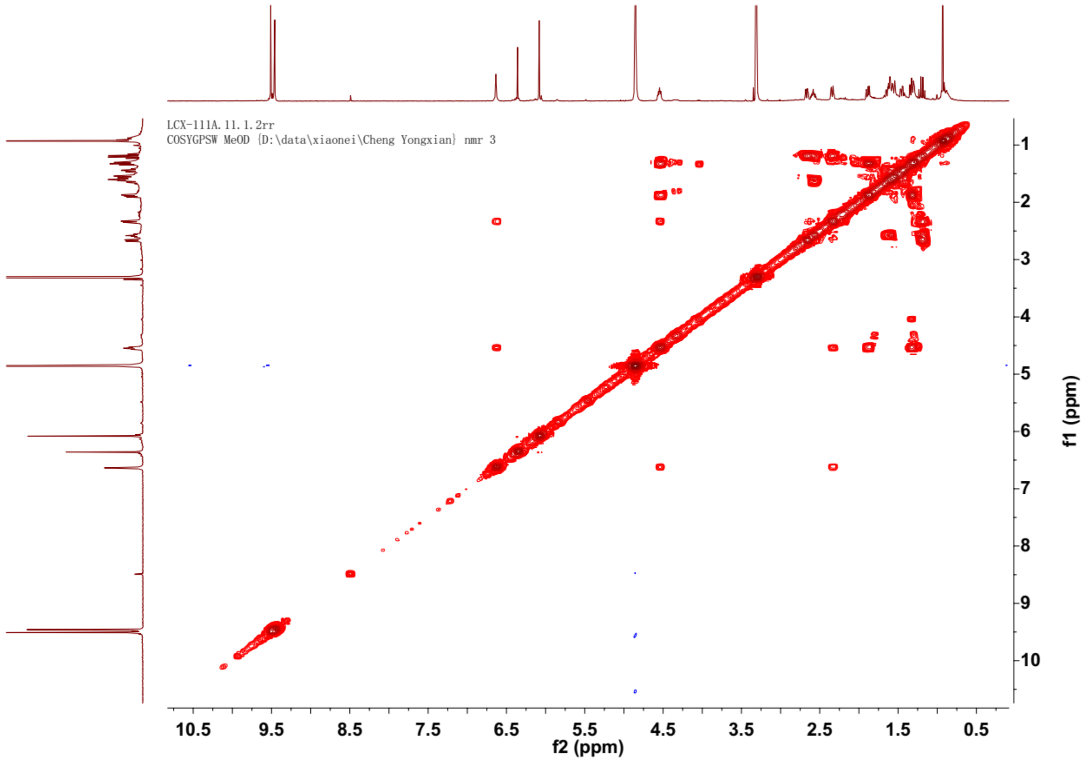

Figure S18. ^{1}H-^{1}H COSY spectrum of **3** in Methanol-d_4.

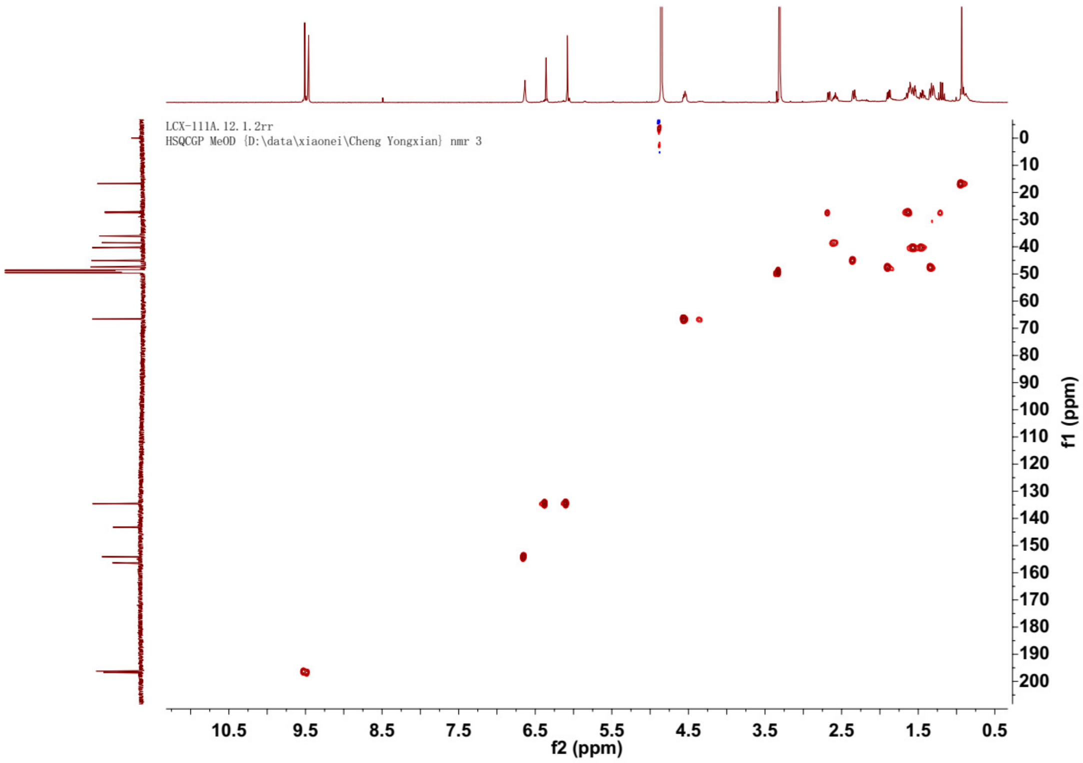

Figure S19. HSQC spectrum of **3** in Methanol-*d*4.

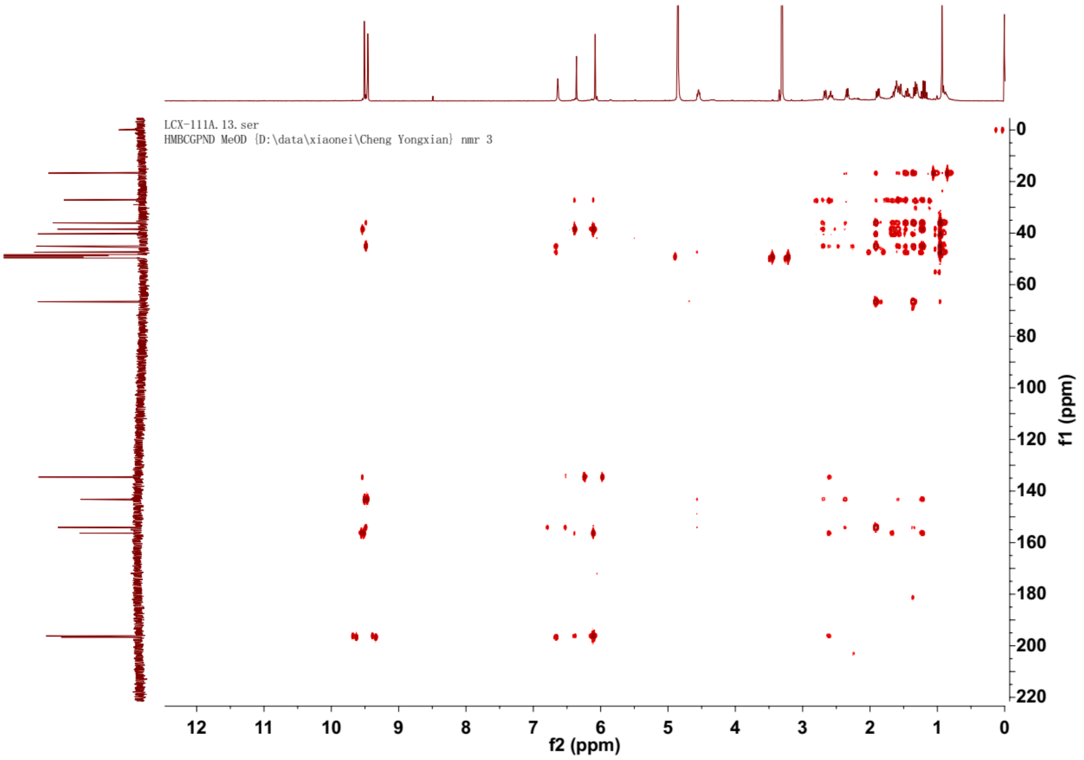

Figure S20. HMBC spectrum of **3** in Methanol-*d*4.

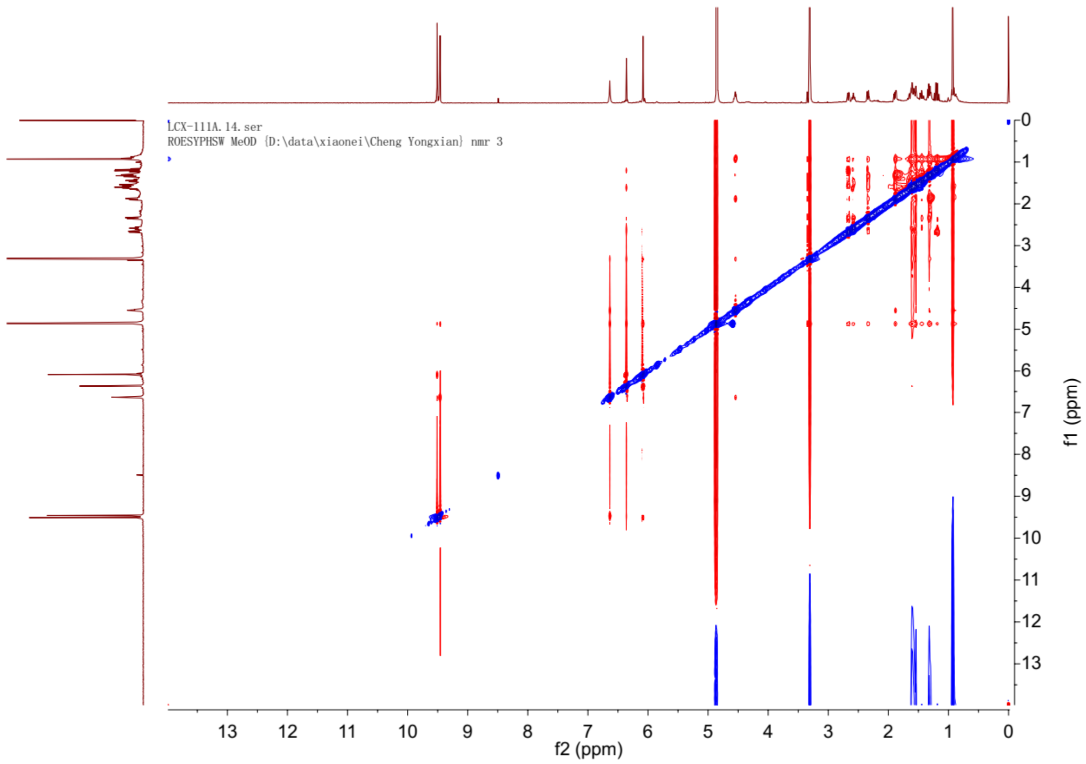

Figure S21. ROESY spectrum of **3** in Methanol-d_4.

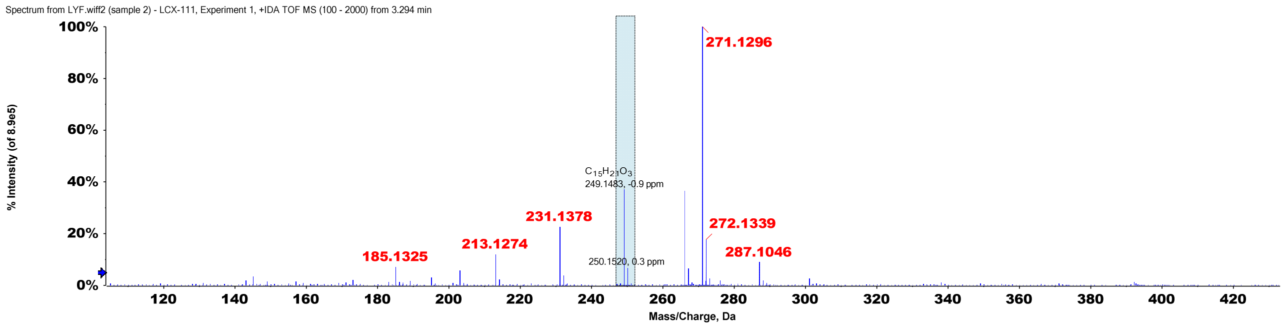

Hit	Formula	m/z	RDB	ppm	MS Rank	MSMS ppm	MSMS Rank	Found
1	C15H20O3	249.1485	6.0	-0.9	1			NA/NA

Figure S22. HRESIMS of **3**.

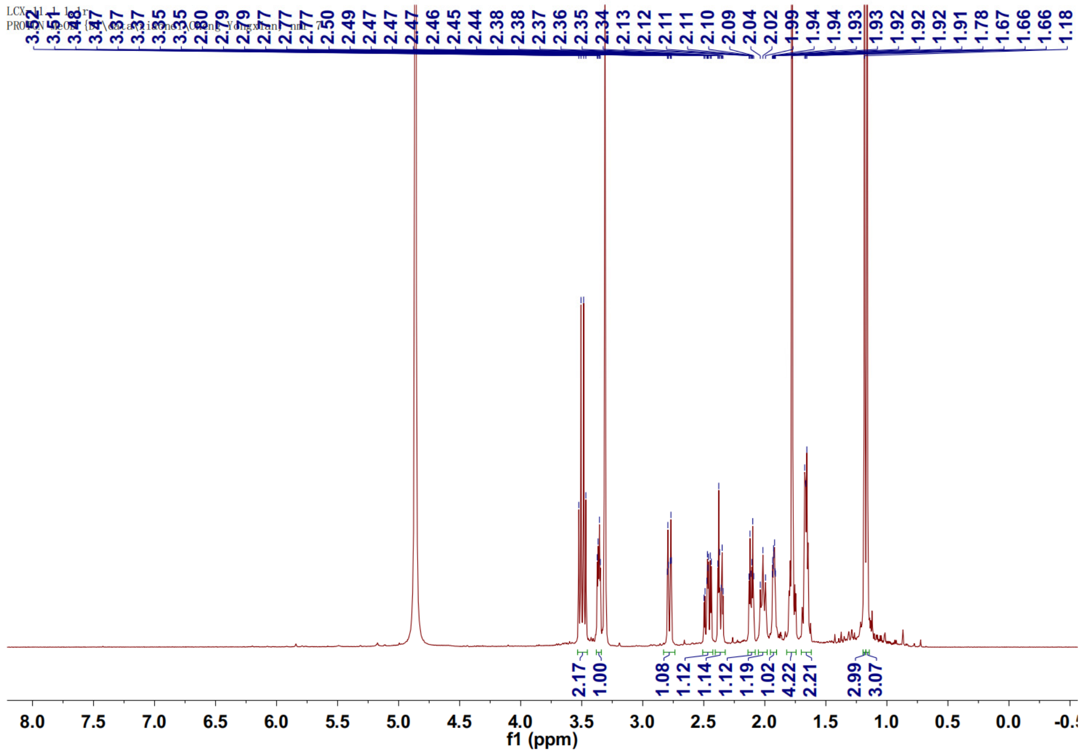

Figure S23. ^{1}H NMR spectrum of **4** in Methanol-*d₄*.

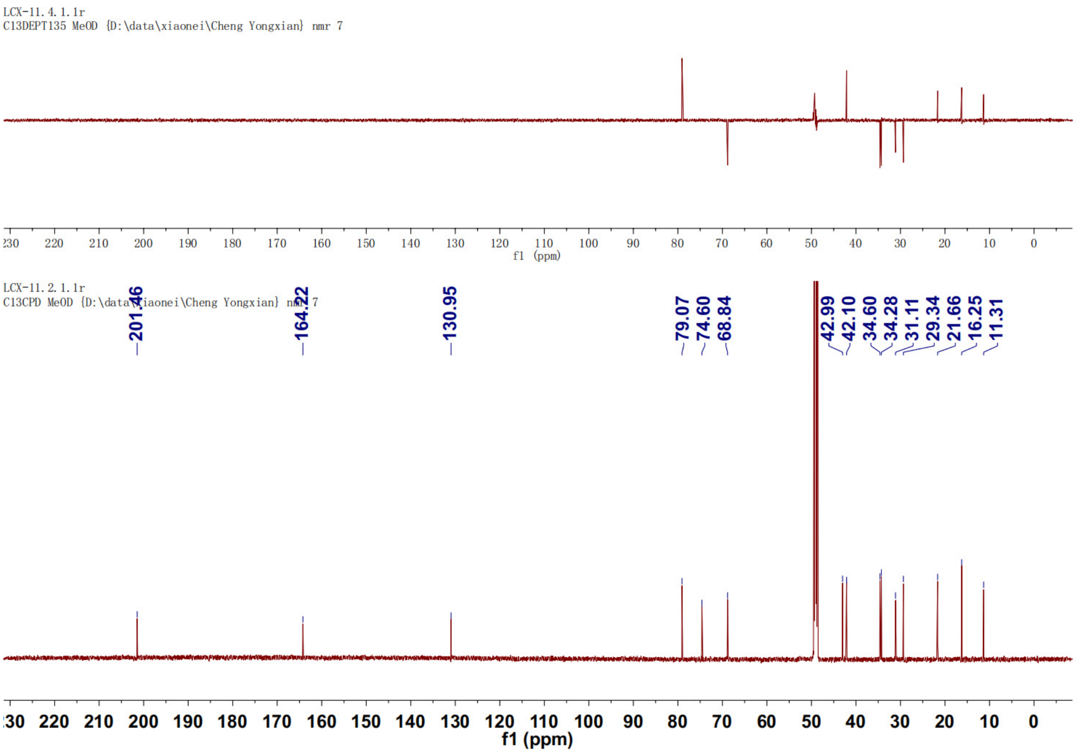

Figure S24. ^{13}C NMR and DEPT spectra of **4** in Methanol-*d₄*.

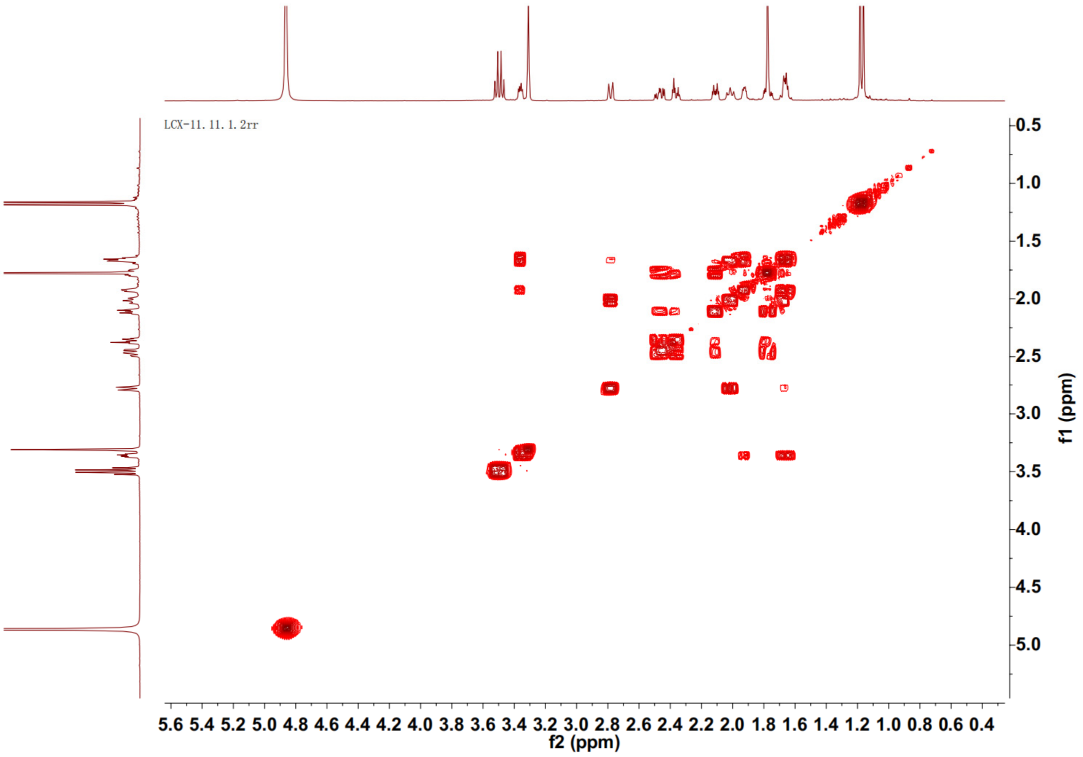

Figure S25. ^{1}H-^{1}H COSY spectrum of **4** in Methanol-d_4.

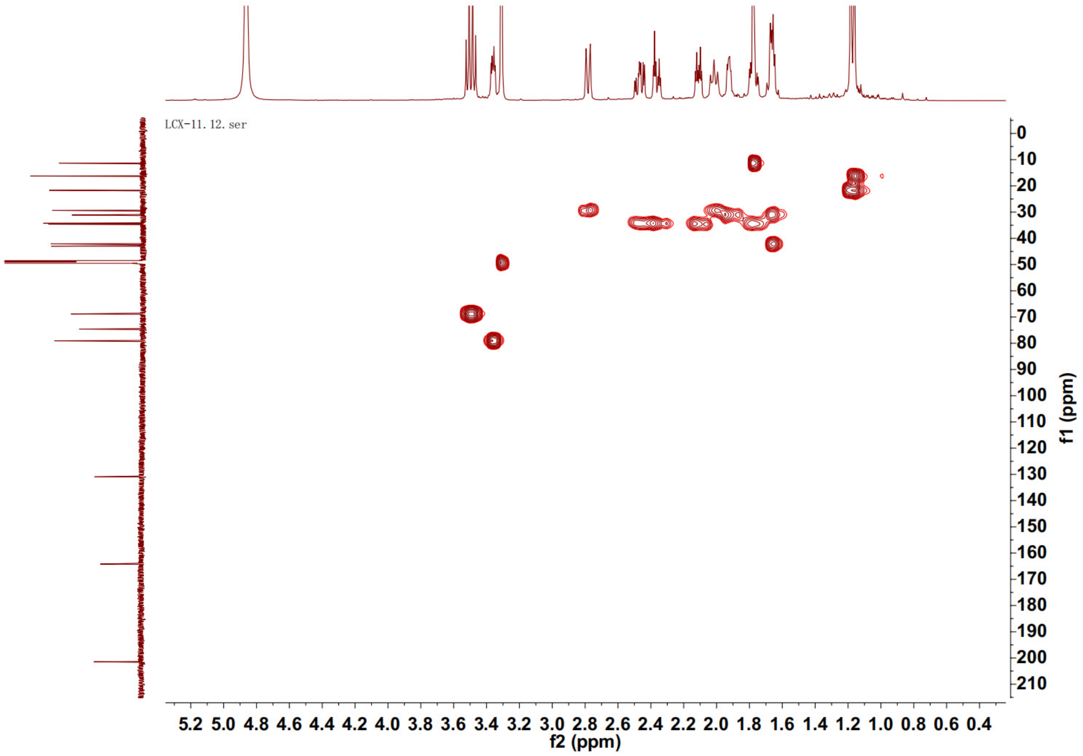

Figure S26. HSQC spectrum of **4** in Methanol-d_4.

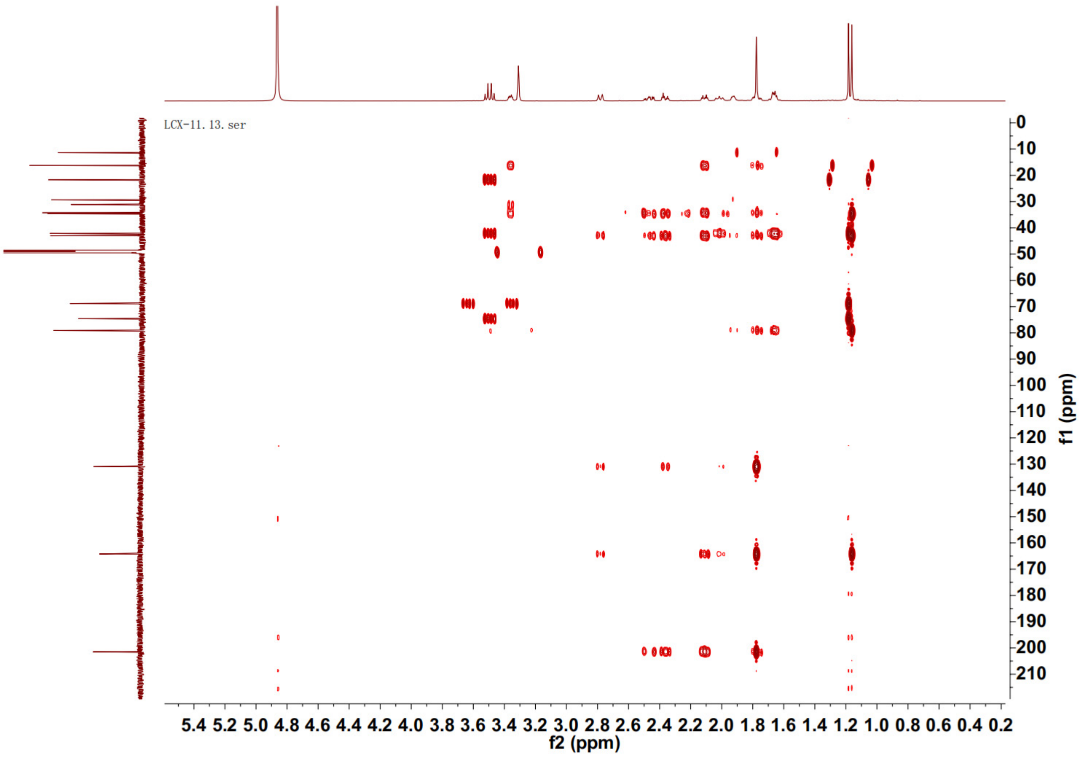

Figure S27. HMBC spectrum of **4** in Methanol-d_4.

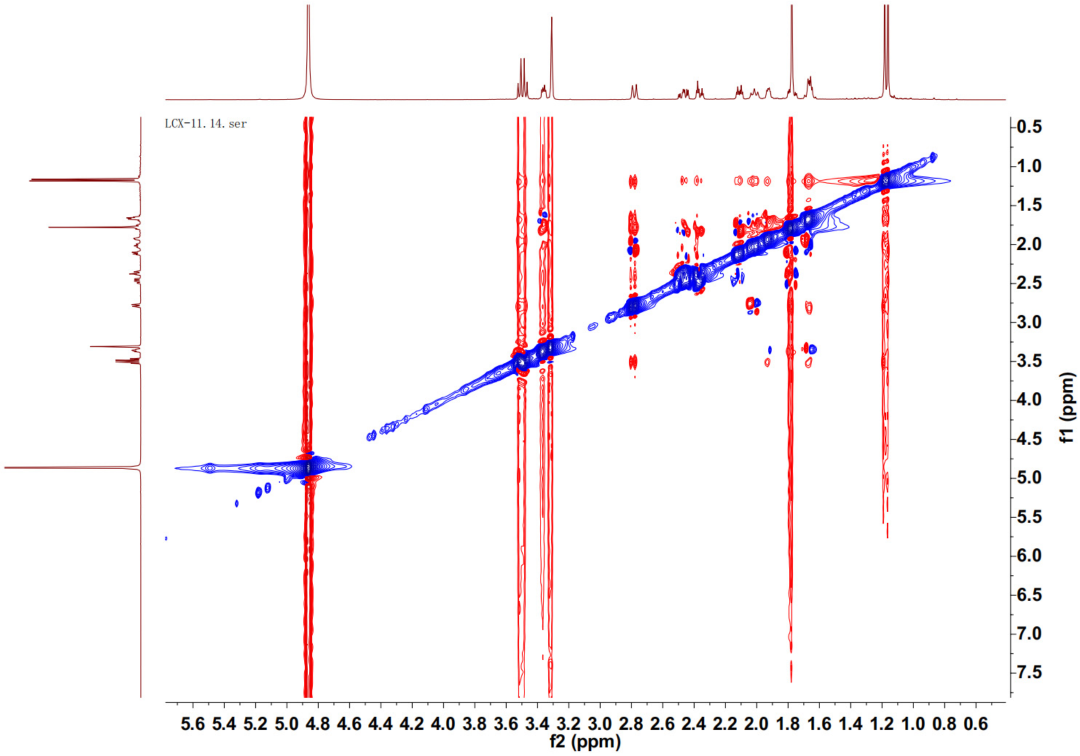

Figure S28. ROESY spectrum of **4** in Methanol-d_4.

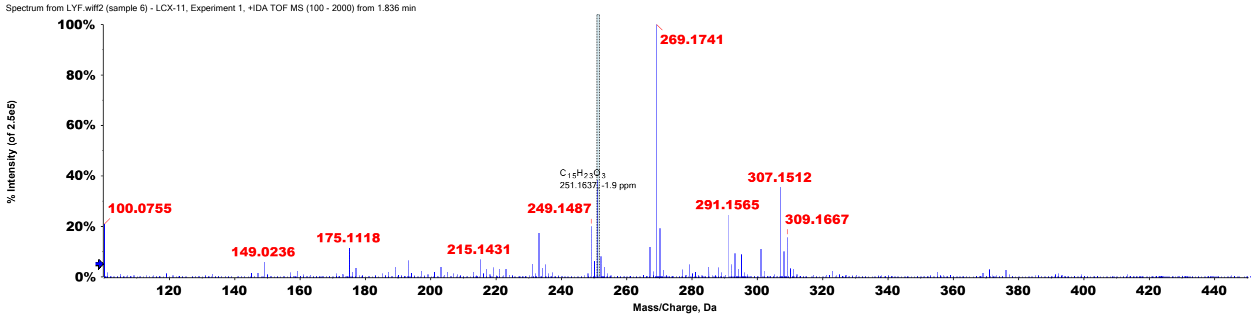

Hit	Formula	m/z	RDB	ppm	MS Rank	MSMS ppm	MSMS Rank	Found
1	C15H22O3	251.1642	5.0	-1.9	1			NA/NA

Figure S29. HRESIMS of **4**.

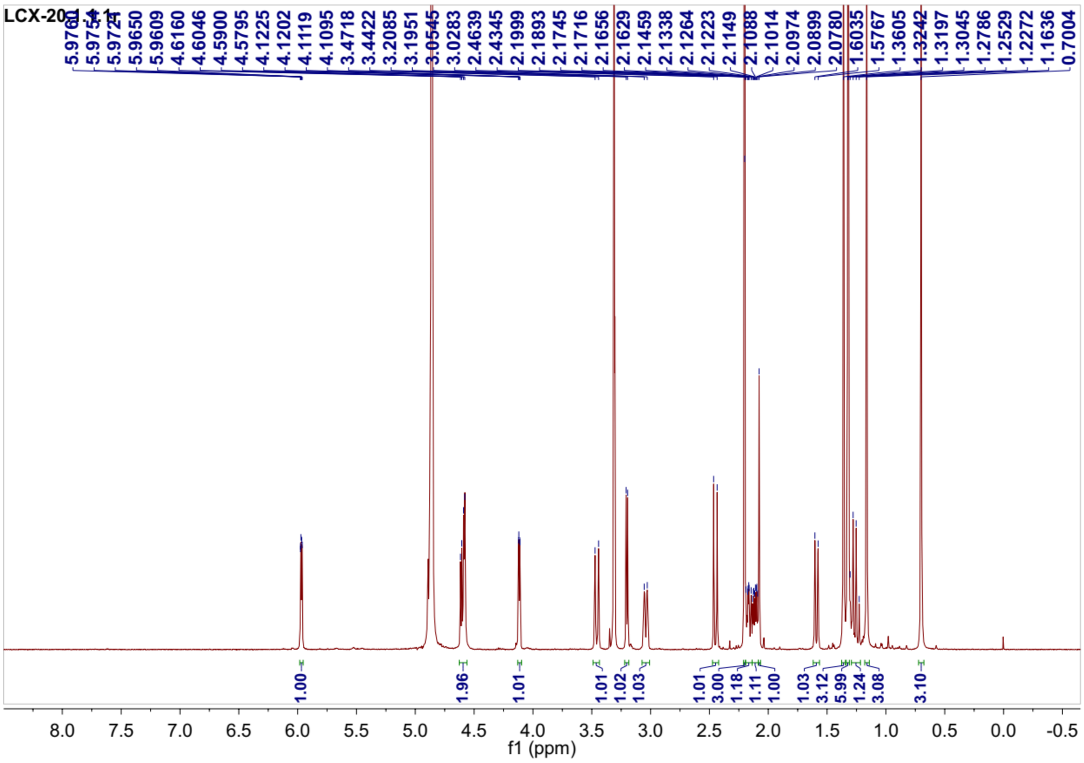

Figure S30.^{1}H NMR spectrum of **5** in Methanol-*d*4.

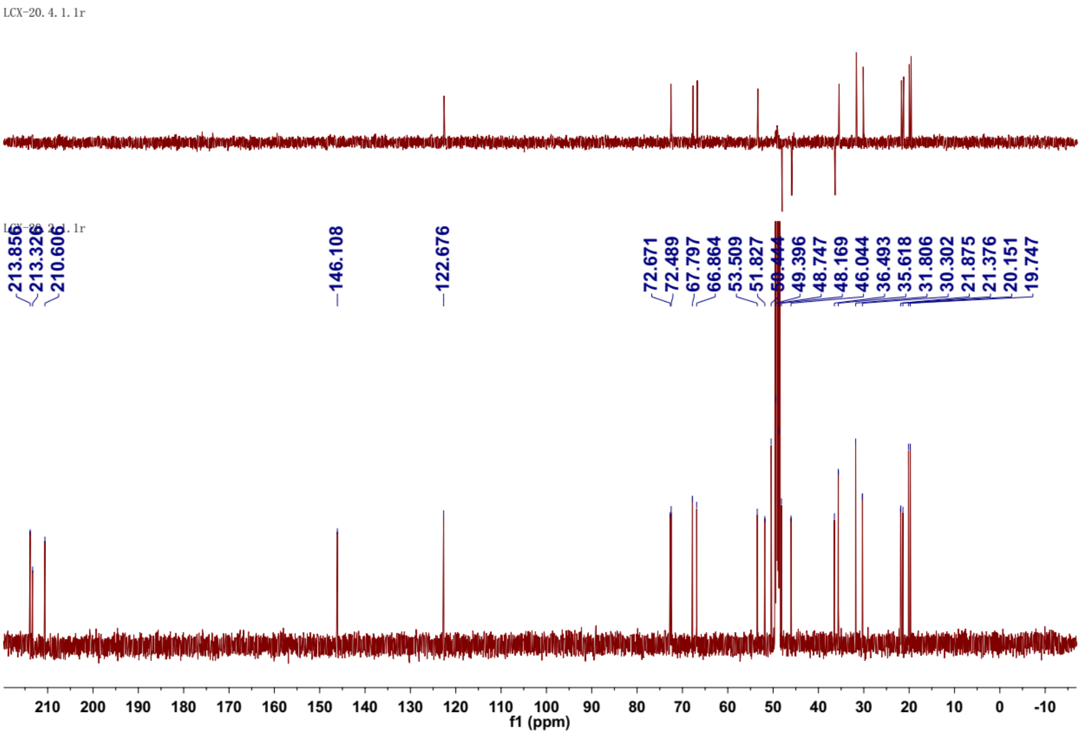

Figure S31. ^{13}C NMR and DEPT spectra of **5** in Methanol-*d4*.

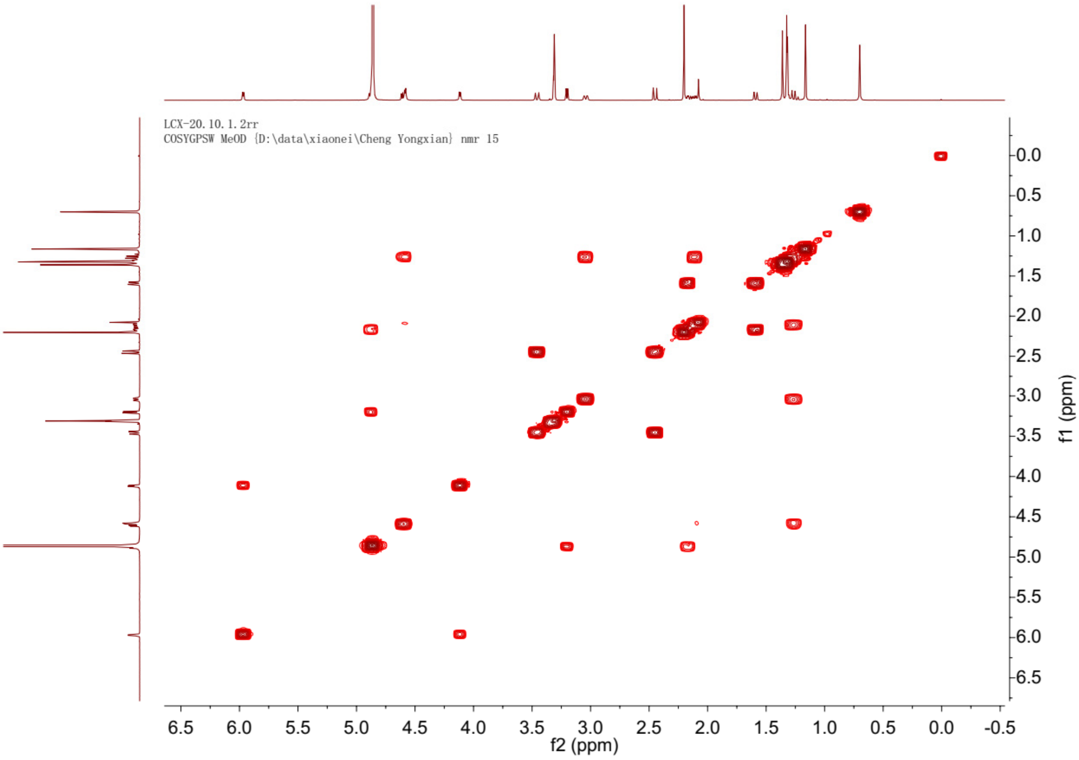

Figure S32. HSQC spectrum of **5** in Methanol-*d4*.

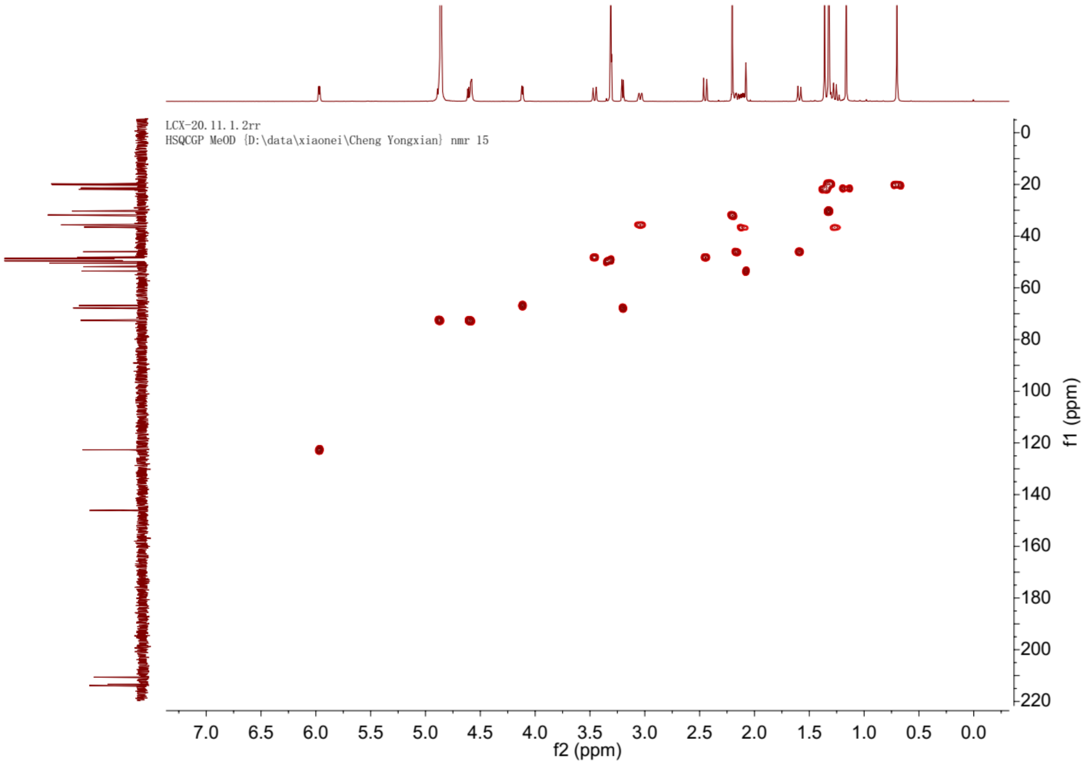

Figure S33. HSQC spectrum of **5** in Methanol-d_4.

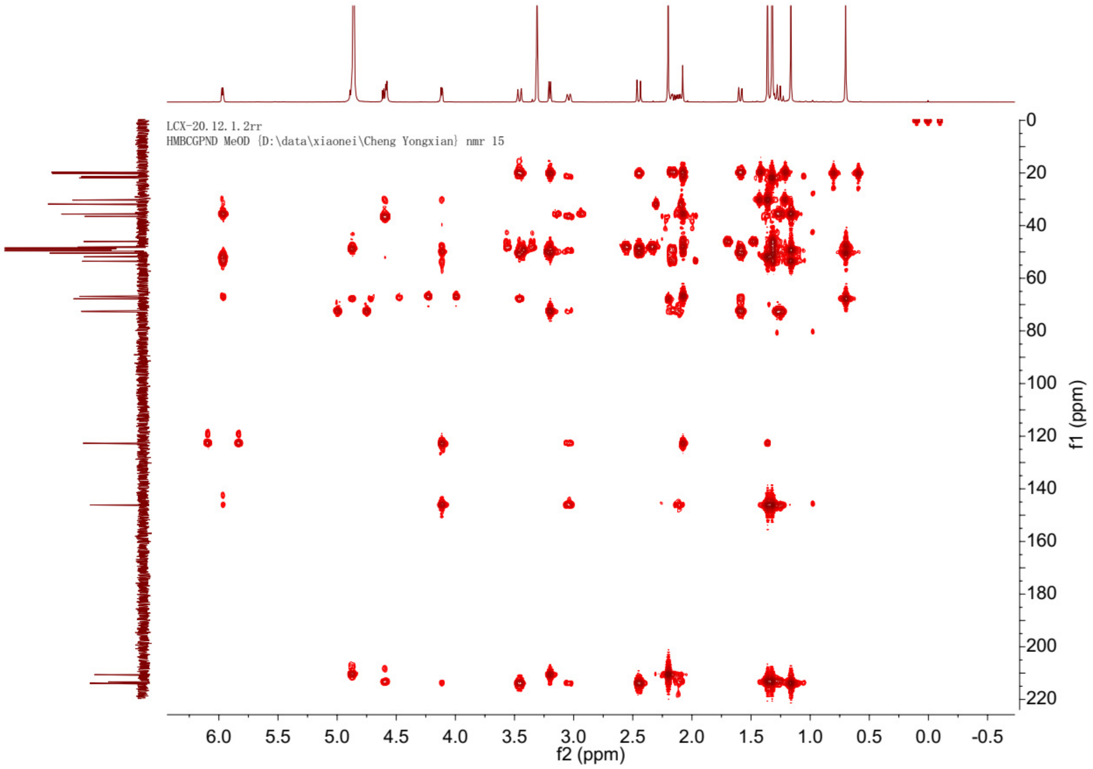

Figure S34. HMBC spectrum of **5** in Methanol-d_4.

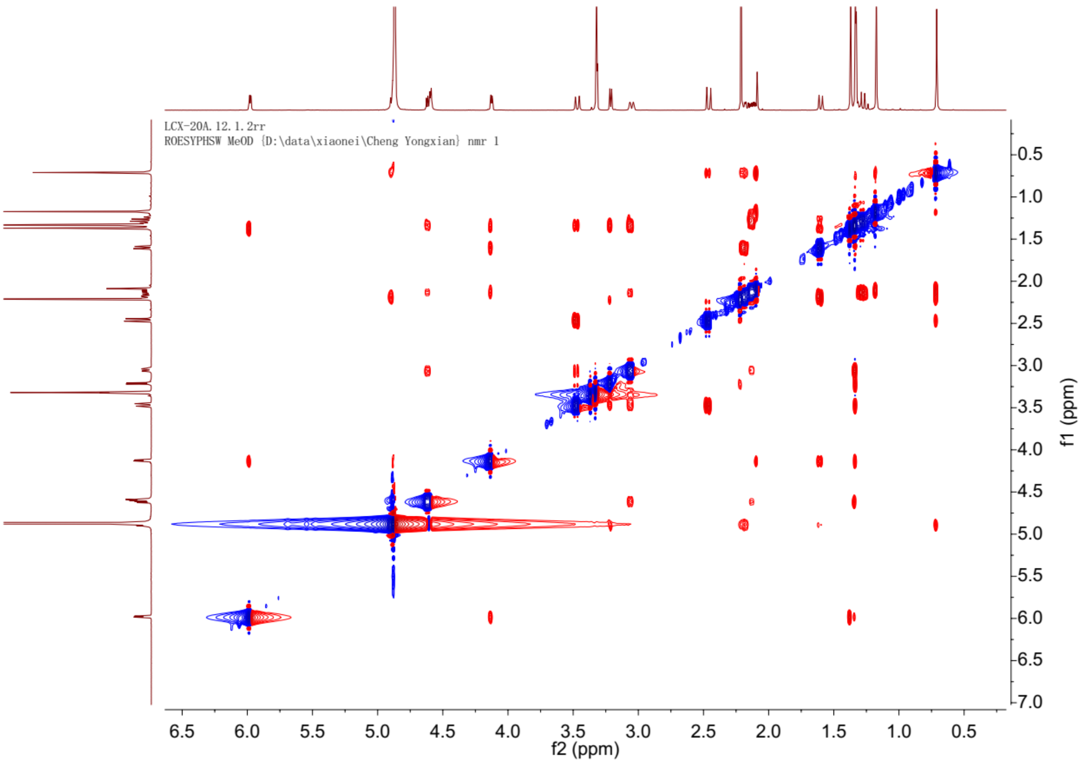

Figure S35. ROESY spectrum of **5** in Methanol-d_4.

SCIEX

Created with SCIEX OS 1.4.1
Printed:18/1/2021 4:13:29 PM

Acquisition Date	18/1/2021 11:50:32 AM	Result Table	LCX-20
Acquisition Method	N/A	Algorithm Used	AutoPeak
Project	N/A	Instrument Name	X500 QTOF

Mass Spretra

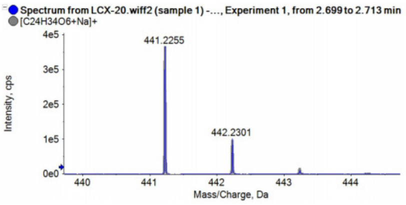

#	Analyte Peak Name	Formula	Precursor Mass	Found At Mass	Mass Error (ppm)
1	LCX-20	C24H34O6	441.2250	441.2255	1.6

Figure S36. HRESIMS of **5**.

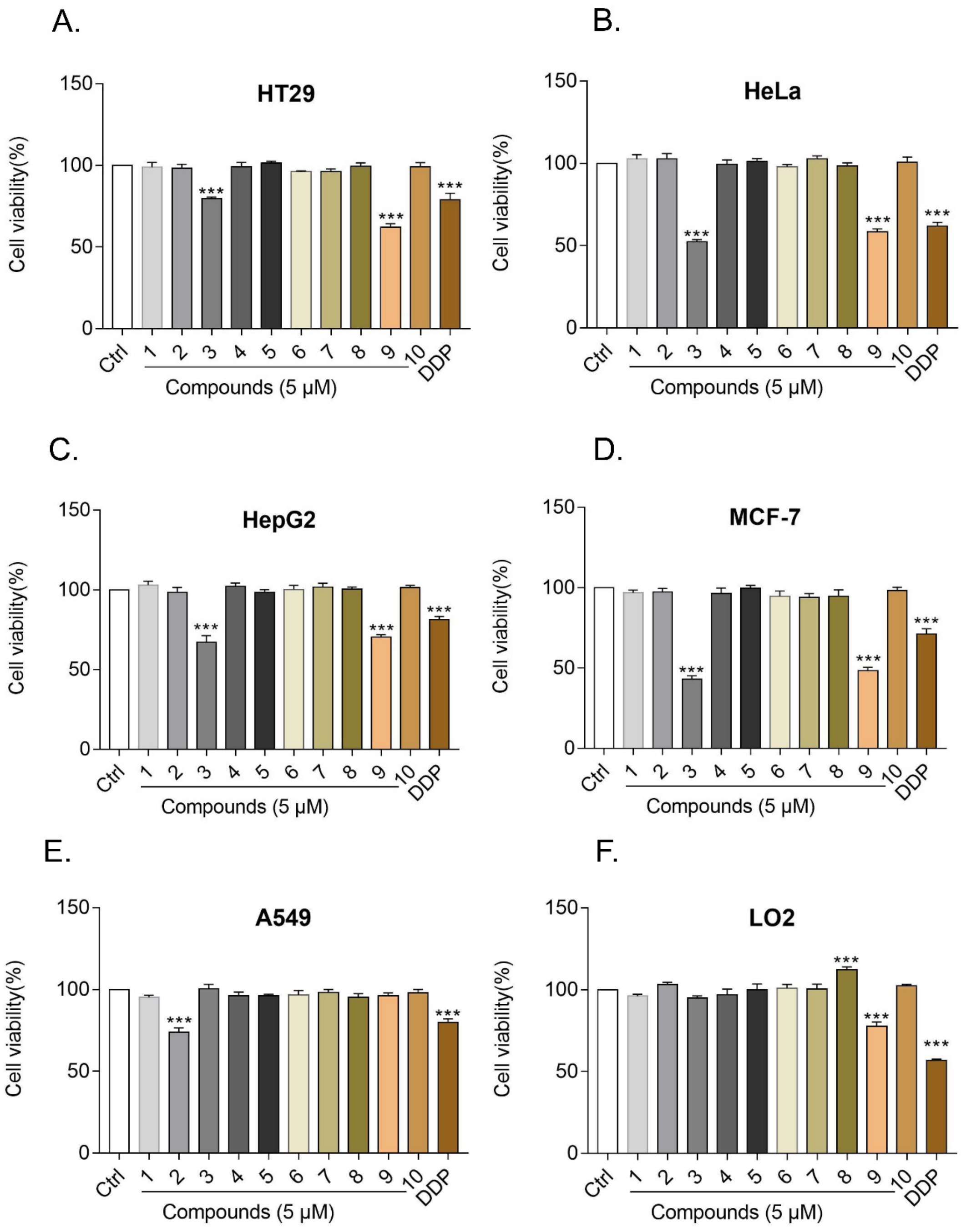

Figure S37. Effects of Compound 1-10 and DDP on cell viability in human cancer cells. HT29, HeLa, HepG2, MCF-7, A549, and LO2 cells were treated with 5μM compound **1-10** or DDP for 24 h followed by MTT assay. ***$p < 0.001$; *$p < 0.05$ is defined as statistical significance.

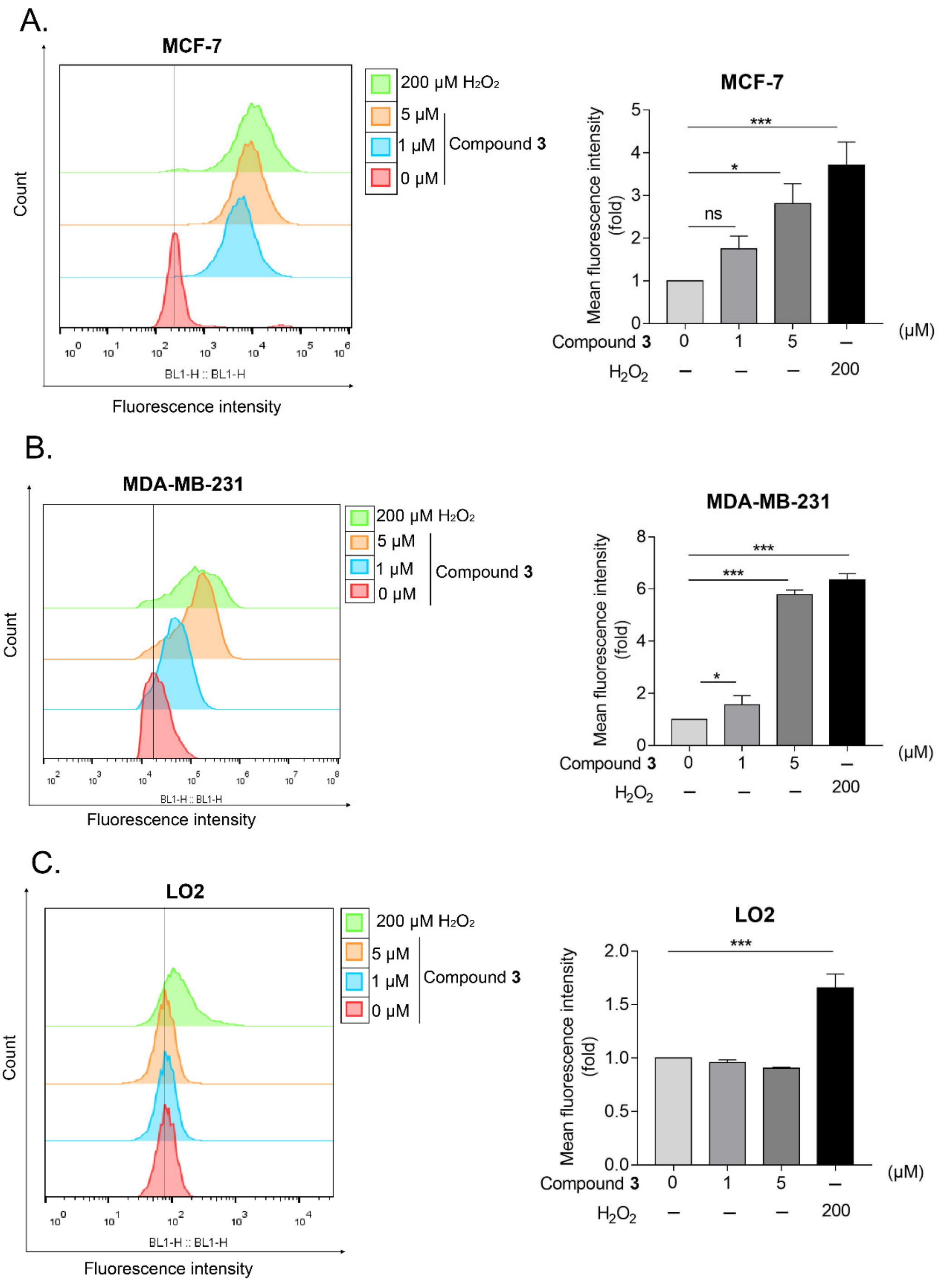

Figure S38. Compound 3 promoted the production of reactive oxygen species in human breast cancer cells. MCF-7 (A), MDA-MB-231 (B), and LO2 cells (C) were treated with indicated concentration of compound 3 or H2O2 for 24 h. Then the ROS detection kit was performed to measure the intracellular ROS level and the fluorescence intensity in the cells was detected by flow cytometry and the bar chart showed relative mean fluorescence intensity by FlowJo software. *$p < 0.05$, ***$p < 0.001$; *$p < 0.05$ is defined as statistical significance, ns represents non-significant effects.

Crystal structure determination of 1

Crystal Data for $C_{15}H_{18}O_4$ (*M* =262.29 g/mol): monoclinic, space group $P2_1$ (no. 4), *a* = 8.5199(2) Å, *b* = 8.1291(2) Å, *c* = 9.3413(2) Å, *β* = 92.277(2)°, *V* = 646.46(3) Å^3, *Z* = 2, *T* = 169.99(10) K, μ(Cu Kα) = 0.797 mm^{-1}, *Dcalc* = 1.347 g/cm^3, 6394 reflections measured (9.476° ≤ 2Θ ≤ 147.736°), 2411 unique (R_{int} = 0.0324, R_{sigma} = 0.0265) which were used in all calculations. The final R_1 was 0.0455 (I > 2σ(I)) and wR_2 was 0.1164 (all data).

Table S1 Crystal data and structure refinement for 1.

Identification code	55
Empirical formula	$C_{15}H_{18}O_4$
Formula weight	262.29
Temperature/K	169.99(10)
Crystal system	monoclinic
Space group	$P2_1$
a/Å	8.5199(2)
b/Å	8.1291(2)
c/Å	9.3413(2)
α/°	90
β/°	92.277(2)
γ/°	90
Volume/Å^3	646.46(3)
Z	2
ρ_{calc}g/cm^3	1.347
μ/mm^{-1}	0.797
F(000)	280.0
Crystal size/mm^3	0.14 × 0.12 × 0.1
Radiation	Cu Kα (λ = 1.54184)
2Θ range for data collection/°	9.476 to 147.736
Index ranges	-10 ≤ h ≤ 7, -9 ≤ k ≤ 9, -11 ≤ l ≤ 11
Reflections collected	6394
Independent reflections	2411 [R_{int} = 0.0324, R_{sigma} = 0.0265]
Data/restraints/parameters	2411/1/184
Goodness-of-fit on F^2	1.164
Final R indexes [I>=2σ (I)]	R_1 = 0.0455, wR_2 = 0.1155
Final R indexes [all data]	R_1 = 0.0459, wR_2 = 0.1164
Largest diff. peak/hole / e Å^{-3}	0.25/-0.37
Flack/Hooft parameter	0.02(15)/0.07(6)

Table S2 Fractional Atomic Coordinates (×10^4) and Equivalent Isotropic Displacement Parameters (Å^2×10^3) for 1. U_{eq} is defined as 1/3 of the trace of the orthogonalised U_{IJ} tensor.

Atom	*x*	*y*	*z*	U(eq)
O1	3032(2)	5291(3)	4748(2)	31.4(5)
O2	11030(2)	4158(3)	6905(2)	29.0(5)
O3	11204(2)	7329(3)	7776(2)	35.4(5)
O4	8415(2)	5296(3)	10454.0(19)	29.1(5)
C1	6235(3)	6149(3)	7042(3)	17.6(5)
C2	4696(3)	5877(3)	6819(3)	22.5(6)
C3	4343(3)	5507(4)	5312(3)	23.1(6)
C4	5878(3)	5431(3)	4546(3)	20.7(5)
C5	7112(3)	6099(3)	5655(2)	17.6(5)
C6	8670(3)	5136(4)	5674(3)	22.9(6)
C7	9607(3)	4993(3)	7131(3)	20.0(5)
C8	9899(3)	6714(4)	7736(3)	21.7(6)
C9	8502(3)	7630(3)	8279(3)	21.2(6)
C10	7045(3)	6552(3)	8471(2)	18.4(5)
C11	8649(3)	3977(4)	8154(3)	20.1(5)
C12	8734(4)	2355(4)	8184(3)	31.1(6)
C13	7588(3)	4903(3)	9138(3)	20.3(5)
C14	5933(3)	7444(4)	9456(3)	27.0(6)
C15	5799(4)	6293(5)	3104(3)	31.4(7)

Table S3 Anisotropic Displacement Parameters (Å^2×10^3) for 1. The Anisotropic displacement factor exponent takes the form: $-2\pi^2[h^2a^{*2}U_{11}+2hka^*b^*U_{12}+\ldots]$.

Atom	U_{11}	U_{22}	U_{33}	U_{23}	U_{13}	U_{12}
O1	21.0(9)	43.7(13)	29.2(10)	3.7(9)	-3.7(7)	-4.0(9)
O2	16.6(8)	32.3(12)	38.4(11)	-1.8(9)	7.2(8)	5.4(8)
O3	22.1(10)	37.9(13)	46.6(12)	-11.1(11)	8.1(8)	-9.8(9)
O4	39.9(11)	30.5(12)	16.5(8)	1.7(8)	-3.7(8)	6.8(10)
C1	18.9(11)	16.9(12)	17.3(11)	2.2(9)	5.3(9)	2.2(9)
C2	19.5(12)	27.1(15)	21.2(12)	2.9(10)	5.2(10)	1.6(10)
C3	21.0(12)	23.3(15)	25.0(13)	3.0(10)	0.4(10)	-0.9(10)
C4	23.2(12)	19.2(13)	19.7(11)	-0.9(10)	1.9(9)	-0.5(10)
C5	18.5(11)	19.6(13)	14.8(11)	0.5(9)	2.6(9)	-1.1(9)
C6	20.2(12)	29.9(16)	19.1(12)	-3.1(11)	5.5(9)	2.3(10)
C7	14.3(10)	23.1(15)	22.9(12)	-1.7(10)	3.6(9)	2.6(9)
C8	19.2(11)	25.9(15)	20.1(11)	1.0(10)	2.8(9)	-1.8(10)
C9	24.0(12)	19.5(15)	20.2(11)	-0.9(10)	1.7(9)	-0.5(10)

C10	18.4(11)	21.0(14)	16.0(11)	3.2(10)	2.7(9)	2.7(10)
C11	18.3(11)	21.4(14)	20.5(12)	-0.4(9)	-1.4(9)	0.1(10)
C12	38.4(15)	21.2(16)	34.2(15)	2.5(12)	6.5(12)	2.2(13)
C13	21.1(11)	22.6(15)	17.4(11)	4.2(9)	2.5(9)	1.5(10)
C14	29.1(13)	32.8(16)	19.6(12)	-0.6(11)	6.3(10)	9.6(12)
C15	34.3(15)	43.0(19)	17.0(12)	1.1(12)	2.1(10)	-4.6(14)

Table S4 Bond Lengths for 1.

Atom	Atom	Length/Å	Atom	Atom	Length/Å
O1	C3	1.228(3)	C5	C6	1.540(3)
O2	C7	1.413(3)	C6	C7	1.554(3)
O3	C8	1.219(3)	C7	C8	1.526(4)
O4	C13	1.429(3)	C7	C11	1.524(4)
C1	C2	1.338(4)	C8	C9	1.509(4)
C1	C5	1.522(3)	C9	C10	1.536(4)
C1	C10	1.514(3)	C10	C13	1.541(3)
C2	C3	1.460(4)	C10	C14	1.529(3)
C3	C4	1.517(3)	C11	C12	1.321(5)
C4	C5	1.545(3)	C11	C13	1.514(4)
C4	C15	1.517(4)			

Table S5 Bond Angles for 1.

Atom	Atom	Atom	Angle/˚	Atom	Atom	Atom	Angle/˚
C2	C1	C5	112.0(2)	C11	C7	C6	108.7(2)
C2	C1	C10	125.6(2)	C11	C7	C8	110.4(2)
C10	C1	C5	122.3(2)	O3	C8	C7	121.5(2)
C1	C2	C3	110.3(2)	O3	C8	C9	121.2(3)
O1	C3	C2	126.3(2)	C9	C8	C7	117.3(2)
O1	C3	C4	125.3(2)	C8	C9	C10	114.2(2)
C2	C3	C4	108.4(2)	C1	C10	C9	111.3(2)
C3	C4	C5	104.2(2)	C1	C10	C13	106.7(2)
C3	C4	C15	113.0(2)	C1	C10	C14	111.2(2)
C15	C4	C5	116.0(2)	C9	C10	C13	108.2(2)
C1	C5	C4	103.65(19)	C14	C10	C9	108.8(2)
C1	C5	C6	117.1(2)	C14	C10	C13	110.6(2)
C6	C5	C4	113.1(2)	C12	C11	C7	121.6(2)
C5	C6	C7	117.4(2)	C12	C11	C13	121.1(2)

O2	C7	C6	108.6(2)	C13	C11	C7	117.3(2)
O2	C7	C8	111.6(2)	O4	C13	C10	106.5(2)
O2	C7	C11	108.5(2)	O4	C13	C11	110.3(2)
C8	C7	C6	109.0(2)	C11	C13	C10	111.3(2)

Table S6 Torsion Angles for 1.

A	B	C	D	Angle/°	A	B	C	D	Angle/°
O1	C3	C4	C5	169.9(3)	C5	C6	C7	C8	-54.8(3)
O1	C3	C4	C15	43.1(4)	C5	C6	C7	C11	65.6(3)
O2	C7	C8	O3	11.0(4)	C6	C7	C8	O3	-109.0(3)
O2	C7	C8	C9	-169.2(2)	C6	C7	C8	C9	70.8(3)
O2	C7	C11	C12	-31.4(3)	C6	C7	C11	C12	86.5(3)
O2	C7	C11	C13	148.7(2)	C6	C7	C11	C13	-93.3(3)
O3	C8	C9	C10	-166.6(3)	C7	C8	C9	C10	13.6(3)
C1	C2	C3	O1	-175.7(3)	C7	C11	C13	O4	-90.1(3)
C1	C2	C3	C4	4.3(3)	C7	C11	C13	C10	27.9(3)
C1	C5	C6	C7	-27.5(3)	C8	C7	C11	C12	-154.0(3)
C1	C10	C13	O4	177.4(2)	C8	C7	C11	C13	26.2(3)
C1	C10	C13	C11	57.1(3)	C8	C9	C10	C1	-75.4(3)
C2	C1	C5	C4	-9.9(3)	C8	C9	C10	C13	41.5(3)
C2	C1	C5	C6	-135.2(2)	C8	C9	C10	C14	161.7(2)
C2	C1	C10	C9	-150.1(3)	C9	C10	C13	O4	57.5(2)
C2	C1	C10	C13	92.1(3)	C9	C10	C13	C11	-62.7(3)
C2	C1	C10	C14	-28.6(4)	C10	C1	C2	C3	-179.3(3)
C2	C3	C4	C5	-10.1(3)	C10	C1	C5	C4	173.0(2)
C2	C3	C4	C15	-136.9(3)	C10	C1	C5	C6	47.7(4)
C3	C4	C5	C1	11.6(3)	C11	C7	C8	O3	131.7(3)
C3	C4	C5	C6	139.4(2)	C11	C7	C8	C9	-48.5(3)
C4	C5	C6	C7	-148.0(2)	C12	C11	C13	O4	90.1(3)
C5	C1	C2	C3	3.7(3)	C12	C11	C13	C10	-151.9(3)
C5	C1	C10	C9	26.6(3)	C14	C10	C13	O4	-61.6(3)
C5	C1	C10	C13	-91.2(3)	C14	C10	C13	C11	178.2(2)
C5	C1	C10	C14	148.1(3)	C15	C4	C5	C1	136.5(2)
C5	C6	C7	O2	-176.5(2)	C15	C4	C5	C6	-95.6(3)

Table S7 Hydrogen Atom Coordinates (Å×10^4) and Isotropic Displacement Parameters (Å^2×10^3) for 1.

Atom	*x*	*y*	*z*	U(eq)
H2	11551.18	4686.43	6346.34	43
H4	8472.83	4474.88	10963.3	44
H2A	3956.52	5918.48	7524.52	27
H4A	6118.49	4270.21	4377.33	25
H5	7343.86	7237.15	5389.64	21
H6A	9347.2	5650.76	4993.43	27
H6B	8446.04	4032.08	5329.85	27
H9A	8228.24	8509.89	7612.63	25
H9B	8799.32	8130.95	9192.16	25
H12A	9500(50)	1770(60)	7560(50)	45(11)
H12B	8040(50)	1670(60)	8840(50)	55(13)
H13	6669.72	4226.01	9334.07	24
H14A	5528.51	8413.51	8984.71	41
H14B	6493.81	7749.3	10326.75	41
H14C	5078.3	6728.12	9673.33	41
H15A	5582.66	7440.24	3239.66	47
H15B	4978.21	5812	2506.96	47
H15C	6785.17	6171.83	2653.13	47

ECD calculation for compounds 2−5

Conformation search using molecular mechanics calculations was performed in CONFLEX version 7.0 with MMFF force field with an energy window for acceptable conformers (ewindow) of 5 kcal/mol above the ground state, a maximum number of conformations per molecule (maxconfs) of 100, and an RMSD cutoff (rmsd) of 0.5Å. Then the predominant conformers were optimized at B3LYP/6-311g(d,p) level in Gaussian 09 (Frisch et al. 2009) [1]. The optimized conformation geometries and thermodynamic parameters of all selected conformations were provided. The optimized conformers of **2**–**5** were used for the ECD calculation, which were performed with Gaussian 09 (B3LYP/6-311g(d,p)). The solvent effects were taken into account by the polarizable-conductor calculation model (PCM, methanol as the solvent). Percentages for each conformation are shown in Table S1.

Selected conformation of 1 and their percentage

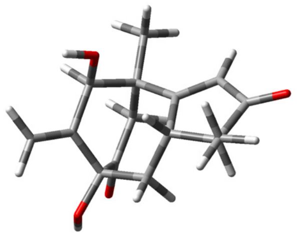

1-1 (47.06%)

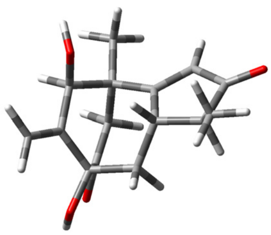

1-2 (52.94%)

Figure S39. The lowest energy conformers of **1** (the relative populations are in parentheses).

Selected conformation of 2 and their percentage

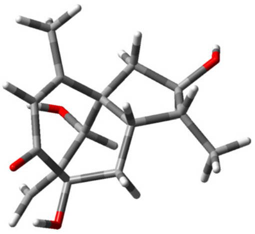

2-1 (24.27%)

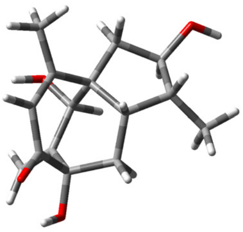

2-2 (17.79%)

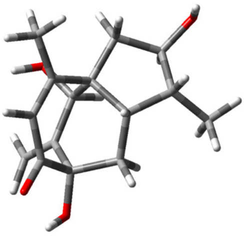

2-3 (20.73%)

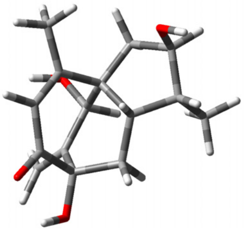

2-4 (21.10%)

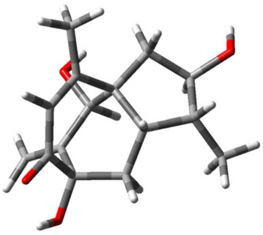

2-5 (5.92%)

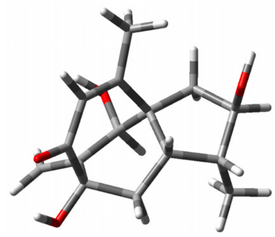

2-6 (10.19%)

Figure S40. The lowest energy conformers of **2** (the relative populations are in parentheses).

Selected conformation of 3 and their percentage

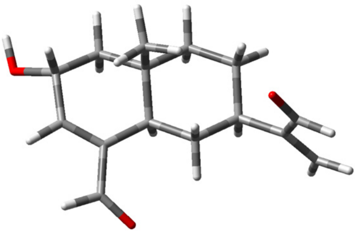

3-1 (9.92%)

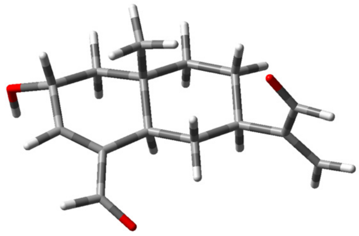

3-2 (20.19%)

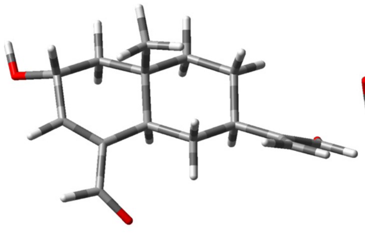

3-3 (20.02%)

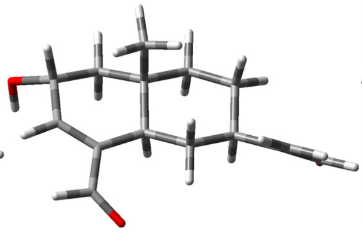

3-4 (40.68%)

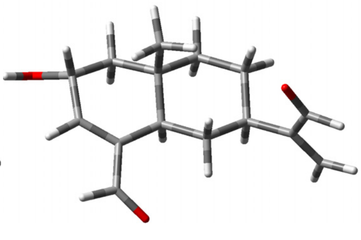

3-5 (9.20%)

Figure S41. The lowest energy conformers of **3** (the relative populations are in parentheses).

Selected conformation of 4 and their percentage

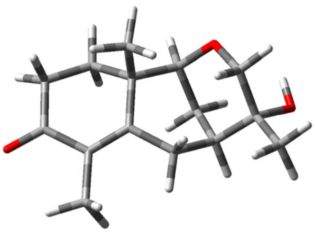

4-1 (52.91%)

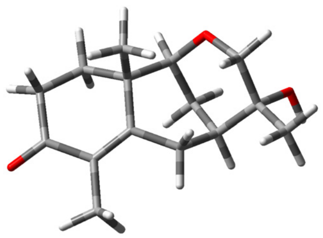

4-2 (47.09%)

Figure S42. The lowest energy conformers of **4** (the relative populations are in parentheses).

Selected conformation of 5 and their percentage

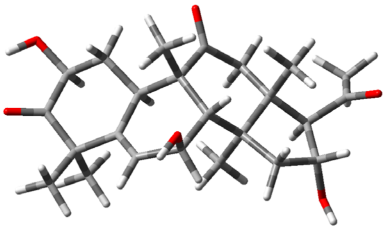

5-1 (24.79%)

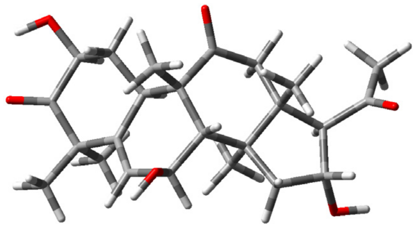

5-2 (20.55%)

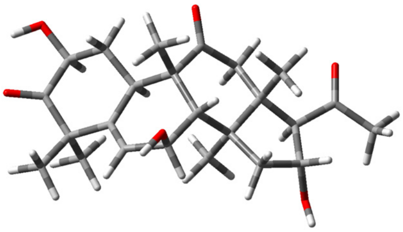

5-3 (7.96%)

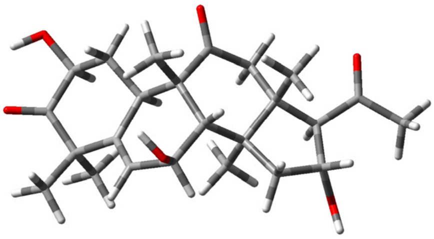

5-4 (9.86%)

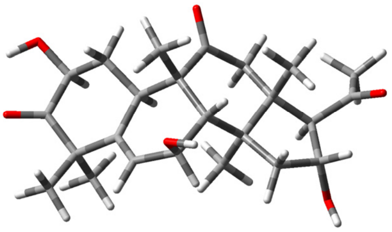

5-5 (20.10%)

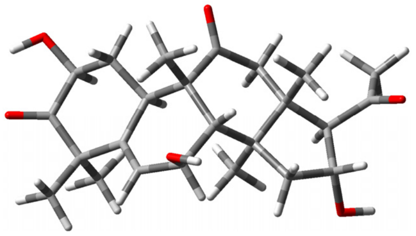

5-6 (16.75%)

Figure S43. The lowest energy conformers of **5** (the relative populations are in parentheses).

Table S8. Extracted heats and weighting factors of the optimized conformers of **2–5** at B3LYP/6-311g(d,p) level

		B3LYP/6-31+G(d)	
	Conformer	Extracted heats	Boltzmann-calculated contribution (%)
**2**	1	-884.45037	24.27%
	2	-884.45008	17.79%
	3	-884.45022	20.72%
	4	-884.45024	21.10%
	5	-884.44904	5.92%

	6	-884.44955	10.19%
**3**	1	-809.20954	9.91%
	2	-809.21021	20.19%
	3	-809.21021	20.02%
	4	-809.21087	40.68%
	5	-809.20947	9.20%

**4**	1	-810.41392	52.91%
	2	-810.41381	47.08%
**5**	1	-1386.2244	24.79%
	2	-1386.22422	20.55%
	3	-1386.22333	7.96%
	4	-1386.22353	9.86%
	5	-1386.2242	20.10%
	6	-1386.22403	16.75%

Table S9. The Cartesian coordinates of the lowest energy conformers for **2–5**

**2-1**	X axis(Å)	Y axis(Å)	Z axis(Å)	**2-2**	X axis(Å)	Y axis(Å)	Z axis(Å)
C	-0.6046	0.6655	-0.085	C	-0.6008	0.6746	-0.0758
C	-2.0891	1.0909	-0.0344	C	-2.0813	1.1109	-0.0226
C	-2.8379	-0.2009	0.2617	C	-2.8444	-0.1753	0.2616
C	-2.1532	-1.2266	-0.6473	C	-2.1641	-1.2056	-0.6463
C	-0.6893	-0.7296	-0.7983	C	-0.6983	-0.7126	-0.8024
C	0.4351	-1.6823	-0.3687	C	0.4239	-1.6764	-0.3918
C	1.6828	-0.9264	0.1064	C	1.6765	-0.935	0.092
C	2.222	4.00E-04	-1.0121	C	2.2215	0.0014	-1.015
C	1.5343	1.2873	-1.3199	C	1.5412	1.2951	-1.3086
C	0.3015	1.6263	-0.8899	C	0.3114	1.6369	-0.8727
C	-0.2126	3.0138	-1.1835	C	-0.1938	3.0314	-1.1502
O	3.2346	-0.2987	-1.6515	O	3.2334	-0.2963	-1.6562
O	2.6755	-1.9085	0.4191	O	2.6614	-1.9297	0.3903
C	-0.0228	0.4861	1.3598	C	-0.0179	0.4748	1.3659
C	1.3757	-0.1063	1.3527	C	1.3759	-0.1299	1.3491
C	2.2438	0.0651	2.3683	C	2.2464	0.0221	2.3658
O	-0.0489	1.7288	2.0589	O	-0.0276	1.7071	2.0822
C	-2.3703	-2.6541	-0.1625	C	-2.3811	-2.6314	-0.1556
O	-4.211	-0.0818	-0.0635	O	-4.2119	-0.0137	-0.0703
H	-0.5558	-0.5683	-1.8798	H	-0.5718	-0.5397	-1.883
H	-2.2997	1.8553	0.7211	H	-2.2856	1.8711	0.739
H	-2.4386	1.4912	-0.9944	H	-2.4257	1.523	-0.9795
H	-2.758	-0.48	1.3176	H	-2.7758	-0.4463	1.3205
H	-2.6215	-1.17	-1.6413	H	-2.6348	-1.1514	-1.6392
H	0.6861	-2.3505	-1.2031	H	0.6692	-2.3321	-1.2378

H	0.1146	-2.3455	0.4433	H	0.104	-2.3517	0.4103
H	2.1385	1.9686	-1.9132	H	2.1485	1.9794	-1.8952
H	0.5646	3.6583	-1.6093	H	0.5877	3.6757	-1.5683
H	-1.0346	2.9797	-1.9044	H	-1.0158	3.0113	-1.8717
H	-0.5603	3.4975	-0.2652	H	-0.5384	3.5068	-0.2264
H	3.2971	-1.9119	-0.3372	H	3.2981	-1.9081	-0.3532
H	-0.6566	-0.2026	1.93	H	-0.6568	-0.2154	1.9288
H	1.9795	0.6313	3.2569	H	1.9867	0.5806	3.2606
H	3.2334	-0.3841	2.3497	H	3.2328	-0.4334	2.3409
H	0.7251	2.2397	1.7642	H	0.7268	2.2316	1.7629
H	-1.8508	-3.3702	-0.8071	H	-1.8619	-3.3501	-0.7975
H	-3.4365	-2.9053	-0.181	H	-3.4467	-2.8847	-0.1729
H	-2.0223	-2.7918	0.8661	H	-2.0325	-2.7643	0.8736
H	-4.591	0.6038	0.5131	H	-4.6682	-0.8482	0.1333
**2-3**	X axis(Å)	Y axis(Å)	Z axis(Å)	**2-4**	X axis(Å)	Y axis(Å)	Z axis(Å)
C	-0.6039	0.6703	-0.075	C	-0.7184	0.4027	0.3642
C	-2.0873	1.1011	-0.0162	C	-2.1976	0.4868	0.861
C	-2.839	-0.1899	0.2807	C	-3.03	-0.3343	-0.1218
C	-2.1628	-1.2191	-0.6311	C	-2.1021	-1.4738	-0.5557
C	-0.698	-0.7264	-0.786	C	-0.7575	-0.7358	-0.7156
C	0.4239	-1.6832	-0.3603	C	0.5219	-1.5742	-0.7536
C	1.6788	-0.9315	0.1015	C	1.7439	-0.7576	-0.308
C	2.2111	-0.0096	-1.0247	C	1.9205	0.5086	-1.1842
C	1.5274	1.2804	-1.3286	C	1.0095	1.6792	-1.0153
C	0.2996	1.6258	-0.8895	C	-0.1448	1.6771	-0.3143
C	-0.2078	3.0176	-1.1749	C	-0.9136	2.9679	-0.1964
O	3.2141	-0.3164	-1.6754	O	2.8268	0.5778	-2.0199
O	2.6699	-1.9176	0.4059	O	2.889	-1.6021	-0.4637
C	-0.0094	0.4952	1.3658	C	0.2028	0.0017	1.566
C	1.385	-0.1086	1.3486	C	1.6215	-0.3362	1.1486
C	2.2602	0.056	2.3593	C	2.6613	-0.281	2.0032
O	-0.0144	1.7433	2.0553	O	0.1952	1.0276	2.5581
C	-2.3837	-2.6451	-0.1441	C	-2.1243	-2.6302	0.4399
O	-4.2216	-0.0661	-8.00E-04	O	-3.3536	0.4858	-1.2402
H	-0.5677	-0.567	-1.8682	H	-0.8284	-0.2581	-1.7069
H	-2.2915	1.8639	0.7428	H	-2.2896	0.0292	1.854
H	-2.4379	1.5066	-0.9734	H	-2.5825	1.5041	0.9562
H	-2.7414	-0.4642	1.3361	H	-3.9657	-0.6804	0.3282
H	-2.6325	-1.1628	-1.6242	H	-2.41	-1.8661	-1.5334
H	0.665	-2.3569	-1.1933	H	0.6721	-1.9654	-1.7686
H	0.1056	-2.3405	0.4574	H	0.4464	-2.4614	-0.1146
H	2.1312	1.9589	-1.9255	H	1.3682	2.5763	-1.5133
H	0.5704	3.6584	-1.6045	H	-0.3806	3.8093	-0.6534

H	-1.0353	2.9909	-1.8899	H	-1.8813	2.8906	-0.7008
H	-0.5458	3.5008	-0.2528	H	-1.0705	3.2258	0.8558
H	3.2923	-1.9159	-0.3497	H	3.3691	-1.2588	-1.2449
H	-0.6438	-0.1836	1.9471	H	-0.2054	-0.8933	2.0488
H	2.0045	0.6241	3.2493	H	2.5286	9.00E-04	3.0439
H	3.2461	-0.4007	2.336	H	3.6684	-0.5421	1.6895
H	0.7643	2.2428	1.7547	H	0.7665	1.7438	2.2307
H	-1.8688	-3.3639	-0.7895	H	-1.5163	-3.4669	0.0824
H	-3.4509	-2.8922	-0.159	H	-3.1462	-3.007	0.5592
H	-2.0328	-2.7832	0.8836	H	-1.7629	-2.3437	1.4299
H	-4.3295	0.1945	-0.9312	H	-3.9028	-0.0455	-1.8428
**2-5**	X axis(Å)	Y axis(Å)	Z axis(Å)	**2-6**	X axis(Å)	Y axis(Å)	Z axis(Å)
C	-0.6012	0.6612	-0.1052	C	-0.7223	0.3935	0.3588
C	-2.0859	1.0892	-0.0813	C	-2.2049	0.4681	0.8492
C	-2.8386	-0.1914	0.2492	C	-3.0275	-0.3575	-0.1382
C	-2.1486	-1.246	-0.6215	C	-2.0892	-1.4878	-0.5732
C	-0.6838	-0.7537	-0.7791	C	-0.7493	-0.7402	-0.7257
C	0.4363	-1.6917	-0.3108	C	0.5353	-1.5709	-0.7625
C	1.6841	-0.9179	0.1349	C	1.7497	-0.7492	-0.3071
C	2.2175	-0.0357	-1.0224	C	1.9223	0.5219	-1.1769
C	1.5374	1.2465	-1.3615	C	1.0044	1.687	-1.0052
C	0.3088	1.6001	-0.931	C	-0.1527	1.6757	-0.3086
C	-0.1876	2.9937	-1.2242	C	-0.9226	2.9643	-0.1809
O	3.2173	-0.37	-1.6648	O	2.8314	0.6015	-2.0087
O	2.6744	-1.8915	0.4826	O	2.9021	-1.5841	-0.4602
C	-0.0275	0.5148	1.3488	C	0.195	-0.0065	1.5634
C	1.3861	-0.0459	1.3515	C	1.6173	-0.336	1.1516
C	2.2982	0.2003	2.3124	C	2.6518	-0.2834	2.013
O	-0.0967	1.7686	2.0208	O	0.1748	1.0207	2.5549
C	-2.3686	-2.657	-0.0919	C	-2.1064	-2.6491	0.4171
O	-4.21	-0.0843	-0.0865	O	-3.3544	0.4433	-1.2694
H	-0.5432	-0.625	-1.864	H	-0.8194	-0.2586	-1.7151
H	-2.3032	1.8778	0.647	H	-2.2987	0.0082	1.8411
H	-2.4255	1.4589	-1.0572	H	-2.5958	1.483	0.9447
H	-2.7655	-0.4356	1.3143	H	-3.961	-0.725	0.2996
H	-2.6105	-1.2218	-1.6198	H	-2.3921	-1.8773	-1.5536
H	0.687	-2.3929	-1.1179	H	0.6924	-1.9557	-1.7789
H	0.1137	-2.3213	0.5267	H	0.4627	-2.462	-0.1285
H	2.1468	1.9167	-1.9611	H	1.3624	2.5884	-1.4957
H	0.5944	3.6256	-1.6598	H	-0.3894	3.8108	-0.628
H	-1.0171	2.9695	-1.937	H	-1.8886	2.8902	-0.6894
H	-0.5198	3.4856	-0.3046	H	-1.0839	3.2117	0.8731
H	3.297	-1.9172	-0.2728	H	3.3647	-1.2559	-1.2583

H	-0.6515	-0.1842	1.9186	H	-0.2113	-0.9037	2.0435
H	2.0962	0.8247	3.1742	H	2.5128	-0.0105	3.0551
H	3.2962	-0.2288	2.2581	H	3.6613	-0.54	1.7029
H	0.1194	1.6072	2.9539	H	0.7872	1.7154	2.2567
H	-1.8449	-3.3935	-0.7095	H	-1.4913	-3.4798	0.0579
H	-3.4346	-2.909	-0.1089	H	-3.1259	-3.034	0.5311
H	-2.0269	-2.7612	0.9429	H	-1.7506	-2.3643	1.4097
H	-4.5818	0.658	0.421	H	-3.9915	1.1175	-0.9756
**3-1**	X axis(Å)	Y axis(Å)	Z axis(Å)	**3-2**	X axis(Å)	Y axis(Å)	Z axis(Å)
C	3.2901	-0.6478	0.3092	C	3.2921	-0.6632	0.3092
C	2.8738	0.7804	0.4809	C	2.8778	0.7661	0.4897
C	1.6489	1.2461	0.1657	C	1.6533	1.2325	0.1746
C	0.55	0.3543	-0.3885	C	0.5552	0.3426	-0.3854
C	0.8146	-1.16	-0.0693	C	0.8123	-1.171	-0.0559
C	2.2718	-1.4856	-0.4742	C	2.2674	-1.507	-0.4604
C	-0.9037	0.7155	-0.0125	C	-0.8987	0.7135	-0.0186
C	-1.914	-0.1681	-0.7788	C	-1.9108	-0.172	-0.78
C	-1.6297	-1.6751	-0.6583	C	-1.6347	-1.6787	-0.6415
C	-0.1589	-2.0152	-0.9138	C	-0.1657	-2.0282	-0.8937
C	-3.3494	0.176	-0.4356	C	-3.346	0.1825	-0.4468
C	-3.859	-0.0273	0.9495	C	-3.8614	-7.00E-04	0.939
O	-3.1822	-0.4989	1.8546	O	-3.1873	-0.4571	1.8538
C	-4.2173	0.6787	-1.3296	C	-4.209	0.6759	-1.3506
O	4.5309	-0.6424	-0.3843	O	4.5307	-0.6742	-0.389
C	1.4832	2.7204	0.272	C	1.4796	2.7041	0.3011
O	0.5486	3.3379	-0.2212	O	0.5769	3.3283	-0.2403
C	0.6255	-1.5084	1.4317	C	0.6224	-1.5081	1.4474
H	3.4683	-1.0852	1.2979	H	3.4761	-1.1037	1.2951
H	3.6538	1.443	0.8493	H	3.652	1.4237	0.8798
H	0.6221	0.4792	-1.4809	H	0.6346	0.4622	-1.4778
H	2.4079	-1.3163	-1.552	H	2.3971	-1.3539	-1.5413
H	2.4912	-2.5507	-0.3224	H	2.4843	-2.5708	-0.2959
H	-1.1263	1.7541	-0.2752	H	-1.1148	1.751	-0.2913
H	-1.0412	0.6477	1.0717	H	-1.0401	0.6558	1.0657
H	-1.7663	0.0732	-1.843	H	-1.7574	0.0573	-1.8461
H	-2.2481	-2.2223	-1.3818	H	-2.2558	-2.2313	-1.3585
H	-1.922	-2.0578	0.3248	H	-1.9286	-2.0478	0.3463
H	0.0523	-1.8661	-1.9811	H	0.0464	-1.8889	-1.9621
H	0.0019	-3.0824	-0.7158	H	-0.0103	-3.0945	-0.6869
H	-4.9065	0.2724	1.1229	H	-4.9099	0.3005	1.1037
H	-5.2432	0.9292	-1.0769	H	-5.235	0.9332	-1.1047
H	-3.9169	0.863	-2.3574	H	-3.9049	0.8447	-2.38
H	4.7976	-1.5694	-0.5056	H	4.3783	-0.2779	-1.2639

H	2.2885	3.255	0.8045	H	2.2533	3.2289	0.8876
H	0.903	-2.5522	1.62	H	0.8882	-2.5539	1.6414
H	1.2368	-0.8834	2.0894	H	1.2426	-0.8869	2.1003
H	-0.4068	-1.3888	1.7695	H	-0.4078	-1.3744	1.7863
**3-3**	X axis(Å)	Y axis(Å)	Z axis(Å)	**3-4**	X axis(Å)	Y axis(Å)	Z axis(Å)
C	3.3895	-0.7525	0.0866	C	3.3884	-0.7647	0.0864
C	3.0684	0.7099	0.0598	C	3.0716	0.7002	0.0759
C	1.8237	1.2059	-0.0924	C	1.8273	1.1977	-0.0732
C	0.6067	0.3076	-0.2638	C	0.6096	0.3017	-0.2545
C	0.8692	-1.1242	0.3258	C	0.8678	-1.1343	0.3279
C	2.1973	-1.6465	-0.2733	C	2.1949	-1.6583	-0.272
C	-0.7535	0.8386	0.2409	C	-0.7494	0.8333	0.2539
C	-1.9148	-0.1061	-0.1575	C	-1.9124	-0.1053	-0.1528
C	-1.6629	-1.5464	0.3183	C	-1.666	-1.5492	0.3143
C	-0.2842	-2.0609	-0.1003	C	-0.288	-2.0655	-0.1046
C	-3.2897	0.3676	0.2835	C	-3.2867	0.3689	0.2885
C	-4.4604	-0.1333	-0.4968	C	-4.4555	-0.1127	-0.5066
O	-4.3516	-0.914	-1.4346	O	-4.344	-0.8742	-1.4598
C	-3.5664	1.2238	1.2809	C	-3.5639	1.2088	1.2995
O	4.4454	-0.967	-0.8393	O	4.4462	-0.9903	-0.8352
C	1.745	2.6897	-0.176	C	1.7453	2.6824	-0.1341
O	0.7207	3.3124	-0.4198	O	0.737	3.3011	-0.4447
C	0.9762	-1.1321	1.8745	C	0.9733	-1.1512	1.8766
H	3.7685	-1.0094	1.0822	H	3.7666	-1.0319	1.0793
H	3.9317	1.3649	0.1566	H	3.9338	1.3533	0.1963
H	0.4993	0.2	-1.3552	H	0.5038	0.2014	-1.3466
H	2.111	-1.7197	-1.3669	H	2.1035	-1.7339	-1.3649
H	2.4024	-2.6705	0.066	H	2.4004	-2.6822	0.0674
H	-0.9765	1.8119	-0.2051	H	-0.9691	1.8114	-0.1831
H	-0.7131	0.993	1.3247	H	-0.7094	0.9787	1.339
H	-1.9178	-0.1185	-1.2576	H	-1.9125	-0.1105	-1.253
H	-2.4204	-2.2219	-0.0981	H	-2.4252	-2.2193	-0.108
H	-1.7744	-1.6107	1.4072	H	-1.7796	-1.6201	1.4026
H	-0.276	-2.1804	-1.192	H	-0.2787	-2.1793	-1.1969
H	-0.1335	-3.0638	0.3187	H	-0.1409	-3.071	0.3093
H	-5.448	0.2409	-0.178	H	-5.4435	0.258	-0.1849
H	-4.5799	1.5297	1.5242	H	-4.5771	1.5158	1.543
H	-2.7853	1.6584	1.8961	H	-2.7838	1.6269	1.9273
H	4.6275	-1.9219	-0.8567	H	4.1163	-0.7679	-1.7228
H	2.6947	3.2277	-0.0132	H	2.679	3.2223	0.1001
H	1.2274	-2.1365	2.2352	H	1.2209	-2.1585	2.2319
H	1.7453	-0.4505	2.2491	H	1.7445	-0.4744	2.2555
H	0.0436	-0.8411	2.3649	H	0.0412	-0.8602	2.3679

**3-5**	X axis(Å)	Y axis(Å)	Z axis(Å)				
C	3.2906	-0.6594	0.2986				
C	2.8782	0.7728	0.4715				
C	1.6518	1.2381	0.1582				
C	0.5506	0.3463	-0.3939				
C	0.8115	-1.1662	-0.066				
C	2.2655	-1.4967	-0.4755				
C	-0.9024	0.7135	-0.0204				
C	-1.9158	-0.1739	-0.778				
C	-1.6356	-1.6801	-0.6434				
C	-0.1667	-2.0254	-0.9012				
C	-3.3503	0.1764	-0.4373				
C	-3.8587	-0.0091	0.9507				
O	-3.18	-0.4644	1.8627				
C	-4.2188	0.6682	-1.3368				
O	4.5173	-0.6875	-0.4195				
C	1.4811	2.712	0.2706				
O	0.5511	3.3316	-0.228				
C	0.627	-1.5045	1.4378				
H	3.4713	-1.1064	1.2827				
H	3.6491	1.4415	0.8457				
H	0.6237	0.4658	-1.4868				
H	2.396	-1.3333	-1.555				
H	2.4832	-2.5618	-0.3205				
H	-1.1231	1.7501	-0.2922				
H	-1.0389	0.6553	1.0645				
H	-1.7681	0.0573	-1.8445				
H	-2.2578	-2.2329	-1.3593				
H	-1.9255	-2.0518	0.3447				
H	0.0414	-1.8846	-1.9702				
H	-0.0077	-3.0915	-0.6959				
H	-4.9072	0.2895	1.1201				
H	-5.2443	0.9227	-1.0863				
H	-3.9194	0.8383	-2.3674				
H	5.2074	-0.3331	0.1653				
H	2.2805	3.2453	0.8129				
H	0.8997	-2.5487	1.6311				
H	1.2446	-0.8793	2.0893				
H	-0.4032	-1.377	1.7792				
**4-1**	X axis(Å)	Y axis(Å)	Z axis(Å)	**4-2**	X axis(Å)	Y axis(Å)	Z axis(Å)
C	3.1226	-1.3207	-0.2147	C	3.1395	-1.3057	-0.1813
C	3.3033	0.1658	-0.0502	C	3.3075	0.1867	-0.0738
C	2.093	1.0539	0.0455	C	2.0904	1.0602	0.0666

C	0.8465	0.5217	0.1153	C	0.8532	0.5118	0.1595
C	0.5995	-1.0037	0.1635	C	0.6116	-1.0154	0.1686
C	1.7216	-1.6825	-0.6735	C	1.7485	-1.6845	-0.6532
C	-0.3954	1.4085	0.0792	C	-0.4062	1.3662	0.2086
C	-1.5841	0.8896	-0.7782	C	-1.5621	0.9032	-0.7212
C	-1.1789	-0.3692	-1.5495	C	-1.1532	-0.325	-1.5397
C	-0.7653	-1.4095	-0.511	C	-0.7404	-1.4046	-0.5437
O	-1.8556	-1.689	0.3856	O	-1.828	-1.7401	0.3288
C	-2.8289	0.5571	0.0799	C	-2.84	0.5379	0.0687
C	-2.4822	-0.5833	1.0477	C	-2.5499	-0.68	0.9669
C	-3.3934	1.7682	0.8242	C	-3.4073	1.7011	0.8887
O	-3.8844	0.1199	-0.7957	O	-3.873	0.1757	-0.8652
C	0.6887	-1.5151	1.6174	C	0.6627	-1.5436	1.6175
C	2.4313	2.5218	0.0779	C	2.4159	2.5303	0.1185
O	4.435	0.6464	0.0166	O	4.4336	0.6846	-0.0791
H	3.3719	-1.8069	0.7335	H	3.3751	-1.7536	0.7888
H	3.8409	-1.6704	-0.9652	H	3.8715	-1.6801	-0.9063
H	1.6143	-2.7746	-0.6396	H	1.6508	-2.7774	-0.6169
H	1.6195	-1.3971	-1.73	H	1.6542	-1.4022	-1.7114
H	-0.1486	2.3897	-0.3412	H	-0.1945	2.4002	-0.0811
H	-0.6884	1.6007	1.1157	H	-0.7164	1.4191	1.2567
H	-1.863	1.6573	-1.5124	H	-1.802	1.71	-1.4267
H	-2.0134	-0.7442	-2.1521	H	-1.9878	-0.6786	-2.1558
H	-0.3672	-0.1541	-2.2544	H	-0.3389	-0.0822	-2.2326
H	-0.6171	-2.3595	-1.0393	H	-0.5729	-2.3275	-1.1126
H	-1.8537	-0.2349	1.8689	H	-2.0067	-0.3806	1.8664
H	-3.3977	-0.9809	1.5008	H	-3.4935	-1.1206	1.3088
H	-4.343	1.5125	1.309	H	-4.3784	1.4293	1.3191
H	-3.6144	2.5825	0.1247	H	-3.586	2.5733	0.2496
H	-2.7185	2.1497	1.5948	H	-2.7519	2.0057	1.7089
H	-3.7876	-0.8447	-0.9085	H	-4.0251	0.9295	-1.46
H	-0.0205	-1.0202	2.2827	H	-0.0733	-1.0604	2.2634
H	0.4994	-2.5935	1.6635	H	0.4813	-2.6239	1.6466
H	1.6726	-1.3353	2.0626	H	1.632	-1.3597	2.0924
H	3.2746	2.7159	0.75	H	3.2851	2.7181	0.7587
H	2.7002	2.8694	-0.9247	H	2.6395	2.9026	-0.8863
H	1.6144	3.1437	0.4525	H	1.6115	3.1364	0.5413
**5-1**	X axis(Å)	Y axis(Å)	Z axis(Å)	**5-2**	X axis(Å)	Y axis(Å)	Z axis(Å)
C	-3.8875	1.4428	-0.7715	C	-3.8838	1.4433	-0.7722
C	-4.4401	0.0222	-0.7693	C	-4.4406	0.0245	-0.7758
C	-3.42	-1.1217	-0.9149	C	-3.4233	-1.1219	-0.9233
C	-2.2254	-0.8632	0.0361	C	-2.2285	-0.8701	0.0293
C	-1.6386	0.5546	0.0381	C	-1.6407	0.5472	0.042

C	-2.7885	1.564	0.2704	C	-2.7894	1.5576	0.275
C	-1.6832	-1.8349	0.7952	C	-1.6856	-1.8482	0.7799
C	-0.4434	-1.7179	1.6181	C	-0.4441	-1.7393	1.6018
C	0.4498	-0.5041	1.2847	C	0.4456	-0.5195	1.2806
C	-0.4038	0.8002	1.0023	C	-0.4118	0.7843	1.0145
C	1.5467	-0.7892	0.2097	C	1.5419	-0.791	0.2009
C	2.4606	0.4794	0.0634	C	2.4578	0.4796	0.068
C	1.6281	1.5964	-0.5519	C	1.6168	1.6013	-0.5292
C	0.4366	1.9352	0.3374	C	0.4305	1.9279	0.3711
C	2.6021	-1.8525	0.6063	C	2.5908	-1.8614	0.5865
C	3.8576	-1.4932	-0.2245	C	3.8603	-1.4824	-0.212
C	3.6152	-0.0782	-0.808	C	3.6123	-0.07	-0.8075
C	3.0525	1.0218	1.406	C	3.0608	1.001	1.4138
C	0.9175	-1.2872	-1.1272	C	0.9158	-1.2769	-1.142
O	4.0051	-2.417	-1.3003	O	4.0549	-2.4202	-1.266
C	4.9003	0.7447	-0.8214	C	4.8953	0.758	-0.8487
O	5.7649	0.6339	0.0481	O	5.9305	0.3936	-0.2909
C	5.0592	1.7397	-1.9424	C	4.8567	2.0626	-1.6027
O	0.0932	3.1186	0.4217	O	0.0974	3.1118	0.4836
C	-0.8622	1.3487	2.3858	C	-0.8782	1.3121	2.4032
C	-4.1632	-2.4518	-0.6508	C	-4.1708	-2.4505	-0.6641
C	-2.9323	-1.153	-2.3759	C	-2.9337	-1.1497	-2.3837
O	-5.6615	-0.1584	-0.7352	O	-5.6627	-0.1521	-0.7448
O	-4.9117	2.385	-0.4662	O	-4.9059	2.3886	-0.4696
O	-0.8346	-1.6519	2.985	O	-0.8362	-1.6873	2.9691
H	-1.2844	0.7427	-0.983	H	-1.2813	0.7411	-0.9761
H	1.0055	-0.3215	2.2145	H	1.0026	-0.3441	2.2111
H	3.3065	-0.1609	-1.8578	H	3.3036	-0.1624	-1.8565
H	-3.5171	1.6746	-1.7746	H	-3.508	1.6764	-1.7731
H	-3.2361	1.4185	1.2628	H	-3.2417	1.4079	1.2646
H	-2.4146	2.5939	0.2527	H	-2.4129	2.5868	0.2643
H	-2.1051	-2.8365	0.7966	H	-2.1074	-2.8498	0.7733
H	0.1358	-2.641	1.5131	H	0.1367	-2.6602	1.4868
H	2.2351	2.5035	-0.6569	H	2.2147	2.5139	-0.6274
H	1.2584	1.3518	-1.5517	H	1.2424	1.3679	-1.5299
H	2.2606	-2.8763	0.4177	H	2.2554	-2.8817	0.3696
H	2.8346	-1.7799	1.675	H	2.8056	-1.8145	1.6607
H	4.7561	-1.5651	0.3966	H	4.7276	-1.5458	0.4535
H	3.6746	0.285	1.9228	H	2.3008	1.3298	2.1272
H	3.6778	1.904	1.2239	H	3.6762	0.2521	1.921
H	2.2878	1.3498	2.1145	H	3.6991	1.875	1.2403
H	0.2301	-0.571	-1.5726	H	0.2385	-0.5527	-1.5898
H	1.659	-1.5039	-1.9003	H	1.6621	-1.5002	-1.9092

H	0.3751	-2.2269	-0.981	H	0.3631	-2.2117	-1.0036
H	4.2929	-3.2636	-0.9141	H	4.9907	-2.3513	-1.5321
H	5.017	1.2203	-2.9029	H	4.4972	2.8585	-0.948
H	4.2724	2.4941	-1.886	H	5.8641	2.3093	-1.9501
H	6.03	2.2362	-1.8573	H	4.2096	1.9736	-2.4788
H	-0.0141	1.4439	3.0738	H	-0.0336	1.3989	3.0967
H	-1.3004	2.3505	2.3202	H	-1.3179	2.314	2.3495
H	-1.6073	0.7012	2.8564	H	-1.6251	0.6573	2.8608
H	-3.5237	-3.3218	-0.8382	H	-3.5343	-3.322	-0.8551
H	-5.0354	-2.5591	-1.3075	H	-5.0435	-2.5525	-1.3209
H	-4.5308	-2.5058	0.3808	H	-4.5381	-2.5072	0.3674
H	-3.7716	-1.3	-3.0663	H	-3.7727	-1.2896	-3.0759
H	-2.4404	-0.2225	-2.677	H	-2.4366	-0.2204	-2.6799
H	-2.2185	-1.9693	-2.5371	H	-2.2237	-1.9688	-2.5472
H	-5.7622	1.936	-0.6512	H	-5.7574	1.9394	-0.6498
H	-1.5368	-2.3072	3.1342	H	-1.4957	-2.3848	3.1218
**5-3**	X axis(Å)	Y axis(Å)	Z axis(Å)	**5-4**	X axis(Å)	Y axis(Å)	Z axis(Å)
C	-3.8827	1.3798	-0.8906	C	-3.8836	1.383	-0.8893
C	-4.4279	-0.0425	-0.8355	C	-4.4335	-0.0372	-0.8302
C	-3.399	-1.1856	-0.9069	C	-3.4086	-1.1837	-0.9033
C	-2.2254	-0.8781	0.0556	C	-2.2308	-0.8789	0.0555
C	-1.647	0.5426	0.0074	C	-1.6476	0.5397	0.005
C	-2.8076	1.554	0.1683	C	-2.8049	1.5546	0.1666
C	-1.6904	-1.8135	0.864	C	-1.6936	-1.8181	0.8577
C	-0.4606	-1.6557	1.6962	C	-0.4576	-1.6681	1.6818
C	0.4247	-0.4441	1.3322	C	0.4245	-0.4508	1.3274
C	-0.4398	0.8377	0.9912	C	-0.4392	0.8316	0.9889
C	1.541	-0.7541	0.2839	C	1.543	-0.7553	0.2785
C	2.4391	0.5233	0.0922	C	2.4409	0.523	0.0933
C	1.5968	1.6102	-0.5641	C	1.5996	1.6118	-0.5609
C	0.4002	1.9646	0.3119	C	0.4005	1.9612	0.3132
C	2.608	-1.7818	0.7396	C	2.6098	-1.7849	0.7309
C	3.8867	-1.4049	-0.05	C	3.8896	-1.4042	-0.0549
C	3.5962	-0.0612	-0.7614	C	3.5998	-0.0576	-0.7607
C	3.0185	1.1061	1.4269	C	3.0174	1.1007	1.4313
C	0.939	-1.3269	-1.0361	C	0.9422	-1.3221	-1.0449
O	4.1798	-2.4008	-1.0212	O	4.1847	-2.3952	-1.0305
C	4.843	0.8008	-0.9507	C	4.8461	0.8063	-0.9441
O	4.8204	2.0232	-0.805	O	4.8211	2.0283	-0.7952
C	6.0974	0.1226	-1.4446	C	6.1029	0.1312	-1.436
O	0.0467	3.1462	0.3747	O	0.044	3.1417	0.3794
C	-0.9398	1.4192	2.3467	C	-0.9373	1.4101	2.3465
C	-4.1409	-2.5068	-0.5982	C	-4.1536	-2.5022	-0.5909

C	-2.8804	-1.2799	-2.3545	C	-2.8943	-1.281	-2.3523
O	-5.6487	-0.2297	-0.8195	O	-5.6546	-0.2201	-0.8069
O	-4.9192	2.3273	-0.6489	O	-4.9157	2.3338	-0.6424
O	-0.8604	-1.554	3.0585	O	-0.8342	-1.6074	3.0537
H	-1.2683	0.6871	-1.0119	H	-1.2692	0.6825	-1.0146
H	0.9633	-0.2225	2.2633	H	0.9614	-0.2335	2.2603
H	3.2563	-0.2373	-1.7902	H	3.2617	-0.2295	-1.7909
H	-3.4923	1.569	-1.895	H	-3.4964	1.5706	-1.8953
H	-3.2769	1.4498	1.1559	H	-3.2723	1.453	1.1554
H	-2.4391	2.5845	0.1136	H	-2.4336	2.584	0.1102
H	-2.1057	-2.8172	0.8996	H	-2.1095	-2.8221	0.8992
H	0.1289	-2.5757	1.6244	H	0.133	-2.5846	1.5831
H	2.1927	2.52	-0.7037	H	2.1949	2.5227	-0.695
H	1.2302	1.3246	-1.5541	H	1.2359	1.33	-1.5531
H	2.2993	-2.818	0.5616	H	2.3018	-2.8202	0.5475
H	2.7995	-1.684	1.8146	H	2.7996	-1.6919	1.8066
H	4.7419	-1.3414	0.6317	H	4.7436	-1.3437	0.6284
H	3.6741	0.404	1.9498	H	2.2406	1.401	2.1388
H	3.6042	2.0125	1.2454	H	3.6721	0.3966	1.9526
H	2.2431	1.4094	2.1349	H	3.6034	2.0079	1.2545
H	0.2805	-0.6278	-1.5482	H	0.2845	-0.6206	-1.5546
H	1.699	-1.6165	-1.7671	H	1.7031	-1.6087	-1.7761
H	0.3742	-2.2451	-0.8468	H	0.3764	-2.2407	-0.8606
H	4.3322	-3.2399	-0.5504	H	4.3285	-3.2384	-0.5641
H	6.5111	-0.522	-0.6665	H	5.8833	-0.4455	-2.3378
H	5.8742	-0.4565	-2.344	H	6.85	0.8905	-1.686
H	6.8447	0.8801	-1.6991	H	6.5151	-0.5151	-0.6586
H	-0.1096	1.5477	3.0511	H	-0.1064	1.5365	3.0504
H	-1.3917	2.4114	2.2414	H	-1.3888	2.4028	2.2441
H	-1.6865	0.7743	2.8185	H	-1.6838	0.7655	2.8179
H	-3.4937	-3.3807	-0.7339	H	-3.5097	-3.3784	-0.7282
H	-4.9993	-2.6485	-1.2665	H	-5.0149	-2.6416	-1.2558
H	-4.5287	-2.5167	0.4273	H	-4.5374	-2.5101	0.4362
H	-3.7043	-1.4617	-3.0551	H	-3.7207	-1.4604	-3.0505
H	-2.3858	-0.3614	-2.6864	H	-2.3973	-0.3644	-2.6862
H	-2.1601	-2.0991	-2.4639	H	-2.1772	-2.1027	-2.4631
H	-5.7621	1.8682	-0.8428	H	-5.7601	1.8842	-0.8515
H	-1.6132	-2.1511	3.2049	H	-1.7949	-1.4678	3.1119
**5-5**	X axis(Å)	Y axis(Å)	Z axis(Å)	**5-6**	X axis(Å)	Y axis(Å)	Z axis(Å)
C	-3.8705	1.4553	-0.7785	C	-3.865	1.4607	-0.7745
C	-4.4422	0.0428	-0.7701	C	-4.4435	0.051	-0.7743
C	-3.4366	-1.1156	-0.9041	C	-3.4432	-1.1113	-0.9136
C	-2.2294	-0.8647	0.0326	C	-2.2344	-0.8715	0.0239

C	-1.6345	0.5483	0.0387	C	-1.6361	0.5398	0.044
C	-2.7766	1.567	0.2686	C	-2.7749	1.561	0.2775
C	-1.6867	-1.8424	0.7838	C	-1.691	-1.8585	0.7625
C	-0.4399	-1.7476	1.5942	C	-0.4402	-1.7766	1.5688
C	0.4498	-0.5256	1.2807	C	0.4465	-0.5477	1.2733
C	-0.4073	0.7807	1.0145	C	-0.4143	0.7589	1.0283
C	1.5459	-0.7947	0.199	C	1.5424	-0.799	0.1869
C	2.4553	0.4785	0.0636	C	2.4524	0.4773	0.0671
C	1.6162	1.6008	-0.5341	C	1.6036	1.6046	-0.509
C	0.4373	1.9277	0.3763	C	0.4321	1.916	0.4156
C	2.6069	-1.8584	0.5803	C	2.5984	-1.8685	0.5557
C	3.8576	-1.4859	-0.2529	C	3.8623	-1.4745	-0.2458
C	3.6078	-0.0649	-0.8191	C	3.6052	-0.0549	-0.8211
C	3.0509	1.0057	1.4111	C	3.0601	0.9816	1.4179
C	0.9156	-1.2819	-1.1418	C	0.9155	-1.272	-1.1608
O	4.003	-2.3963	-1.3397	O	4.0529	-2.3986	-1.3119
C	4.8903	0.7618	-0.826	C	4.8831	0.7811	-0.8516
O	5.7504	0.6517	0.0481	O	5.9113	0.4294	-0.2729
C	5.0513	1.7587	-1.9449	C	4.8446	2.0816	-1.6126
O	0.1146	3.112	0.5082	O	0.1214	3.0991	0.5817
C	-0.8764	1.3004	2.4054	C	-0.8909	1.2525	2.4262
C	-4.1962	-2.4322	-0.6211	C	-4.209	-2.4257	-0.6375
C	-2.9568	-1.1682	-2.3673	C	-2.963	-1.1581	-2.3769
O	-5.6667	-0.1186	-0.7469	O	-5.6688	-0.1041	-0.7528
O	-4.8828	2.4149	-0.4873	O	-4.873	2.4246	-0.4831
O	-0.8606	-1.7317	2.9525	O	-0.8537	-1.7817	2.9296
H	-1.2739	0.7373	-0.9798	H	-1.2701	0.7362	-0.9711
H	1.0063	-0.3529	2.2115	H	1.0039	-0.3861	2.2057
H	3.2952	-0.1358	-1.8687	H	3.2933	-0.1343	-1.8701
H	-3.4907	1.6748	-1.7809	H	-3.48	1.6828	-1.7744
H	-3.2306	1.4235	1.2583	H	-3.2336	1.4119	1.2642
H	-2.3934	2.5936	0.2547	H	-2.3875	2.5862	0.2729
H	-2.1274	-2.8355	0.8086	H	-2.1323	-2.8516	0.7761
H	0.131	-2.6724	1.4591	H	0.13	-2.699	1.4154
H	2.2218	2.509	-0.6376	H	2.1993	2.5189	-0.6049
H	1.2343	1.3663	-1.5316	H	1.2152	1.383	-1.5068
H	2.2684	-2.8815	0.3826	H	2.2667	-2.888	0.3292
H	2.8437	-1.7959	1.6487	H	2.8183	-1.8324	1.6292
H	4.7593	-1.562	0.3631	H	4.734	-1.544	0.4133
H	3.6816	0.2663	1.9135	H	2.3025	1.2882	2.1438
H	3.6684	1.8953	1.2381	H	3.6882	0.2311	1.9069
H	2.2884	1.3171	2.1294	H	3.6869	1.8659	1.2545
H	0.2285	-0.5626	-1.5821	H	0.2371	-0.5446	-1.6012

H	1.6566	-1.4926	-1.9171	H	1.6612	-1.4863	-1.931
H	0.3731	-2.2227	-1.0032	H	0.364	-2.2089	-1.0319
H	4.2951	-3.2476	-0.9673	H	4.9848	-2.3211	-1.5888
H	5.0239	1.2391	-2.9059	H	4.4724	2.878	-0.9656
H	4.2564	2.5051	-1.8963	H	5.8542	2.333	-1.95
H	6.0161	2.2648	-1.8494	H	4.2076	1.9843	-2.4952
H	-0.0325	1.3869	3.0998	H	-0.0502	1.3277	3.1259
H	-1.3195	2.3009	2.3563	H	-1.3356	2.253	2.3929
H	-1.6204	0.6399	2.8596	H	-1.6363	0.583	2.8647
H	-3.5705	-3.3132	-0.804	H	-3.5885	-3.3089	-0.827
H	-5.0745	-2.5345	-1.2703	H	-5.089	-2.5194	-1.2858
H	-4.5567	-2.4701	0.4137	H	-4.5679	-2.468	0.3977
H	-3.8019	-1.3051	-3.0527	H	-3.8086	-1.2848	-3.0636
H	-2.4487	-0.2492	-2.6771	H	-2.4481	-0.2408	-2.6804
H	-2.259	-1.9988	-2.5252	H	-2.2708	-1.9925	-2.5398
H	-5.7389	1.9667	-0.6483	H	-5.7311	1.9789	-0.6407
H	-0.0737	-1.8471	3.5107	H	-0.0694	-1.9543	3.4768

Reference

(1) Frisch, M.J.; Trucks, G.W.; Schlegel, H.B.; Scuseria, G.E.; Robb, M.A.; Cheeseman, J.R.; Scalmani, G.; Barone, V.; Mennucci, B.; Petersson, G.A. *Gaussian 09*; Version 09; Gaussian, Inc.: Wallingford, CT, USA, 2009.
